# Quantitative approaches to understanding conformational flexibility in crystalline sponges

**DOI:** 10.1039/d6ce00383d

**Published:** 2026-10-19

**Authors:** Robert C. Carroll, Eleanor M. Soper, James E. Pearce, Roohollah Hafizi, Alexandra Longcake, Adam P. Cotterill, Neil J. Wells, Michael J. Hall, Michael R. Probert, David C. Harrowven, Graeme M. Day, Simon J. Coles

**Affiliations:** a School of Chemistry and Chemical Engineering, University of Southampton Southampton UK s.j.coles@soton.ac.uk; b School of Natural and Environmental Sciences, Newcastle University Newcastle upon Tyne UK

## Abstract

The crystalline sponge (CS) method has expanded the capabilities of single crystal X-ray diffraction (SCXRD) chemical structure determination and analysis to liquid or oily compounds. Guest molecules encapsulated within the porous metal–organic framework (MOF) have variable intermolecular interaction strengths, both with the pore and other guest molecules, and often exhibit multiple molecular conformations. Quantification and understanding of these features has previously proved challenging and thus limited the insights available from structural models. Through combination of the CS method, conventional SCXRD, Nuclear Magnetic Resonance spectroscopy, single-molecule and periodic DFT, and energy calculations based on electron density, we determine intra- and intermolecular energy changes across a spectrum of physical environments. We explore the MOF pore environment experienced by a systematic family of small organic molecules and showcase the tools available for quantification of these complex systems.

## Abstract 2

Crystal sponge molecular structure conformations have been compared to those from pure crystal, simulated and solution states.

## Graphical abstract

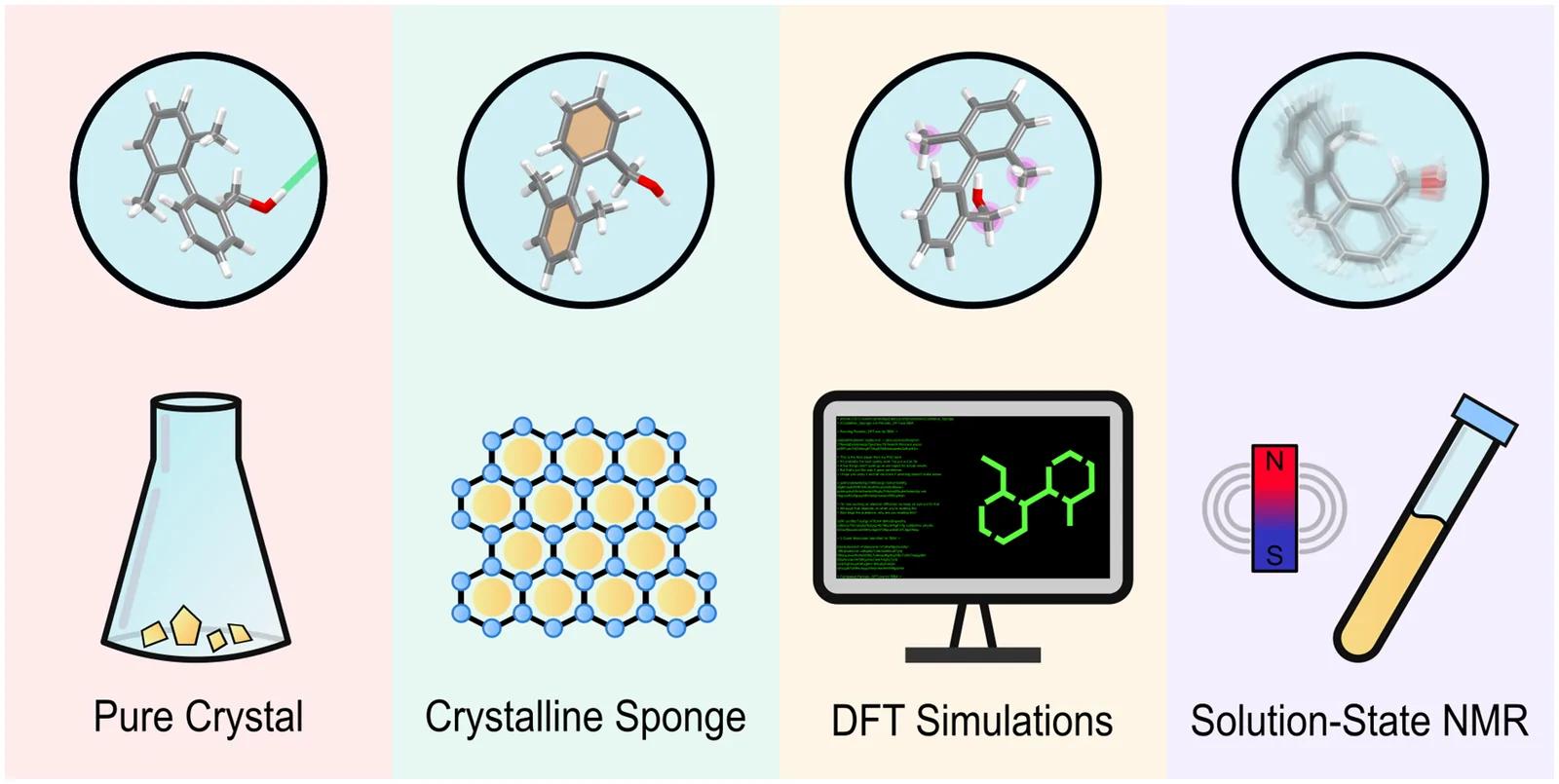

## Introduction

Single crystal X-ray diffraction (SCXRD) offers some of the most detailed and precise molecular level structural information available. The technique is regularly employed across an array of research areas, including characterising active pharmaceutical ingredients, understanding bond connectivity, and rationalising emergent physicochemical properties of materials.^1–3^ However, SCXRD analysis has the inherent limitation that samples must form relatively large and high-quality single crystals. For a wide variety of compounds, growing crystals suitable for diffraction study is extremely challenging and for many it is impossible under ambient laboratory conditions.

To address this issue, a range of advanced crystallisation methods have been developed to improve the compatibility of molecules for SCXRD.^4^ One of the most noteworthy of these developments is the crystalline sponge (CS) method, first published in 2013.^5,6^ This novel technique employs crystalline, porous, solvent-filled metal–organic frameworks (MOFs) as host scaffolds for smaller organic molecules. Small molecules diffuse into the crystalline framework where they can interact with host sites, thus enabling regular ordering of guest molecules throughout the 3D framework. Characterisation with SCXRD can then determine the molecular structure of the host–guest complex while avoiding direct crystallisation of the sample. This effectively expands SCXRD analysis to liquid or non-crystalline compounds.

Since its first publication, the [{(ZnX_2_)_3_(tpt)_2_·*x*(solvent)}_*n*_] host series (where X = Cl, Br, and I and tpt = tris(4-pyridyl)-1,3,5-triazine) has received the most attention of any MOF for CS applications (Fig. 1). The widespread interest and applicability of this host are driven by the variety of intermolecular interactions available for stabilising guest molecules within the pore. Encapsulated guest molecules commonly form aromatic⋯aromatic interactions with electron deficient tpt ligands, hydrogen bond with host nitrogen and halide atoms, or simply rely on van der Waals contacts with the interpenetrated framework. This variety of possible contacts results in compatibility of the host framework with a broad range of guest molecules exhibiting diverse chemical functionalities.

**Fig. 1:**
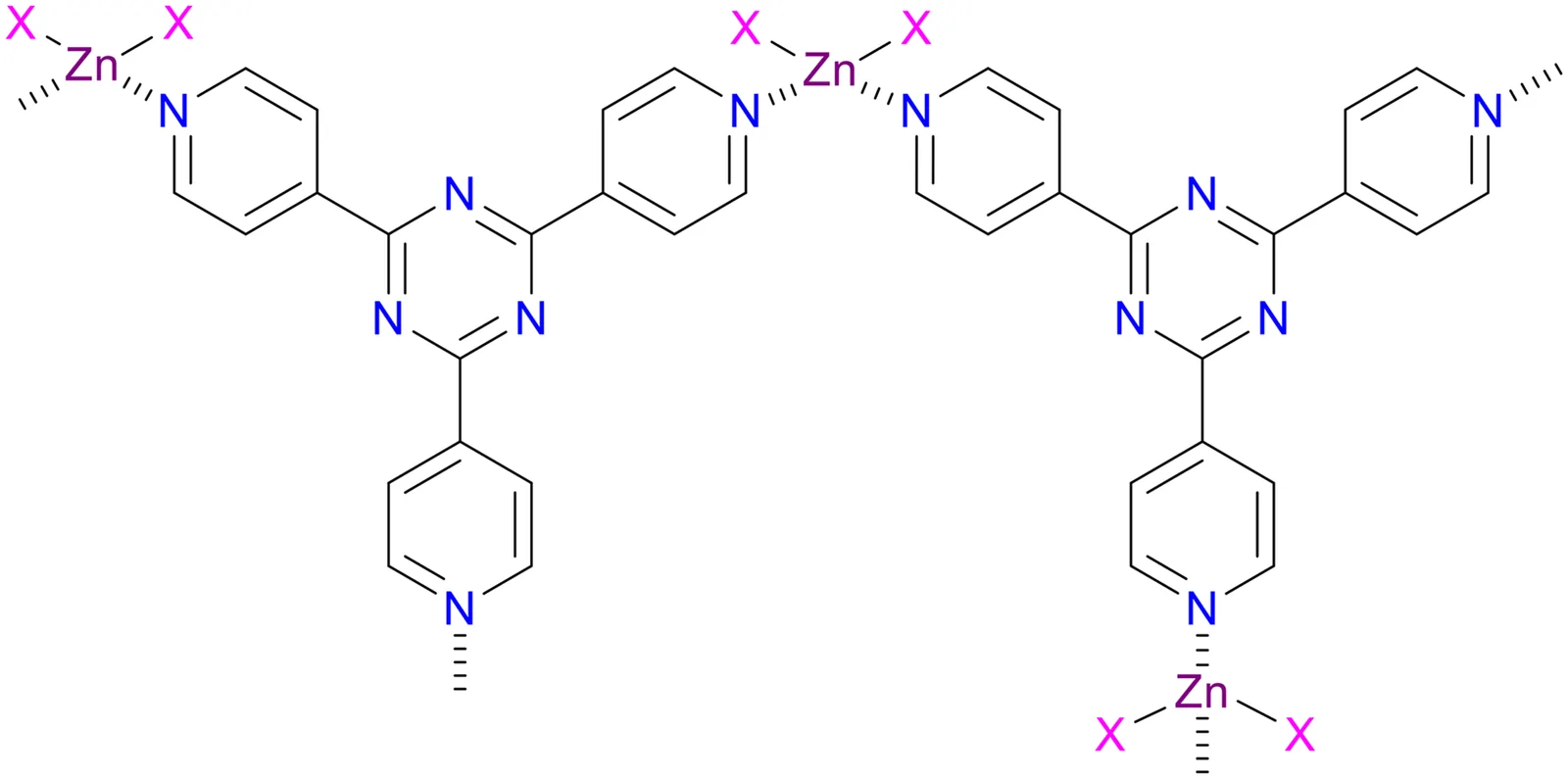
Molecular structure of [{(ZnX_2_)_3_(tpt)_2_·*x*(solvent)}_*n*_] crystalline sponge host.

To date, some of the most notable applications of the CS method include nanogram-scale analysis of natural products, combination with high-performance liquid chromatography (HPLC) for analysis of human pharmaceutical metabolites, and tracking the progress of chemical reactions *in situ*.^7–10^ In parallel, numerous studies have driven the development of the CS process, including optimisation of experimental procedures, fine-tuning of associated hardware, and systematic studies to better understand fundamental behaviours of guest molecules.^6,11–15^ The development of new CS frameworks has also attracted significant interest, with new host materials increasingly being developed for specific applications.^16–22^ Further information on the principles and development of CS analysis is summarised in two comprehensive reviews.^23,24^

Although the CS method offers the potential application of SCXRD analysis to non-crystalline samples, it cannot provide details of the analyte guest solid-state packing arrangement. This is a consequence of partially occupied guest exchange sites, singular guest molecules occupying pores, and the mixture of host–guest and guest–guest interactions adopted by molecules within the MOF cavities. Insights into solid state packing are crucial for engineering the solid state, particularly the development of new medical treatments, where packing motifs and intermolecular interactions are known to affect the stability and efficacy of pharmaceutical formulations.^25^

Whilst it is not possible to obtain comparable data for analyte solid-state packing *via* the CS method, recent work has shown that CS guest molecules can adopt lower energy molecular conformations, on average, than would normally be observed in pure organic crystal structures.^26^ Therefore, CS experiments can offer simultaneous molecular structure determination and relaxed conformational insights, where intramolecular energies can dominate over intermolecular packing forces.

This study further develops understanding of the CS pore environment through comparison of conformational energies for a related family of small organic molecules. These molecules have been structurally characterised *via* solution-state NMR, conventional SCXRD, and the CS method. The breadth of experimental data is complemented by single-molecule and periodic DFT simulations, which provide quantification of intramolecular energetics and insights into the influence of the CS framework on the conformation of guest molecules. Furthermore, intermolecular interaction energies based on electron density are quantified for the first time for CS and organic-solid state forms. The partitioning of energetic contributions is enabled by the PIXEL software, and therefore reliable comparison of these differing environments is possible.^27,28^ Overall, the combination of quantitative experimental and computational methods enables assessment of behaviour across a ‘spectrum of conformational flexibility’ where analyte molecules experience a variety of intra- and intermolecular forces. This work also showcases the tools available for quantification of these complex systems.

## Methodology

### Compound library

The compounds selected for conformational investigation are derivatives of the benzyl biaryl alcohol (BBA) family, illustrated in Fig. 2. This family of molecules has formed the basis of two systematic CS studies, where analytes 1, 3, 4, and 7 were previously characterised by the CS method.^14^

**Fig. 2:**
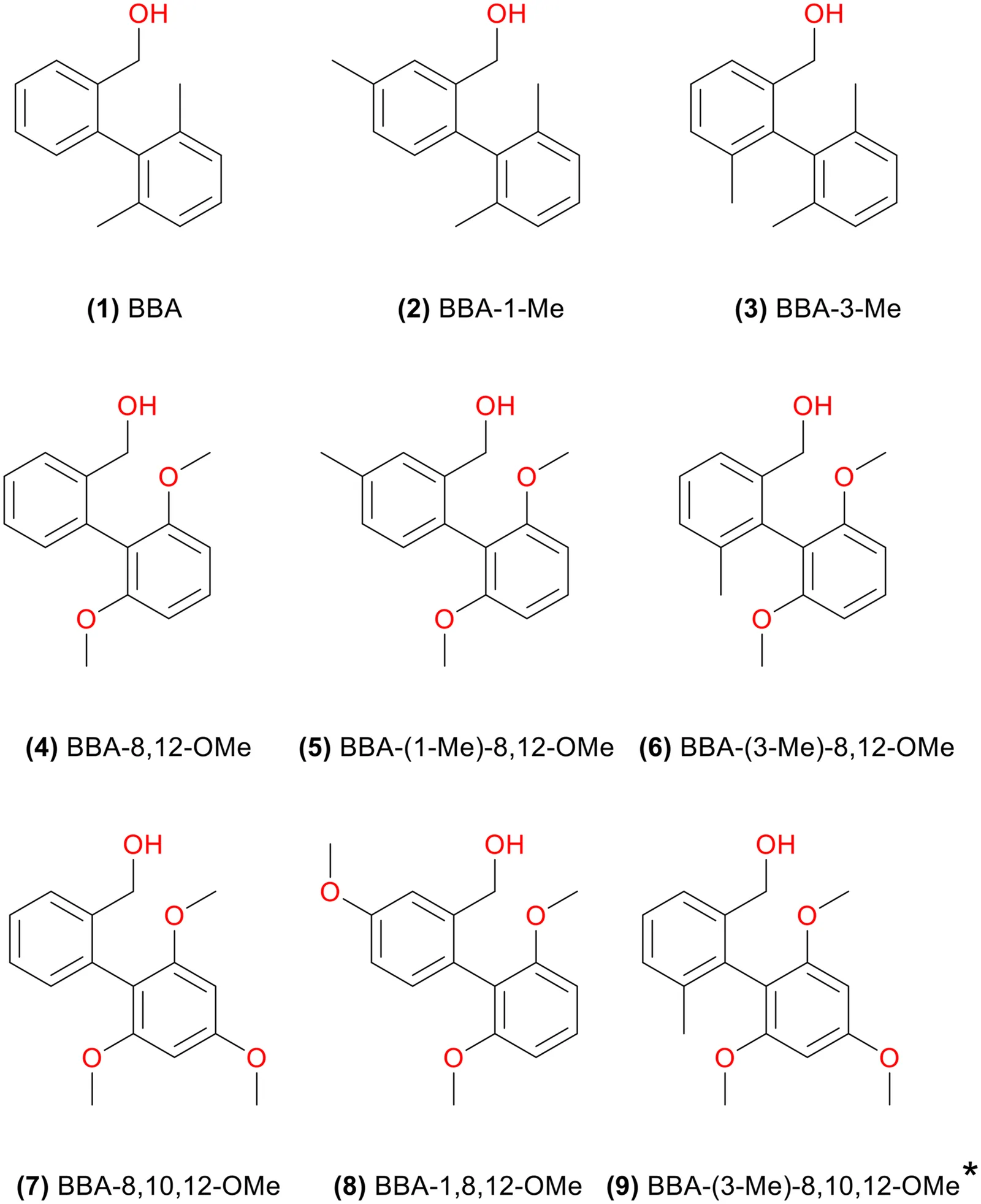
Benzyl biaryl alcohol (BBA) molecules for conformational analysis. *Analyte 9 used for NMR analysis only.

The BBA molecules are good candidates for this study because of synthetic control over functionalisation, which enables assessment of energetic trends in relation to guest molecule size and flexibility.^29^ These molecules are also compatible with multiple experimental analytical techniques, and their relatively simple molecular structures make computational conformer searches more amenable.

### Experimental methods

CS host crystals were synthesised *via* the layered diffusion method before exchange of nitrobenzene (C_6_H_5_NO_2_) templating solvent with cyclohexane (C_6_H_6_). The host material was provided by Merck Crystal-Do, Darmstadt and prepared as described in the literature.^8,30^ Guest exchange procedures likewise followed previous literature protocols and were carried out iteratively to ensure optimum exchange conditions for each analyte material. This was also informed by extensive screening of the BBA materials undertaken for previous studies.^15^ Final parameters used for guest exchange are shown in SI section S1.

CS SCXRD experiments were carried out on a Rigaku 007HF diffractometer using Cu-K_α_ radiation (*λ* = 1.54184 Å), equipped with Varimax confocal mirrors, UG2 goniometer, and HyPix 6000HE/HyPix Arc-100 detectors. All crystallographic structures were solved with SHELXT v2014/5, modelled with Olex2 v1.5, and refined with SHELXL v2016/6. These procedures match our previous systematic studies, and additional details can be found in SI section S1 alongside crystallographic data for novel CS structures presented in this study.

Due to being oily or poorly crystalline synthesis products, some compounds required encapsulated nanodroplet crystallisation (ENaCt) for characterisation by conventional SCXRD.^31^ ENaCt is a high throughput method offering greater control over crystallisation conditions.^32,33^ Further details of instrumentation, crystallisation conditions, and crystallographic data for ENaCt analysis are shown in SI sections S1.5 and S1.6. This enabled characterisation of all nine analytes with conventional SCXRD and formed the experimental basis for this study with CS crystallographic data.

NMR spectra were recorded on a Bruker AVIIIHD500 FT-NMR spectrometer equipped with a BBFO Cryoprobe. Samples were dissolved in deuterated dichloromethane (CD_2_Cl_2_) and experiments were conducted at 298 K, 273 K, and 243 K. Chemical shifts for proton spectra are reported on the delta scale in ppm and were referenced to residual solvent references or an internal TMS reference. All NMR spectra and their corresponding tabulations are detailed in SI section S8.

### Preparation of the dataset for computational analysis

CS crystal structures are typically more disordered than conventional crystal structures. This can arise in both the framework and the guest molecules (including solvent) present in the framework pores. Guest molecules can be present in low occupancy, for reasons such as being found in multiple positions, multiple orientations, disordered with solvent, or not being present in the same location in every pore. Consequently, the experimental crystal structure cannot be directly used for computational study. To account for the disorder and to ensure that the structure used for calculations is as realistic as possible, these structures must be considered on a case-by-case basis, often in multiple configurations. All computational methods used for modelling crystal structures assume full (100%) occupancy of all atoms. This simplification of the crystallographic model and its consequences are discussed in greater detail in the Results and discussion section. Details for considering these factors and the suitability of CS structures for computational study are described in the complementary publication by Soper *et al.* and presented in SI section S2.^26^

### Intramolecular energetics and conformer searches

To evaluate molecular geometry and energetics for the conformations in the observed crystal structures, the experimental structures must first be optimised using periodic DFT to remove errors or experimental artefacts which may remain after structure refinement. All periodic and single molecule DFT calculations were performed with CRYSTAL17 (ref. 34) using the POB-TZVP basis set,^35,36^ B3LYP functional,^37^ and D3 dispersion correction.^38^ To account for the basis set superposition error (BSSE) that arises in finite basis sets, the geometric counterpoise (gCP) correction is implemented in all calculations.^39^ Further details for the calculation of intramolecular energetics, including geometry optimisation, conformational searches, and stability of conformers are detailed in SI section S2 and the complementary publication.^26^

Symmetry-independent target guest molecules were extracted from the DFT-optimised experimental CS structures and used for calculation of intramolecular strain energy (Δ*E*_strain_), conformational energy (Δ*E*_conf_), and total relative energy (Δ*E*^intra^_total_), as shown in relation to the conformational energy landscape in Fig. 3. Δ*E*_strain_ is an energetic measure of the distortion of the molecule away from its nearest stable gas phase geometry and is calculated from the energetic difference between the crystalline molecular geometry (red point in Fig. 3) and its local energy minimum conformer (LM), obtained by unconstrained optimisation of the isolated experimental molecule.

**Fig. 3:**
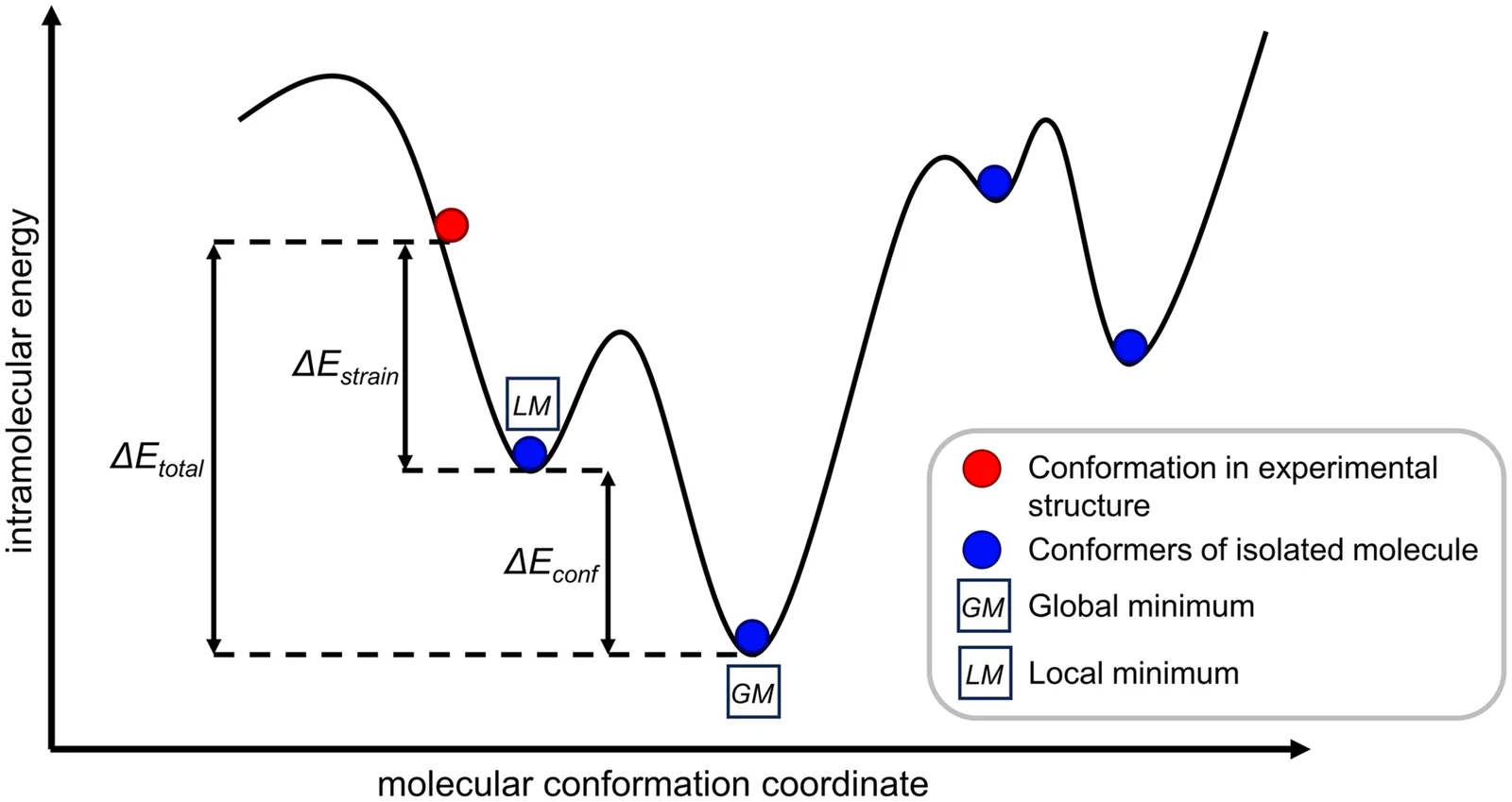
Schematic of a generic conformational energy surface: the molecular energy *versus* a general conformational degree of freedom. Reproduced under CC-BY license.^26^

To generate conformer ensembles for each molecule studied (Fig. 2), the CREST conformer search method^40^ was used as implemented in the CSPy programme.^41,42^ Multiple CREST searches were generated from diverse starting positions to ensure thorough sampling and to fully search the conformational landscape. Any duplicates from the combined searches were removed *via* clustering based on torsion angles. The unique conformers were optimised with DFT-D3, applying the same computational parameters as described for crystal structures, before removing duplicates again to generate the final DFT conformer landscape. Geometric comparisons of the sets of conformers were performed using root mean square deviation of atomic positions to identify the local conformer of the isolated molecule from the observed crystal structures in the conformer landscape. Δ*E*_conf_ describes the energetic difference between the global minimum conformer (GM) and the local energy minimum conformer (LM) corresponding to the experimental molecular geometry.

The total relative energy of the isolated molecule from the experimental crystal structure, Δ*E*^intra^_total_, represents the energy difference between the molecular conformation in the optimised experimental structures (*E*^mol^_crystal_) and the most stable gas phase molecular geometry (*E*_GM_), and can be found as described in eqn (1):

1 Δ*E*^intra^_total_ = Δ*E*_strain_ + Δ*E*_conf_ = *E*^mol^_crystal_ − *E*_GM_

### PIXEL intermolecular energy calculations

Intermolecular interaction energy calculations were carried out with the PIXEL method *via* the MrPIXEL interface for CSD Mercury software.^27,28,43,44^ The PIXEL method is a semi-empirical approach that can provide similar insight to DFT calculations but requires considerably less compute time.^45^

The method partitions molecules into cubic volumes of electron density, termed pixels, which are derived from experimental crystallographic structures. Pairwise calculations on individual pixels enable the quantitative determination of Coulombic (*E*_COU_), polarisation (*E*_POL_), and dispersion (*E*_DISP_), intermolecular interaction energies. Repulsion (*E*_REP_) energies are calculated from the overlap of electron density and summation of these terms allows the total intermolecular interaction energy (*E*^inter^_TOT_) to be calculated (eqn (2)).

2 *E*^inter^_TOT_ = *E*_POL_ + *E*_COU_ + *E*_DISP_ + *E*_REP_

The quantification enabled by this method provides greater insight into the nature of whole molecule contacts and can provide insight into emergent physicochemical properties. This study focuses on symmetry-independent guests within the CS host due to the tendency of unique molecules to adopt different conformations.

PIXEL calculations for CS structures required hydrogen-only geometry optimisations and normalisation to neutron scattering distances due to the difficulty of reliably determining hydrogen positions within the CS pores. The complementary calculations on conventional SCXRD structures did not require optimisation but hydrogen bond lengths were similarly normalised to neutron scattering distances. CS structures were modelled as fragments of chemically reasonable moieties for compatibility with MrPIXEL software, a technique previously employed for gas adsorption studies with MOF materials.^46^ Full details of this approach and benchmarking studies conducted to assess viability with a CS system are detailed in SI section S4. All atoms in the structure are assumed at full (100%) occupancy for PIXEL calculations. Gaussian input files were generated with the DGDZVP basis set and MP2 method due to their integration into Gaussian software and compatibility with framework iodine atoms.^47^ Further explanation of the PIXEL settings employed, as well as the manual workflow adopted for application to MOF host–guest systems, can be found in SI section S3.

### SCXRD and CS molecule labelling scheme

Characterisation of molecules across multiple analytical techniques requires clear nomenclature for investigation. In this study the shorthand in Table 1 is used to distinguish between molecules characterised by conventional SCXRD (pure organic crystals) and CS data.

**Table 1:** Shorthand nomenclature for discussion of conventional SCXRD and CS data

Structure	Molecule site	Shorthand
Pure crystal	A	P_A_
	B	P_B_
Crystalline sponge	A	CS_A_
	B	CS_B_
	C	CS_C_

## Results and discussion

### Total intramolecular and intermolecular energies

Analysis must first address the variation of guest conformations and intramolecular energies observed for the selection of molecules in the study. The influence of CS encapsulation on guest conformation can first be assessed through comparison of total relative (Δ*E*^intra^_total_) energies. Δ*E*^intra^_total_ plots and tabulation for all symmetry-independent molecules from pure crystal and CS structures are displayed in Fig. 4.

**Fig. 4:**
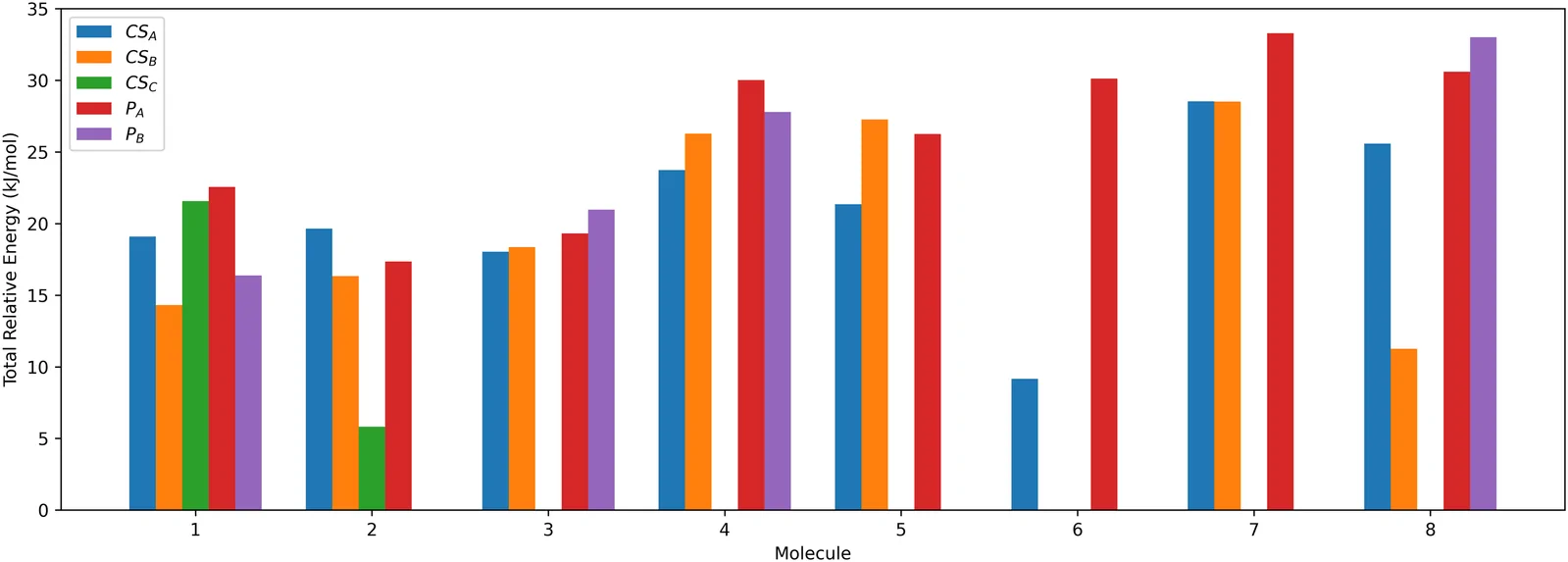
Δ*E*^intra^_total_ for symmetry-independent biaryl molecules in pure organic (left) and CS (right) structures. Molecules are labelled numerically according to the scheme provided in Fig. 2.

In Fig. 4, it is apparent that all guest molecule conformations are affected by encapsulation within the CS host, as all CS guests exhibit non-zero values for Δ*E*^intra^_total_. This behaviour is readily rationalised by the influence of intermolecular interactions: guests visible *via* diffraction study must form reliable host–guest or guest–guest contacts for positional stability throughout the crystalline framework. These influence molecular geometry and consequently Δ*E*^intra^_total_.

The Δ*E*^intra^_total_ values observed for biaryl CS guests range from 5.8 to 28.6 kJ mol^−1^ and fall within the range of our previous study (Fig. 5) which explored a structurally diverse set of guest molecules.^26^ Although overall a similar range of energies is observed between the studies, the chemical functionalities and molecular structures explored are not analogous, which suggests some consistency of host confinement effects regardless of the guest. However, while the biaryl molecules do fall within the range of intramolecular energies previously explored, those functionalised with polar substituents are often higher in energy and do not adopt structures related to their gas phase global energy minimum conformer (SI section S4).

**Fig. 5:**
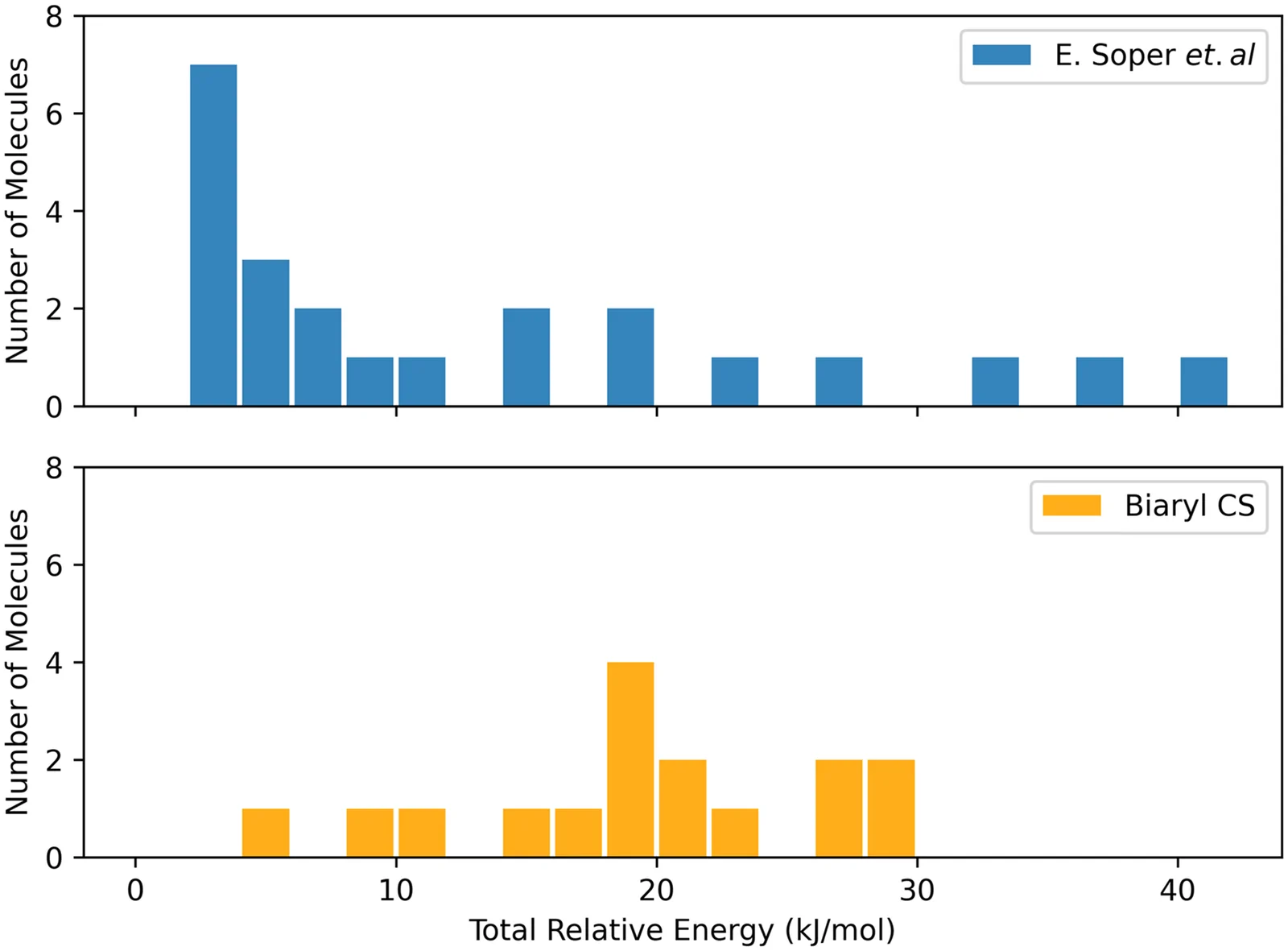
Histogram comparing Δ*E*^intra^_total_ of biaryl molecules within CS structures with those previously studied by Soper *et al.*^26^

The variability of intramolecular energies, exhibited by symmetry-independent guests within individual models, is also noteworthy because it showcases the diverse influence of host encapsulation on molecular conformation of chemically identical molecules. Comparison of Δ*E*^intra^_total_ between CS and pure organic structures (Fig. 4) highlights that biaryl molecules inside the MOF host adopt more relaxed geometries on average than their pure crystal counterparts, 19.7 and 25.6 kJ mol^−1^ respectively. However, biaryl molecules within the CS structures possess a far wider range of Δ*E*^intra^_total_ values, tabulated in SI section S6.

Structural overlays of experimental and local energy minimum conformers, extracted from both geometry optimised structures, are displayed with gas phase global energy minimum conformers in Fig. 6 for a direct visual comparison of molecular conformation. This highlights that rotation of the benzyl alcohol arm (–CH_2_OH) significantly influences the relative energy for less conformationally complex biaryl molecules. In compound 1, the two unique simulated local energy minimum conformers differ primarily in their hydroxyl positions which results in a 7.2 kJ mol^−1^ difference of Δ*E*^intra^_total_ between the observed structures. In contrast, compound 7 exhibits deviations in aryl–aryl torsion angle alongside the variety of –CH_2_OH and –OCH_3_ positions. While there is little overall difference in Δ*E*^intra^_total_ for CS_A_ and CS_B_ for compound 7, examination of their Δ*E*_conf_ and Δ*E*_strain_ values (SI Fig. S12) indicates that their greater conformational flexibility provides alternative routes for the biaryl molecules to balance intramolecular energy in the solid-state. These energetic components are investigated systematically for all biaryl molecules below.

**Fig. 6:**
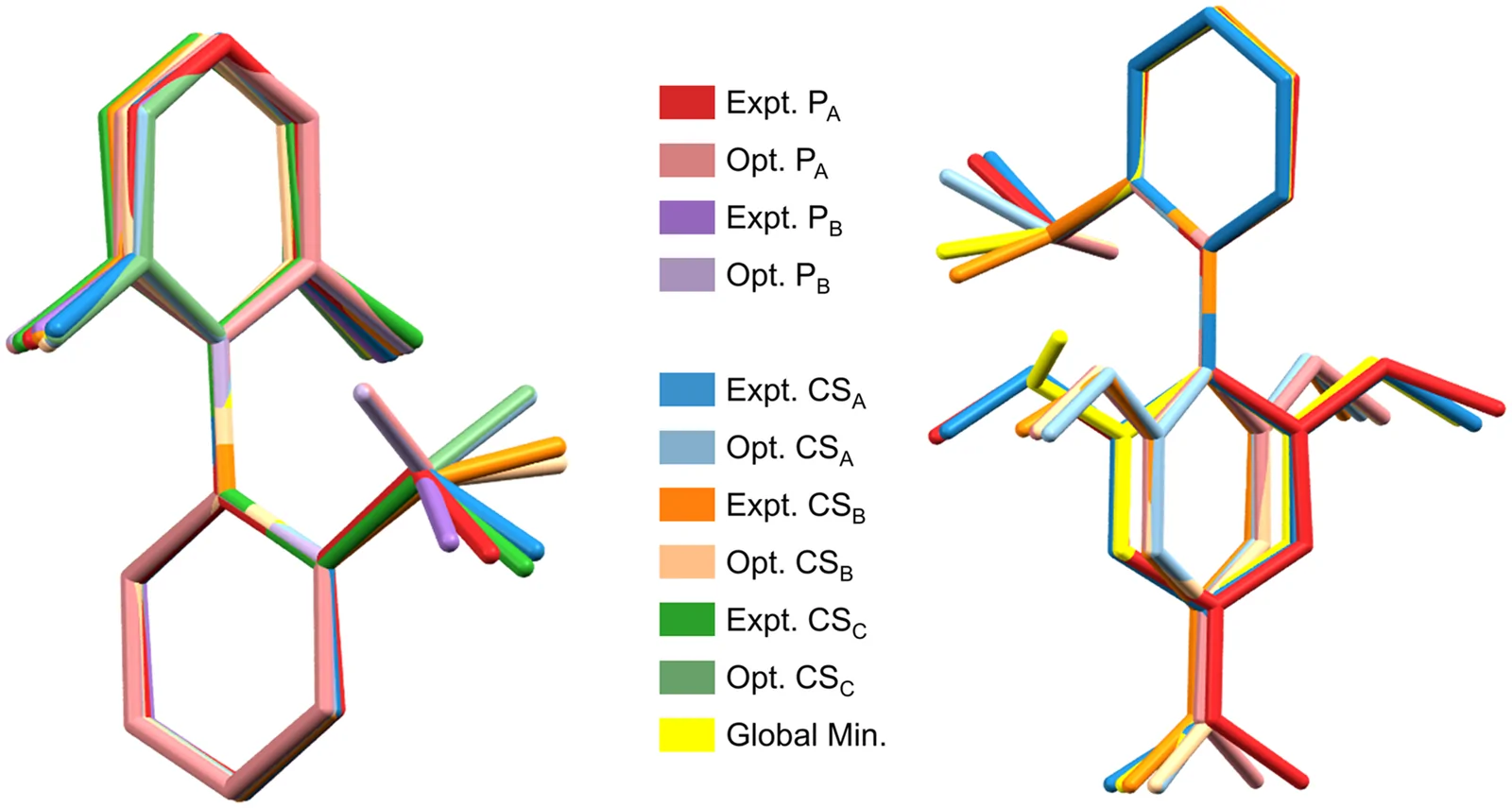
Structural overlays of experimental (expt.), optimised (opt.), and gas phase global energy minimum (global min.) molecular conformations for (left) BBA and (right) BBA-8,10,12-OMe.

Overall, biaryl molecules in pure organic and CS structures experience very similar total intermolecular interaction energies (*E*^inter^_TOT_) on average (−136.9 and −135.6 kJ mol^−1^ respectively) but there is much greater variation for CS guests, as showcased in Fig. 7.

**Fig. 7:**
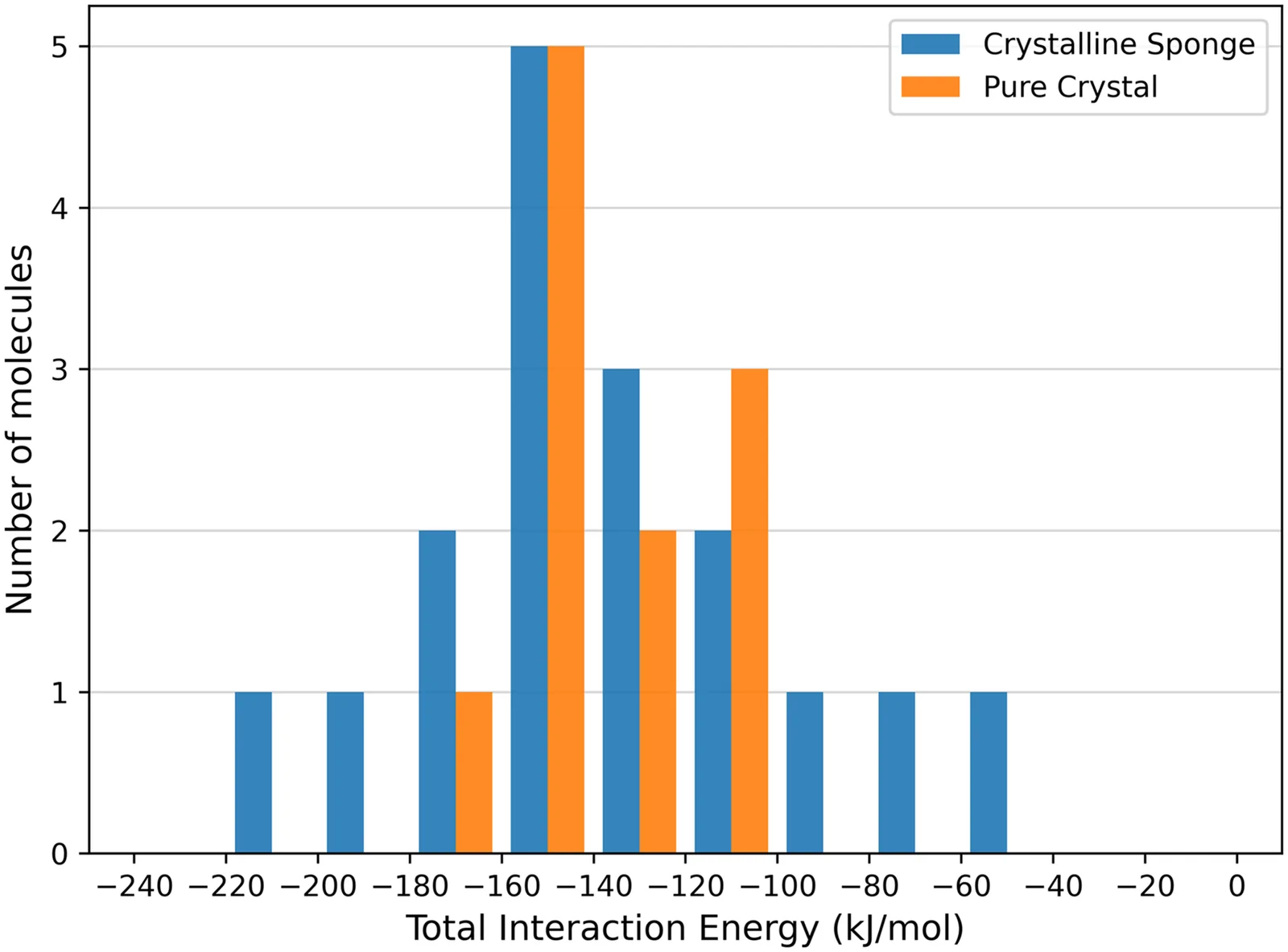
Histogram showing the distribution of *E*^inter^_TOT_ for biaryl molecules in conventional SCXRD and CS structures.

Notably, *E*_REP_ forms a greater proportion of intermolecular interactions on average for CS guests in comparison to pure organic structures (Fig. 8). This may explain why CS guest molecules do not adopt significantly more relaxed conformations than their pure organic crystal forms: although the CS host is a porous material, the interpenetrated framework results in a relatively confined space which imposes strong repulsive forces on guests.

**Fig. 8:**
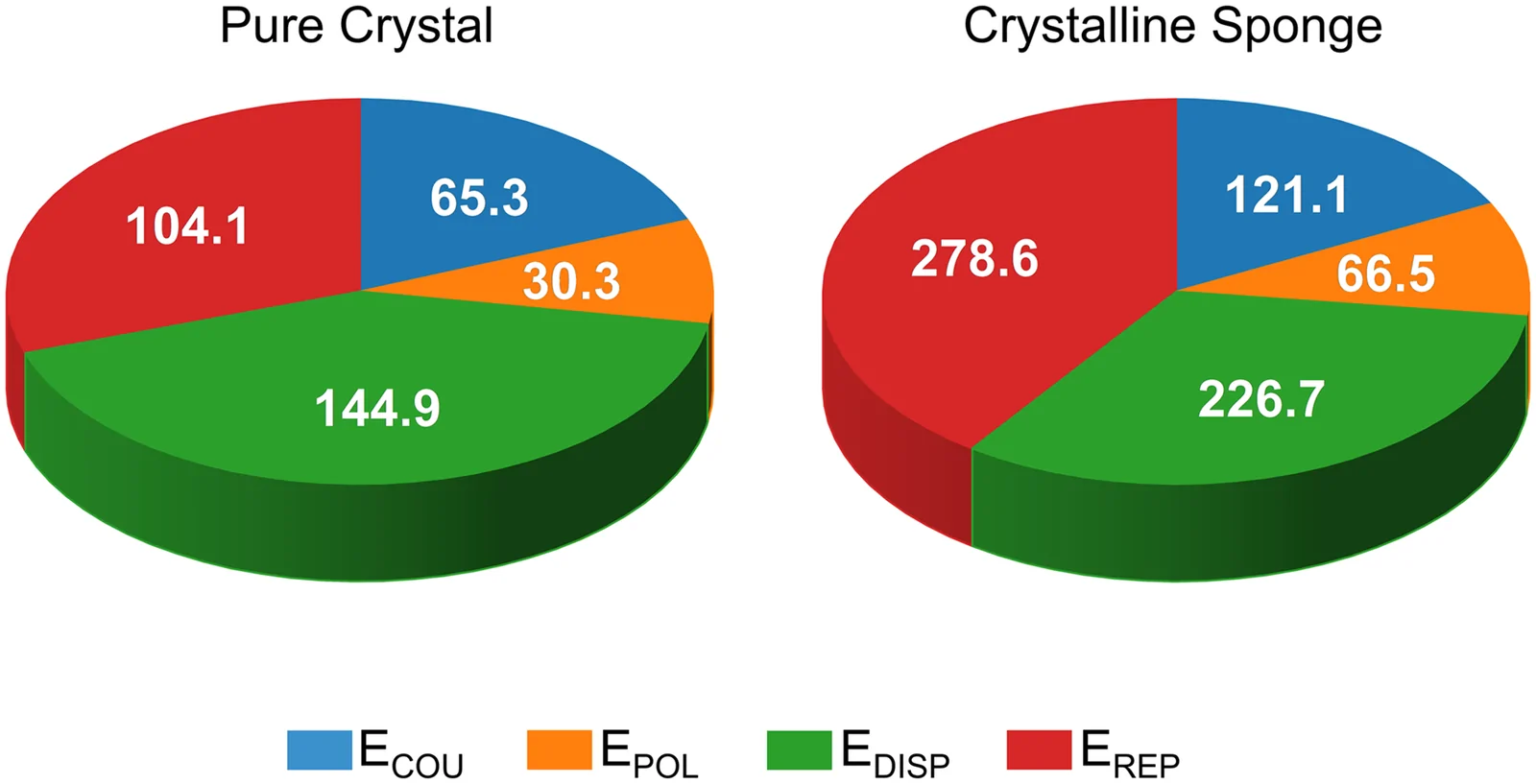
Pie chart for comparison of average intermolecular interaction energies for pure crystal (left) and CS structures (right), values are quoted in kJ mol^−1^.

The influence of molecular size, conformational flexibility, and number of hydrogen bond acceptors on Δ*E*^intra^_total_ can also be assessed by examining Fig. 4. Pure crystal structures exhibit a positive correlation with the larger, more flexible molecules possessing higher relative molecular energies (Δ*E*^intra^_total_). This also corresponds to a positive correlation with *E*_DISP_ and *E*^inter^_TOT_ (SI section S7.1), revealing that the larger and more conformationally complex biaryl compounds tend to adopt stronger interactions in pure crystalline forms. This is primarily driven by aromatic⋯aromatic contacts which define the packing motifs, illustrated in Fig. 9.

**Fig. 9:**
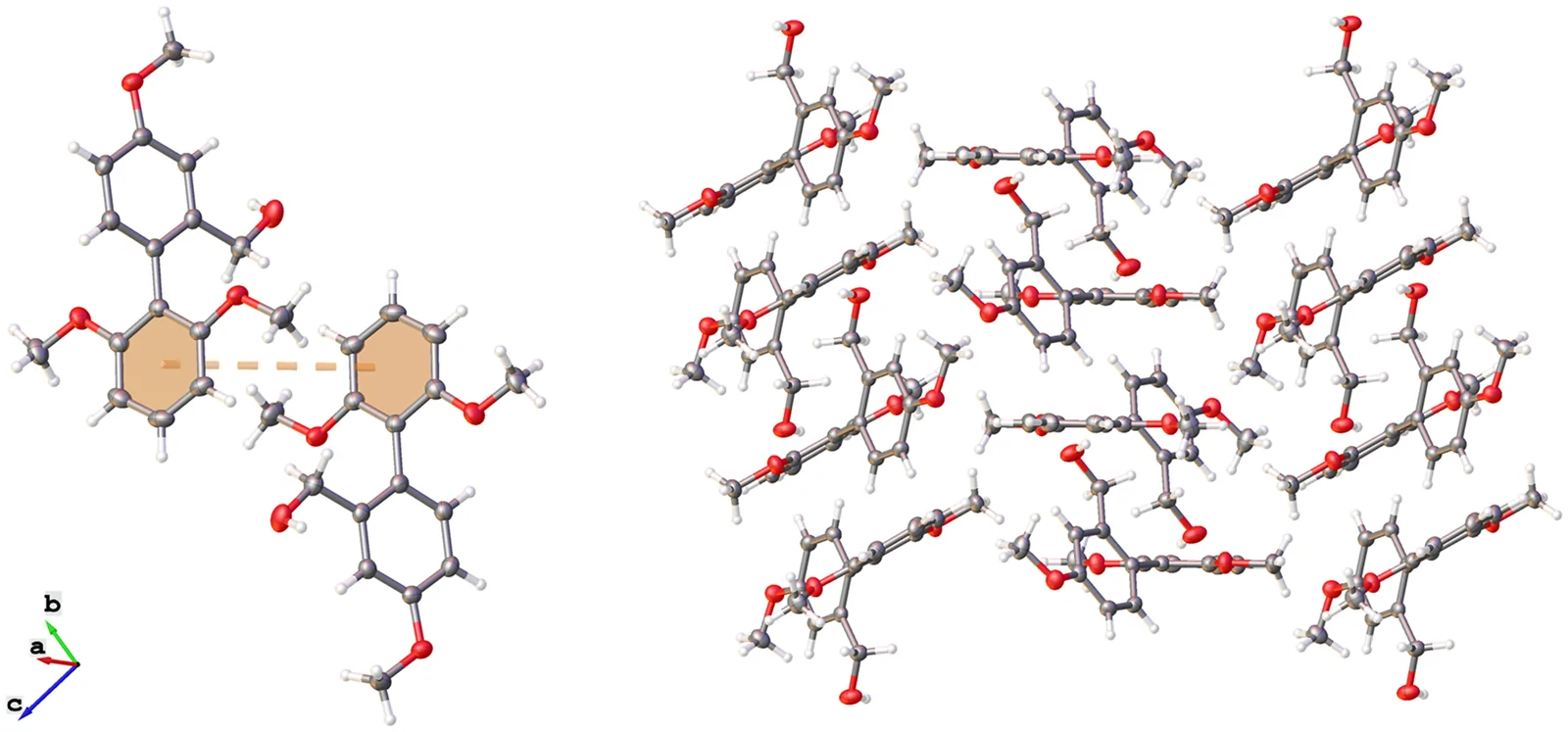
Aromatic⋯aromatic interaction of 8 in pure organic crystalline form (left) and its extended packing structure viewed down the *b*-axis (right).

In contrast, CS structures do not exhibit the same trends, with some of the largest and most flexible molecules adopting lower Δ*E*^intra^_total_ (Fig. 4). The inconsistent values observed for Δ*E*^intra^_total_ in CS structures emphasise that the influence of encapsulation on guest geometry is dependent not only on molecular structure but also the nature of the exchange site in the pore.

To provide further context on Δ*E*^intra^_total_ values for CS guest molecules, ^1^H-NMR studies were undertaken to probe their conformation in solution state. Preliminary NMR studies were performed on all compounds in this study and annotated ^1^H-NMR spectra with full assignments can be found in SI section S8. Analyte 9 represents a biaryl derivative with significant steric bulk and enables assessment of viability for solution-state conformational studies and is therefore a representative example for discussion. However, it was not included in the wider conformational study due to its incompatibility with the CS method.

Experiments conducted at variable temperatures (298 K, 273 K, and 243 K) were unable to differentiate between proton environments for *ortho*-substituted methoxy groups. This prevented investigation of proton couplings and determination of a three-dimensional geometry. Further two-dimensional solution-state NMR studies were therefore not undertaken.

The inability to distinguish between substituents was attributed to their similar electronic environments and constant rotation, or ‘wobbling’, around the central aryl–aryl bond which resulted in averaged signals. This is supported by previous computational investigations on conformational energy plots of substituted biphenyl compounds, which indicate a range of energetically accessible aryl–aryl torsions.^48^

While these results do not provide additional quantitative insight into intramolecular energies, they do provide qualitative context to the different environments experienced by molecules in solution-state and within the CS host. No single conformer can be isolated from NMR study, but CS structures commonly present 1 to 3 isolated conformers and orientations. This indicates that while the CS guest molecules do reside in a relatively large pore, the observed host–guest complex has a far closer resemblance to the solid-state where molecules are locked into more consistent conformations.

### Intermolecular energy components and flexibility

Although CS guests are not comparable to molecular solution-state behaviour, the relationship between guest molecular energies and surrounding environments can still be explored. To provide further insight into the observed conformations, host–guest and guest–guest intermolecular interactions in CS pores are separated and discussed in relation to conformational restraints imposed by the host framework.

Firstly, biaryl analytes in the CS favour interaction with the larger, electron-deficient host framework, evidenced by host–guest interactions contributing four times as much to *E*^inter^_TOT_ than guest–guest contacts on average (−103.3 *versus* −26.3 kJ mol^−1^) as shown in Fig. 10. This highlights that although neighbouring CS guests may be in suitable proximity for intermolecular contact within the pores, many molecules do not adopt complementary positions to maximise guest–guest interactions. This plot also showcases that CS host–guest intermolecular interaction contributions are weaker than pure crystal *E*^inter^_TOT_ and can vary more significantly. This likely has some relation to the variety of exchange sites adopted by guests within CS structures.

**Fig. 10:**
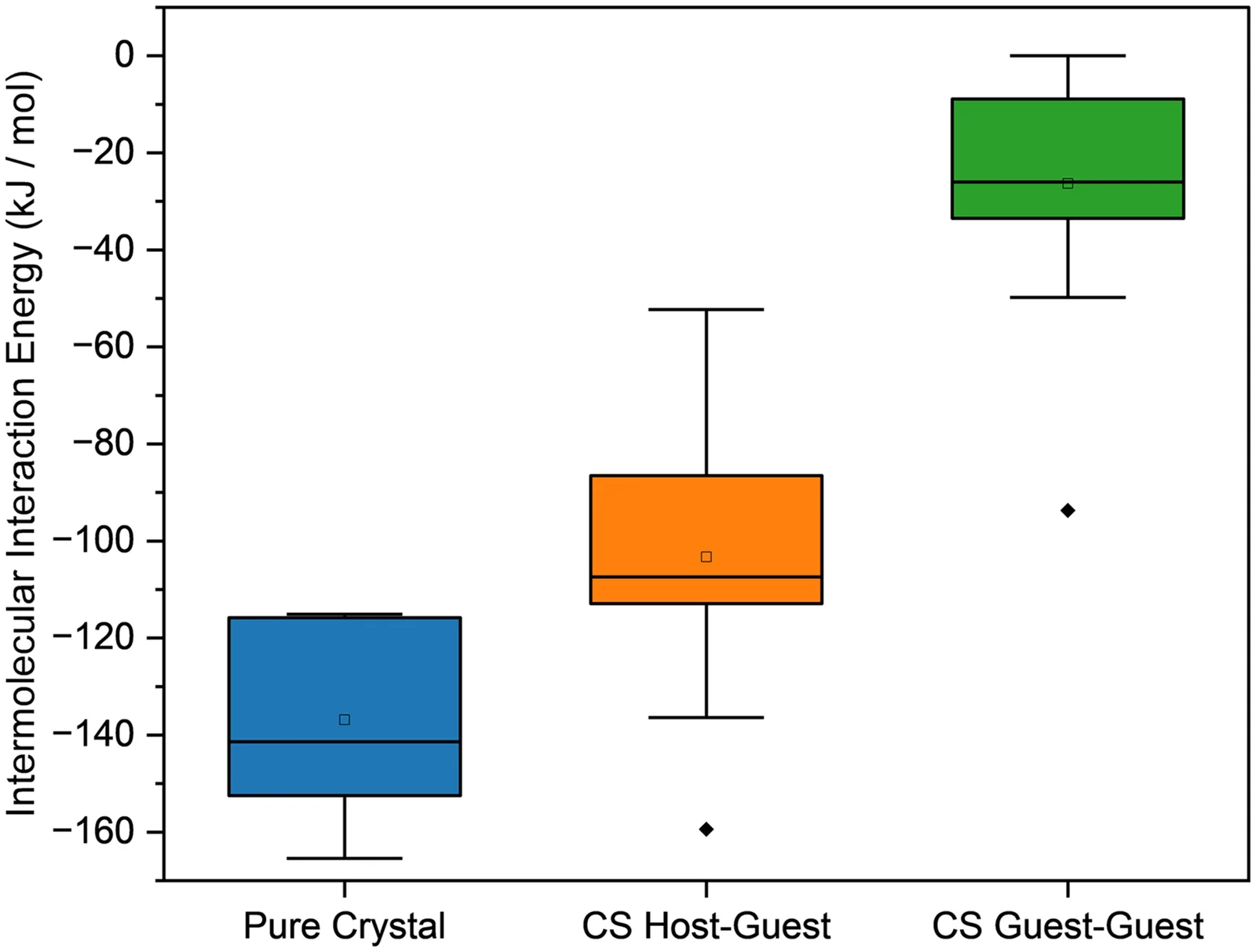
Box plot for pure crystalline, CS host–guest, and CS guest–guest intermolecular interaction energy contributions. Boxes are shown with 25–75% range within 1.5 interquartile range. Outliers are represented by filled diamond. The mean for each dataset is represented by unfilled squares.

To examine the influence of guest molecule flexibility on the observed intra- and intermolecular energies, the number of rotatable bonds present (excluding methyl groups) is compared to the intramolecular energy components (Δ*E*^intra^_total_, Δ*E*_conf_, and Δ*E*_strain_) as displayed in Fig. 11 and Table 2.

**Fig. 11:**
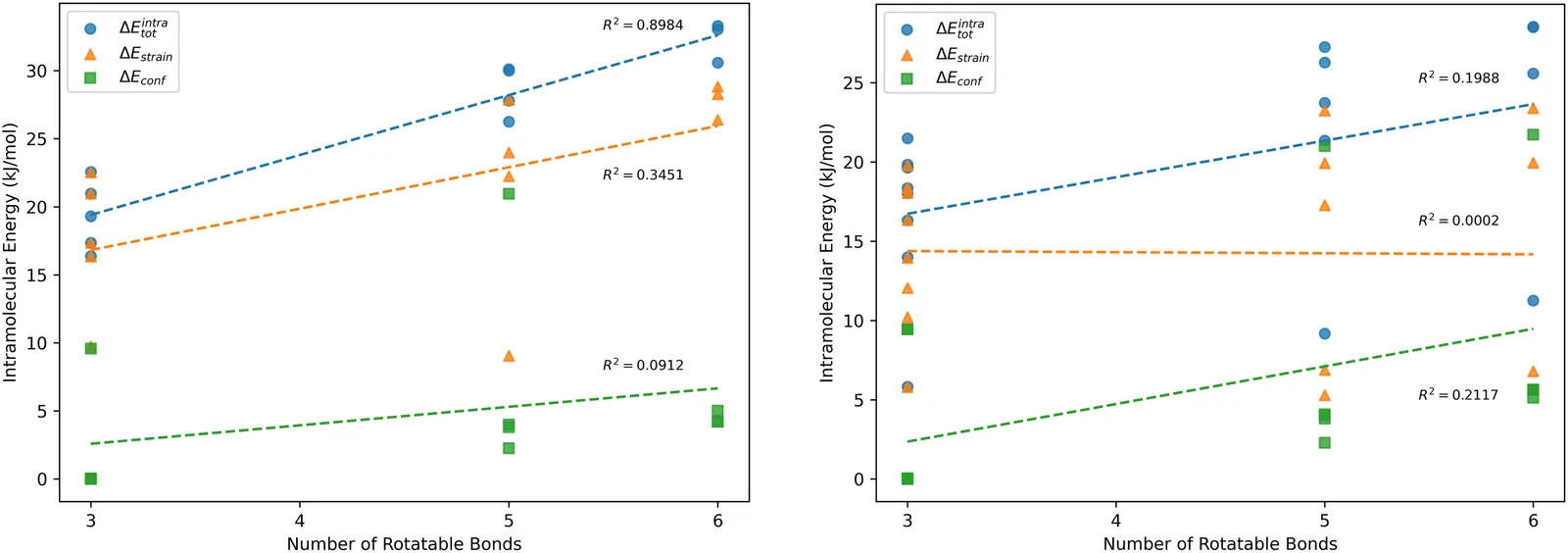
Intramolecular energy components plotted as a function of guest rotatable bonds (methyl not included) for conventional SCXRD (left) and CS structures (right).

**Table 2:** Linear regression statistics for intramolecular energy components plotted with respect to the number of rotatable bonds (methyl not included)

		*R* ^2^-Value	Standard error	*t*-Stat	*P*-Value
Pure crystal	Δ*E*_total_	0.8984	0.4680	9.4019	0.00002
	Δ*E*_strain_	0.3541	1.3251	2.2954	0.04460
	Δ*E*_conf_	0.0921	1.3565	1.0015	0.34020
Crystalline sponge	Δ*E*_total_	0.1988	1.1951	1.9293	0.07283
	Δ*E*_strain_	0.0002	1.2659	0.0537	0.95784
	Δ*E*_conf_	0.2117	1.1828	2.0069	0.06313

The trends for Δ*E*_strain_ reflect those previously identified for Δ*E*^intra^_total_: pure organic structures exhibit a positive correlation with increasing molecular size and conformational flexibility, but there is too much variability in CS structures to reliably assess a linear trend. In comparison, Δ*E*_conf_ has significant variability for both pure organic and CS structures, which indicates the relative conformer adopted in the solid-state is dependent on the unique surrounding environment as well as the size and flexibility of the molecule.

The complementary plots which include methyl bonds are shown in SI section S9 and match the trends from Fig. 11 and Table 2. Another factor to consider is conformational restriction of guest molecules within the pore; ligands have limited flexibility while retaining framework connectivity and crystallinity, which will define the pore spaces, and subsequently molecular geometry, which guests can occupy.

Overall, this behaviour emphasises the complex network of intermolecular interactions and the key role which the flexible CS framework plays in observed guest conformation and corresponding relative energies. For the biaryls, this environment has shown a tendency for more consistent intramolecular energies but will likely have a dependency on both the host and guest structures being studied.

Variation in guest flexibility can also be examined with respect to the intermolecular interaction energy components, shown in SI section S10. CS guests with greater flexibility adopt more favourable *E*_COU_ and *E*_POL_ interaction energies, supported by low *P*-values from linear regression analysis (0.0482 and 0.0547 respectively). However, for the subset of biaryl molecules examined, flexibility is confounded by the addition of methoxy groups and hydrogen bond acceptors, and as such this trend would not be expected for all CS guests. Notably, this trend provides greater context to our previous systematic study which investigated a variety of chemical functionalities substituted on the biaryl backbone.^14^

Here it was observed that addition of substituents with a propensity for intermolecular contacts did not always increase the number of interactions. However, quantification of these interactions in this current work has indicated that it does in fact result in more energetically favourable contacts.

### Intra- and intermolecular energy correlations

Direct comparison of intramolecular and intermolecular energy components enables a greater overview of analyte behaviour across multiple environments, as displayed in Fig. 12. Firstly, pure crystal structures exhibit strong negative correlations of *E*_DISP_ and *E*^inter^_TOT_ with Δ*E*^intra^_total_, confirmed by low *P*-values from linear regression analysis (0.0024 and 0.0005 respectively). This indicates that molecules with higher relative intramolecular energies (Δ*E*^intra^_total_) adopt the most favourable interactions, and this is correlated with molecular size and flexibility for the pure crystal structures investigated within the study.

**Fig. 12:**
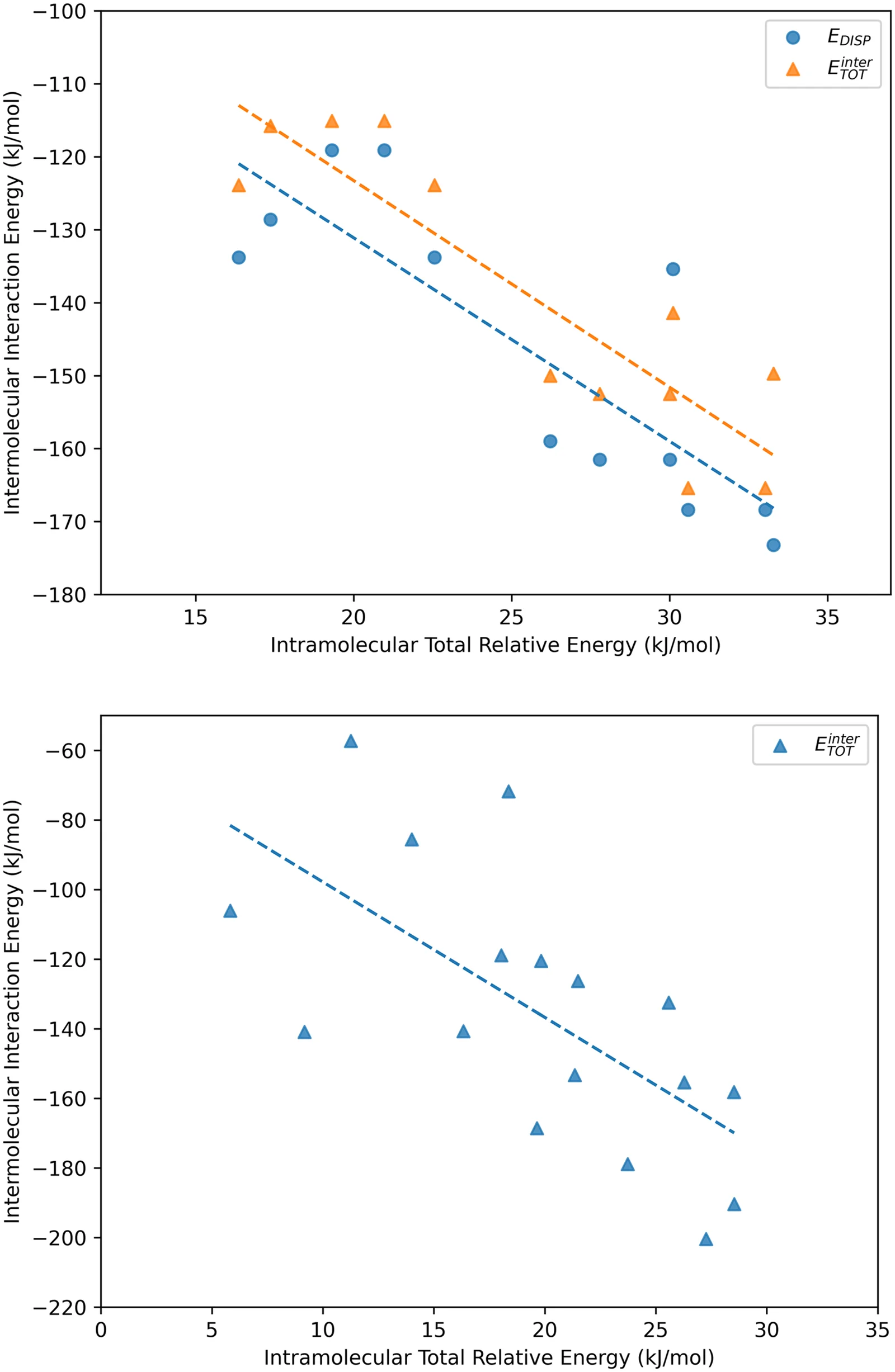
Scatterplot of Δ*E*^intra^_total_*versus E*_DISP_ and *E*^inter^_TOT_ for pure crystal structures (top) and Δ*E*^intra^_total_*versus E*^inter^_TOT_ for CS structures (bottom). Each data point represents a symmetry-independent molecule.

Similarly, CS structures exhibit a strong negative correlation between *E*^inter^_TOT_ and Δ*E*^intra^_total_, highlighting that stronger intermolecular interactions within the MOF host result in higher relative intramolecular energies for guests. While this overall trend is observed for *E*^inter^_TOT_, no individual interaction energy component displays a statistically significant trend, suggesting that no singular interaction type is dominant for all guest molecules. Instead, intermolecular interaction energies have varied composition for specific analytes and exchange sites but consistently result in higher energy conformations for guests adopting the most stable intermolecular interactions.

The relationship of *E*^inter^_TOT_ with Δ*E*_conf_ and Δ*E*_strain_ for pure crystal and CS structures is shown in SI section S11. Neither of these plots present any statistically significant trends with respect to intermolecular interactions. Therefore, the strength of intermolecular contact is not found to influence the relative conformer adopted or the intramolecular strain of the biaryl molecules.

Division of intermolecular interaction energies into host–guest and guest–guest contributions enables further detailed analysis, tabulated in SI section S11.2. Host–guest interactions provide the most significant contribution to the observed correlation between *E*^inter^_TOT_ and Δ*E*^intra^_total_. This result is readily rationalised due to the primary role host–guest contacts play in guest positional stabilisation (Fig. 10). There is also a negative correlation for host–guest *E*^inter^_TOT_ and Δ*E*_conf_ (SI section S11.2) which further supports the influence of the guest adsorption site on the relative conformer adopted.

Conversely, evaluation of Δ*E*_strain_ with respect to both host–guest and guest–guest interaction energies does not present a clear trend. This suggests that guests which are more weakly bound to the host framework may adopt more strained conformations to maximise favourable interactions. It could also indicate that Δ*E*_strain_ has a greater dependence on guest–guest contacts, but the variability of interaction motifs and strengths prevents consistent influence even for a set of structurally similar molecules.

## Conclusions

Through combination of a range of experimental and computational methods, we have quantified changes in intra- and intermolecular energies for a related family of molecules across a variety of environments. These quantitative results exhibit good agreement with expected behaviours from experimental observations. It has been shown that encapsulation of analytes within the CS framework influences guest intramolecular energies, however, there is suitable conformational freedom within the pores for molecules to adopt lower energy conformers than their pure crystal forms.

The intramolecular energy of biaryl molecules in pure crystalline solid-state is directly influenced by molecular size, conformational flexibility, and number of hydrogen bond acceptors. In contrast, biaryl guests in crystalline sponge cavities have a greater dependency on the adopted exchange site and surrounding pore environment.

Quantification of intermolecular interaction energies confirms that analytes favour host–guest interactions, regardless of exchange location. However, these host–guest interactions are often weaker than the interactions observed between analytes in pure crystal structures.

The strength of intra- and intermolecular energies is found to be correlated for biaryl guests within the CS framework, with stronger intermolecular interactions resulting in higher energy conformations. The lack of trend for intermolecular interaction energies and relative molecular energetic components demonstrates the variability inherent in many CS structures (*i.e.* interactions can manifest in many different ways even for similar molecules). While no statistically meaningful trend is observed here, there will still be an impact on guest intramolecular energies and additional data with more varied molecular structures may improve understanding of these observations.

Direct interpretation of these results from the final crystallographic model is often difficult, or impossible, due to their complexity (overlapping solvent, disorder, symmetry sites). Importantly, quantitative insights into CS structures require similar analysis to that conducted here and identification of trends, even with a variety of tools, is still challenging. For further insights, Monte Carlo simulations which comprehensively explore the adsorption sites available to guest molecules are currently being undertaken. This will likely add another key piece of information for understanding and predicting behaviour in these complex systems.

## Author contributions

R. C. Carroll – methodology, investigation, analysis, writing. E. M. Soper – methodology, investigation, analysis, writing. J. E. Pearce – investigation. R. Hafizi – software, writing. A. Longcake – investigation. A. P. Cotterill – investigation. N. J. Wells – methodology, investigation. M. J. Hall – supervision. M. R. Probert – supervision. D. C. Harrowven – methodology, supervision. G. M. Day – methodology, supervision, writing. S. J. Coles – conceptualization, methodology, supervision, writing.

## Conflicts of interest

There are no conflicts to declare.

## Supplementary Material

CE-OLF-D6CE00383D-s001

## Data Availability

The data supporting this article have been included as part of the supplementary information (SI). Supplementary information: data collection and refinement strategies for crystallographic analysis, calculation and tabulation of intramolecular energetics, calculation and tabulation of intermolecular energetics, NMR spectra for all compounds, and comparison of intramolecular and intermolecular energy components. See DOI: https://doi.org/10.1039/d6ce00383d. CCDC 2341223–2341225, 2341231–2341234 and 2359637–2359640 contain the supplementary crystallographic data for this paper. Additional crystallographic data used in this study which has been published previously can be found at CCDC 2254203, 2254204, 2254207, 2254208, and 2341226. Further data, including optimised versions of crystal structures and conformers are available at https://www.pure.soton.ac.uk at https://doi.org/10.5258/SOTON/D4016.^49*a*–*k*^
