# Supplementary material for: Quantitative approaches to understanding conformational flexibility in crystalline sponges
Source: CrystEngComm. 2026 Oct 19. Online ahead of print. doi: 10.1039/d6ce00383d (PMC13678619; doi:10.1039/d6ce00383d)
Supplement: Supplementary file 1 [file CE-OLF-D6CE00383D-s001.pdf]

Supplementary Information (SI) for CrystEngComm.
This journal is © The Royal Society of Chemistry

Electronic Supplementary Information

Quantitative approaches to understanding conformational flexibility in crystalline sponges

Robert C. Carroll*[a], Eleanor M. Soper[a], James E. Pearce[a], Roohollah Hafizi[a], Alexandra Longcake[b], Adam P. Cotterill[a], Neil J. Wells[a], Michael J. Hall[b], Michael R. Probert[b], David C. Harrowven[a], Graeme M. Day[a], and Simon J. Coles*[a]

[a]School of Chemistry and Chemical Engineering, University of Southampton, United Kingdom

[b]School of Natural and Environmental Sciences, Newcastle University, United Kingdom

Contents

1. Crystallographic Analysis 3
   1.1. Crystalline Sponge Data Collection and Refinement Strategy 3
   1.2. Crystallographic Model Restraints and Constraints Diagrams 5
   1.3. Conventional SCXRD Data Collection 6
   1.4. Crystalline Sponge Experimental Conditions 6
   1.5. Conventional SCXRD Crystallisations 7
   1.6. Crystallographic Tables, Diagrams, and Notes 8
2. Calculation of Intramolecular Energetics 19
   2.1. Methods 19
   2.2. Definitions 19
3. PIXEL Calculations 21
   3.1. Workflow 21
   3.2. Defining Coordination Spheres and Framework Fragments 22
   3.3. Coordination Sphere Benchmarking Tabulation 25
4. Intramolecular Energies 27
   4.1. Conventional Single-component Crystal Structures 27
   4.2. Crystalline Sponge Structures 28
   4.3. Comparison of CS Structures to Pharmaceutical Subset 30
   4.4. Comparison of Conventional Crystal Structures to Pharmaceutical Subset 31
5. Geometric Changes Influencing Intramolecular Energy for Biaryl Molecules 34
6. Intermolecular Interaction Energies 35
   6.1. Conventional SCXRD 35
   6.2. Crystalline Sponge 36
      6.2.1. Overview 36
      6.2.2. Host – Guest and Guest – Guest Contributions 38
7. Distribution of Conformational and Strain Energies 40
   7.1. Conventional SCXRD 40
   7.2. Crystalline Sponge Structures 42
8. NMR Spectra and Tabulation 44
9. Guest Flexibility and Intramolecular Energies 65
10. Guest Flexibility and Intermolecular Energies 68
11. Intramolecular and Intermolecular Energy Plots 70
   11.1. Conventional SCXRD 70
   11.2. Crystalline Sponge 73

11.2.1. Overview 73
11.2.2. Host – Guest and Guest – Guest Contributions 76
12. References 82

1. Crystallographic Analysis

1.1. Crystalline Sponge Data Collection and Refinement Strategy

Single crystal X-ray diffraction experiments were carried out on Rigaku 007HF diffractometer using Cu-K_{α} radiation (λ = 1.54184 Å). Equipped with Varimax confocal mirrors, UG2 goniometer, and HyPix 6000HE / HyPix Arc-100 detectors. Data was collected to 0.83 Å resolution for consistency across all samples and strategies were calculated based on $I/\sigma > 15$ for the weakest reflections. CrysAlis^{Pro} software was used for calculation of experiment strategy and data reduction (data integration, empirical and numerical absorption corrections, and scaling).[1]

All structures were modelled with Olex2 v1.5, solved with SHELXT v2014/5, and refined with SHELXL v2016/6.[2–4] Attempts were made to model all non-hydrogen atoms anisotropically. However, low occupancy guests / solvents and overlapping sites prevented stable refinement for some. In these cases, rather than applying restraints to anisoptropic displacement parameters, the molecule was modelled isotropically with fixed displacement parameters (U_{iso} = 0.08). Hydrogen atoms were added with a riding model.

For determination of guest molecule occupancies, non-hydrogen elements were refined as isotropic atoms with a single, common free-variable (FVAR) and fixed thermal parameters (U_{iso} = 0.08). The guest occupancies were then fixed before anisotropic refinement to prevent expansion of thermal ellipsoids and erroneous assignment of residual electron density within the porous framework. Restraints / constraints were required for most guest molecules and are detailed for each dataset. Solvent molecules were found in the difference map and refined as restrained bodies (DFIX, DANG, SIMU, RIGU, and SADI). The considerable thermal motion of solvent within the pore results in the models for some of the averaged structures refining to include geometrically unrealistic distortions. During finalisation of crystallographic structure refinements, some high-intensity reflections were omitted when they possessed an [Error/esd] > 10.

The framework numbering scheme illustrated below in Figure S 1 was applied to each structure to assist rapid identification of host-guest interactions within the structures.

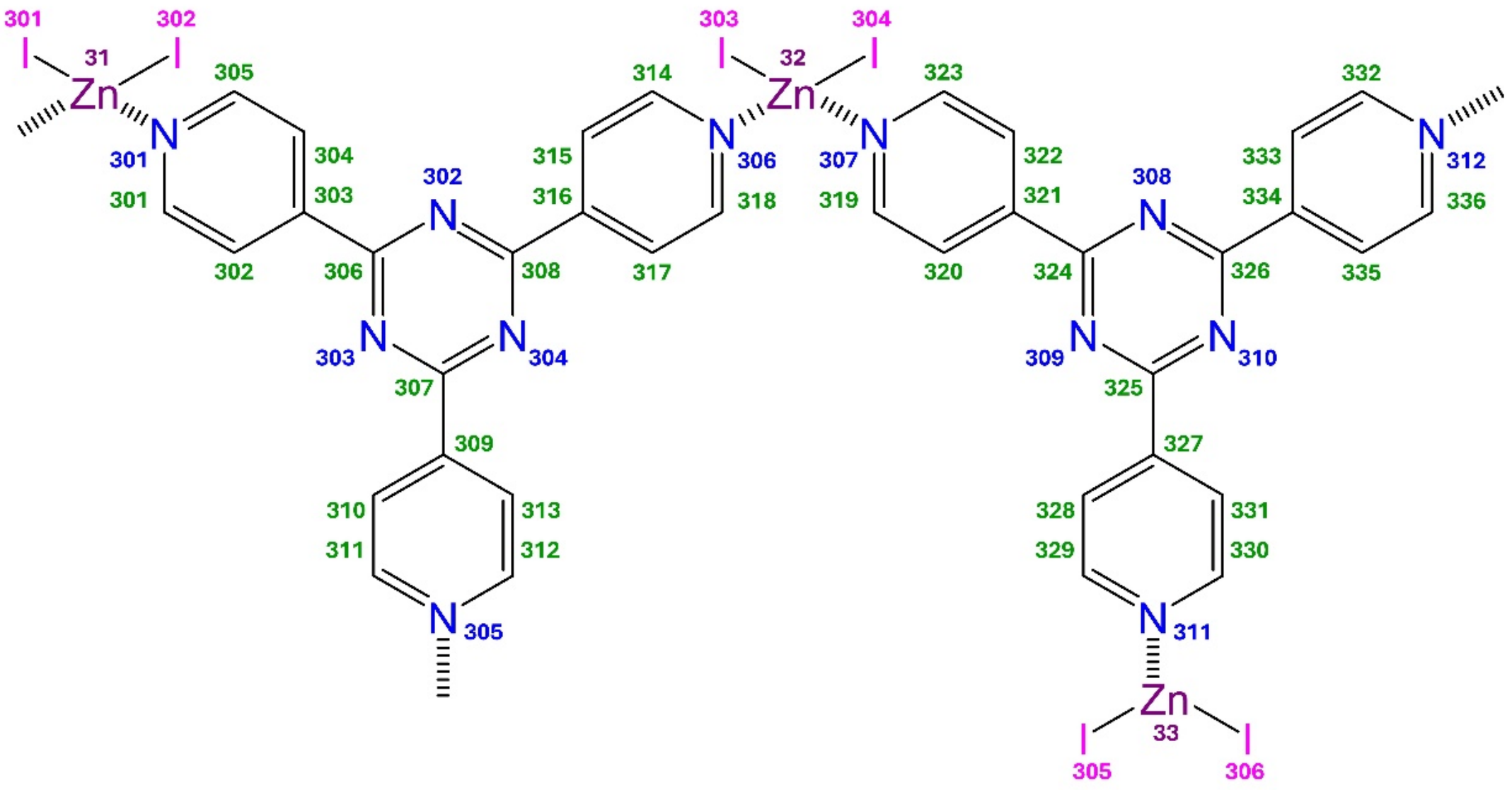

Figure S 1: Crystalline sponge host framework atom numbering scheme.

Incomplete exchange of analyte molecules during the inclusion process results in variation in guest or solvent content between individual cavities of the host framework. This commonly presents itself as lower average guest occupancy or overlapping electron density contributions from multiple molecules in the average crystallographic structure as determined by SCXRD. This can result in distortion of molecular conformations which is commonly treated with crystallographic restraints and constraints to match chemical intuition and prior knowledge of the guest analyte molecular structures. To reduce the influence of these crystallographic artefacts in subsequent simulations, overlapping molecules were separated into unique crystallographic structures for simplicity. This procedure is described in detail in the complementary publication by Soper et al., CrystEngComm, 2025.

1.2. Crystallographic Model Restraints and Constraints Diagrams

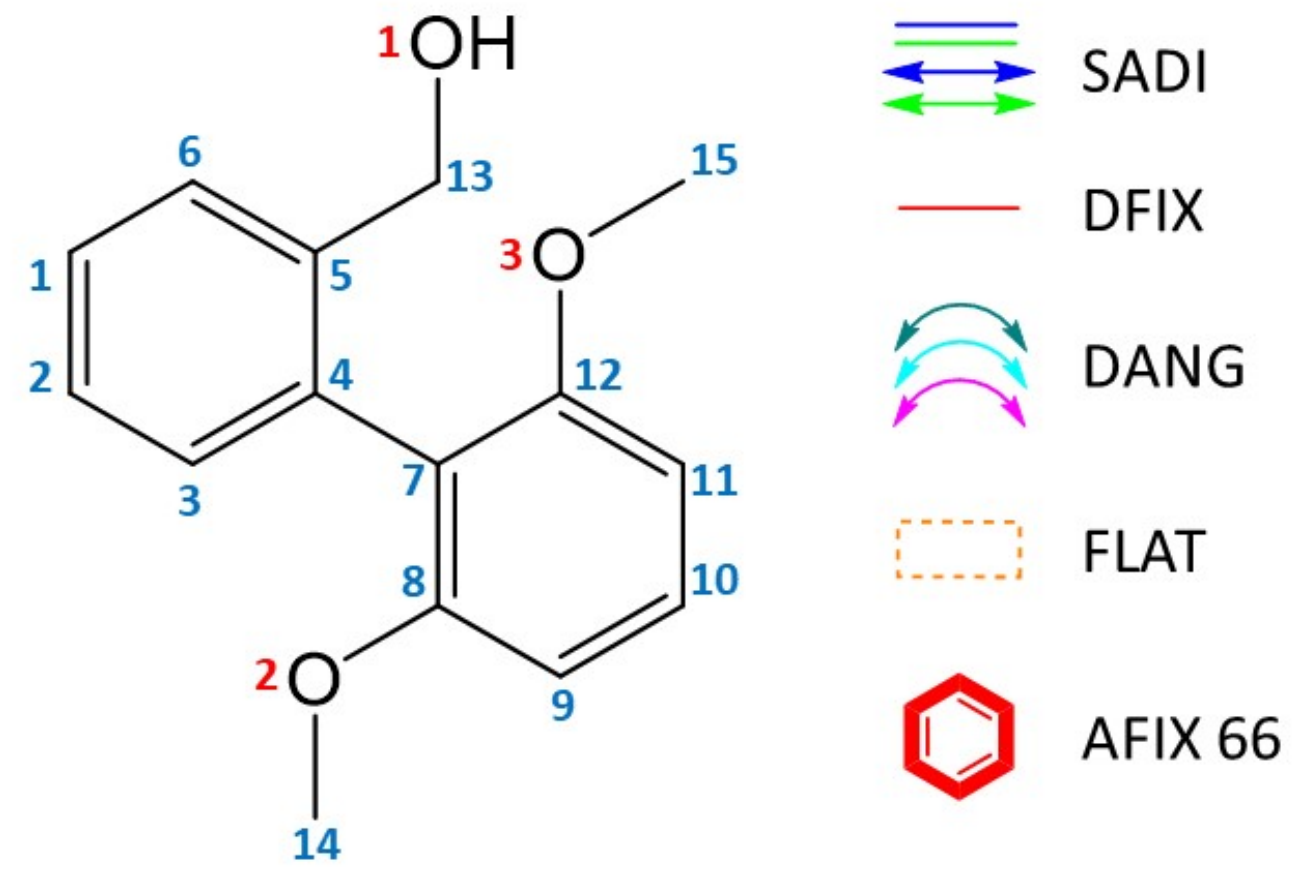

Figure S 2: BBA-8,12-OMe structure and crystallographic restraint representations.

From a practical perspective, any bond shown in black has had no geometric restraints or constraints applied, while any colouration or emboldening indicates application of crystallographic restraints or constraints. An example finalised diagram for BBA-8,12-OMe is illustrated with a text-based version of the restraints in Figure S3.

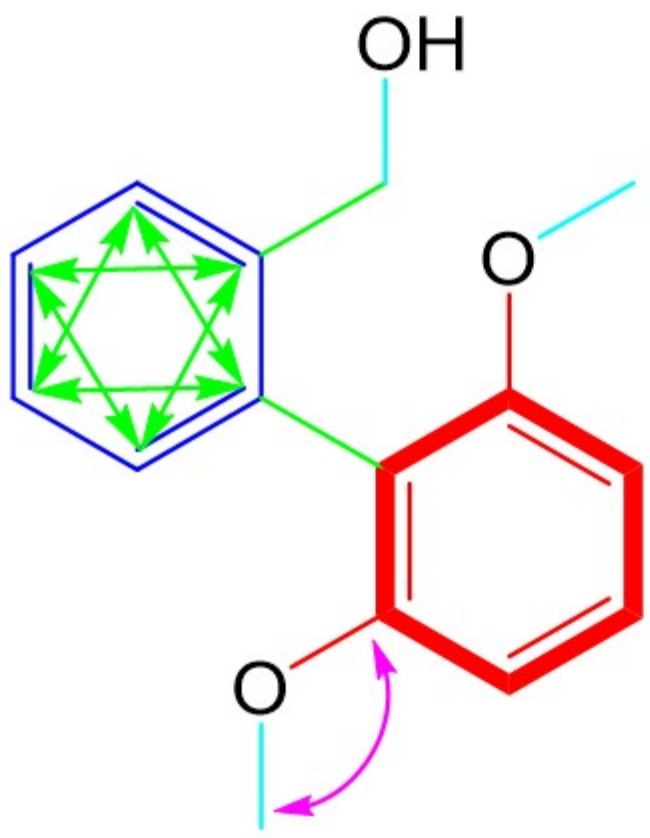


```
SADI C1A C2A C2A C3A C3A C4A C4A C5A C5A C6A C6A C1A
SADI C1A C3A C2A C4A C3A C5A C4A C6A C5A C1A C6A C2A
AFIX 66 C7A C8A C9A C10A C11A C12A
SADI C4A C7A C5A C13A
SADI O1A C13A O2A C14A O3A C15A
DFIX 1.37 O2A C8A O3A C12A
DANG 2.35 C8A C14A
SIMU C1A C2A C3A C4A C5A C6A C7A C8A C9A C10A C11A C12A =
 C13A C14A C15A O1A O2A O3A
RIGU C1A C2A C3A C4A C5A C6A C7A C8A C9A C10A C11A C12A =
 C13A C14A C15A O1A O2A O3A
```

Figure S 3: BBA-8,12-OMe restraints / constraints diagram and text-based equivalent.

1.3. Conventional SCXRD Data Collection

For crystals grown via the ENaCt method, or by slow evaporation, single crystal X-ray diffraction experiments were carried out on the following three instruments:

(1) Rigaku 007HF diffractometer using Cu-K_α radiation (λ = 1.54184 Å), equipped with Varimax confocal mirrors, UG2 goniometer, and HyPix 6000HE / HyPix Arc-100 detectors.

(2) Rigaku FRE+ diffractometer using Mo-K_α radiation (λ = 0.71075 Å), equipped with HF Varimax confocal mirrors, UG2 goniometer, and HyPix 6000HE detector.

(3) Rigaku XtaLAB Synergy-S diffractometer using Cu-K_α radiation (λ = 1.54184 Å), equipped with HyPix Arc-100 detector.

1.4. Crystalline Sponge Experimental Conditions

Table S 1: Crystalline sponge guest exchange conditions.

Analyte	Exchange Temperature / °C	Exchange Duration / hours	Analyte Solvent	Analyte Concentration / mg mL^{-1}	Analyte Volume / µL
BBA-1-Me	50	24	DCM	10	1
BBA	50	24	DCM	2	5
BBA-3-Me	50	24	DCM	10	1
BBA-(1-Me)-8,12-OMe	50	96	DCM	10	1
BBA-8,12-OMe	50	48	DCM	10	1
BBA-(3-Me)-8,12-OMe	50	96	DCM	10	1
BBA-1,8,12-OMe	50	96	DCM	10	1
BBA-8,10,12-OMe	50	24	DCM	10	1

The guest exchange procedure for each analyte was an iterative process, guided by previous knowledge of these analytes and a statistical optimisation investigation (Carroll and Coles, IUCrJ, 2024). The exchange conditions reported in the manuscript represent the best result achieved from the iterative process and comparison between datasets employed the quantitative CS grading system previously developed (Carrol and Coles, IUCrJ, 2024).

1.5. Conventional SCXRD Crystallisations

The following samples were crystallised via slow evaporation from concentrated (~20 mg mL^{-1}) DCM solutions: BBA, BBA-3-Me, BBA-(1-Me)-8,12-OMe, BBA-8,10,12-OMe.

All others were submitted for characterisation via ENaCt methodology, carried out as previously described in the literature with specific conditions described below.[5]

BBA-1-Me (brown powder, 20.9 mg, 80.9 μmol) was dissolved in 1.2 mL of methanol. This MeOH stock solution (17.4 mg mL^{-1}) was then transferred into 12 screw top vials (100 μL each). The solvent was allowed to evaporate in air at room temperature overnight. Each screw top vial contained approximately 1.74 mg of BBA-(1-Me)-8,12-Me. Collected Plate 3 H6, clear needle, nitromethane (@36.3 mg/mL, 50 nL) and FC-40 (200 nL).

BBA-(3-Me)-8,12-OMe (beige powder, 19.2 mg, 74.4 μmol) was dissolved in 1.2 mL of methanol. This MeOH stock solution (16 mg mL^{-1}) was then transferred into 12 screw top vials (100 μL each). The solvent was allowed to evaporate in air at room temperature overnight. Each screw top vial contained approximately 1.6 mg of BBA-(3-Me)-8,12-OMe. Collected Plate 2 Well A4, small clear block, toluene (@33.3mg/mL, 50 nL) and PDMSO (200 nL)

BBA-1,8,12-OMe (beige powder, 22.3 mg, 80.9 μmol) was dissolved in 1.2 mL of methanol. This MeOH stock solution (18.6 mg mL^{-1}) was then transferred into 12 screw top vials (100 μL each). The solvent was allowed to evaporate in air at room temperature overnight. Each screw top vial contained approximately 1.86 mg of BBA-1,8,12-OMe. Collected Plate 3 Well D9, small clear block, acetonitrile (@77.5 mg/mL, 50 nL) and mineral oil (200 nL).

BBA-8,12-OMe (pale off white powder, 27.7 mg, 113 μmol) was dissolved in 2 mL of methanol. This MeOH stock solution (13.9 mg mL^{-1}) was then transferred into 20 screw top vials (100 μL each). The solvent was allowed to evaporate in air at room temperature overnight. Each screw top vial contained approximately 1.39 mg of BBA-8,12-OMe. Collected Plate 1 Well G3, small needle, HFIP (28.9 mg mL^{-1}) and PDMSO (200 nL).

1.6. Crystallographic Tables, Diagrams, and Notes

Table S 2: BBA conventional SCXRD crystallographic data.

CCDC Deposition Number	2341223
Empirical formula	$C_{15}H_{16}O$
Formula weight	212.28
Temperature/K	100.00(10)
Crystal system	monoclinic
Space group	Ia
a/Å	7.3966(4)
b/Å	20.8923(10)
c/Å	15.2539(8)
α/°	90
β/°	94.347(5)
γ/°	90
Volume/Å^3	2350.4(2)
Z	8
ρ$_{calc}$g/cm^3	1.2
μ/mm^{-1}	0.566
F(000)	912
Crystal size/mm^3	0.26 × 0.03 × 0.02
Radiation	Cu Kα (λ = 1.54184)
2Θ range for data collection/°	7.188 to 136.496
Index ranges	-8 ≤ h ≤ 8, -25 ≤ k ≤ 25, -18 ≤ l ≤ 17
Reflections collected	9843
Independent reflections	3769 [R_{int} = 0.0431, R_{sigma} = 0.0533]
Data/restraints/parameters	3769/2/295
Goodness-of-fit on F^2	1.023
Final R indexes [I>=2σ (I)]	R_1 = 0.0411, wR_2 = 0.0977
Largest diff. peak/hole / e Å^{-3}	0.19/-0.21
Flack Parameter	-0.1(2)

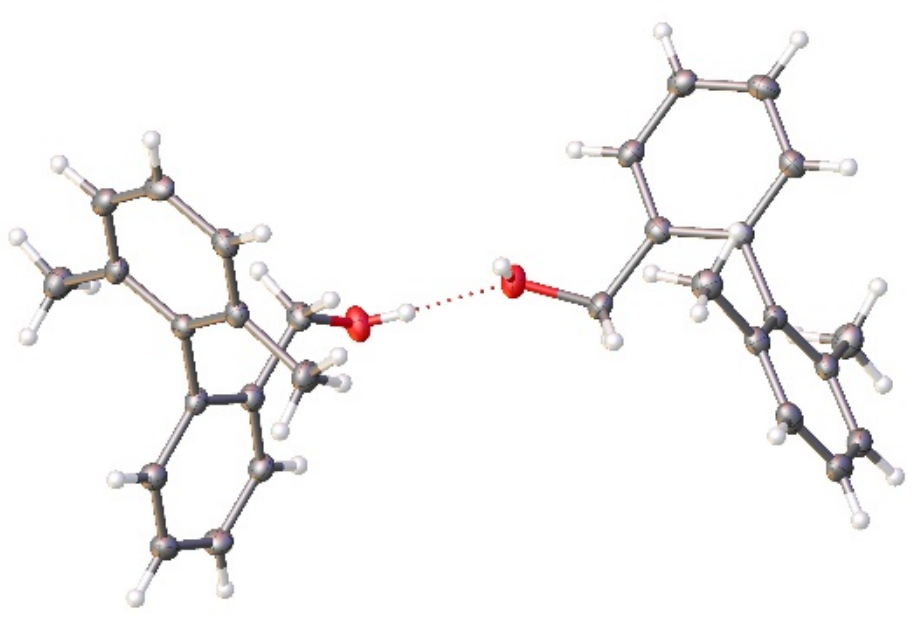

Asymmetric unit of BBA pure crystal with thermal ellipsoids displayed at 50% probability.

Table S 3: BBA-1-Me conventional SCXRD crystallographic data.

CCDC Deposition Number	2341234
Empirical formula	$C_{16}H_{18}O$
Formula weight	226.3
Temperature/K	150.15
Crystal system	orthorhombic
Space group	Iba2
a/Å	15.7149(4)
b/Å	21.2223(7)
c/Å	7.8588(2)
α/°	90
β/°	90
γ/°	90
Volume/Å^3	2620.96(13)
Z	8
$ρ_{calc}$g/cm^3	1.147
μ/mm^{-1}	0.535
F(000)	976
Crystal size/mm^3	0.544 × 0.034 × 0.031
Radiation	CuKα (λ = 1.54184)
2Θ range for data collection/°	7 to 155.2
Index ranges	-19 ≤ h ≤ 19, -26 ≤ k ≤ 25, -9 ≤ l ≤ 9
Reflections collected	19522
Independent reflections	2595 [R_{int} = 0.0302, R_{sigma} = 0.0261]
Data/restraints/parameters	2595/9/171
Goodness-of-fit on F^2	1.085
Final R indexes [I>=2σ (I)]	R_1 = 0.0357, wR_2 = 0.0935
Largest diff. peak/hole / e Å^{-3}	0.17/-0.14
Flack Parameter	-0.04(14)

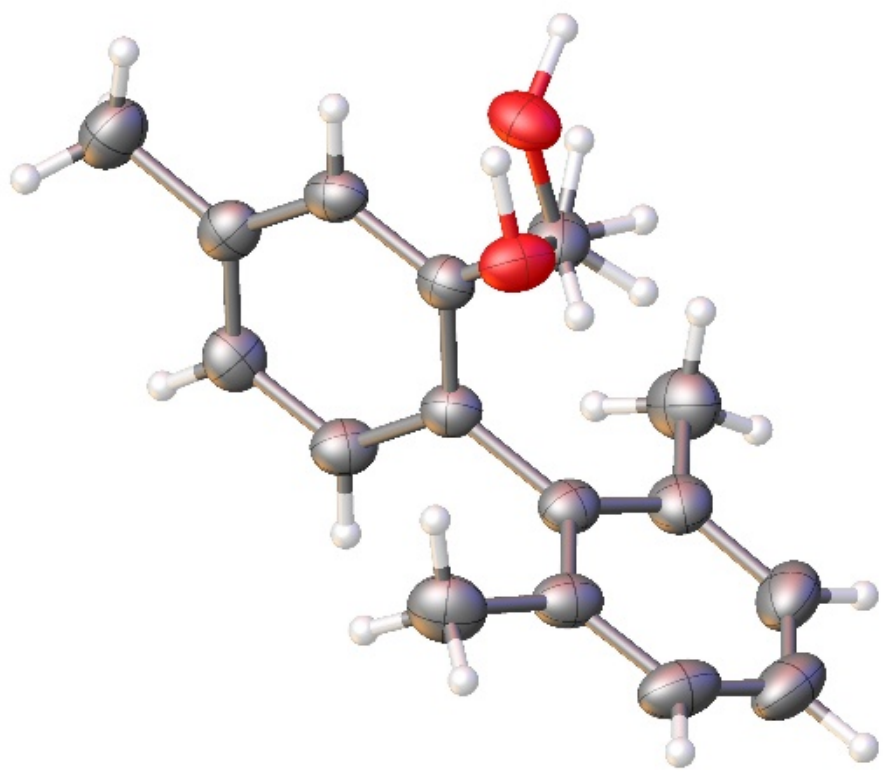

Asymmetric unit of BBA-1-Me pure crystal with thermal ellipsoids displayed at 50% probability.

Table S 4: BBA-3-Me conventional SCXRD crystallographic data.

CCDC Deposition Number	2341231
Empirical formula	$C_{16}H_{18}O$
Formula weight	226.321
Temperature/K	100(2)
Crystal system	triclinic
Space group	P-1
a/Å	7.8263(6)
b/Å	11.6614(10)
c/Å	14.9752(13)
α/°	101.698(7)
β/°	93.345(7)
γ/°	97.367(7)
Volume/Å^3	1322.3(2)
Z	2
$ρ_{calc}$g/cm^3	1.137
μ/mm^{-1}	0.531
F(000)	489.4
Crystal size/mm^3	0.24 × 0.14 × 0.06
Radiation	Cu Kα (λ = 1.54184)
2Θ range for data collection/°	6.04 to 150.3
Index ranges	-9 ≤ h ≤ 7, -14 ≤ k ≤ 14, -17 ≤ l ≤ 18
Reflections collected	22188
Independent reflections	5222 [R_{int} = 0.0490, R_{sigma} = 0.0360]
Data/restraints/parameters	5222/7/325
Goodness-of-fit on F^2	1.062
Final R indexes [I>=2σ (I)]	R_1 = 0.0783, wR_2 = 0.1880
Largest diff. peak/hole / e Å^{-3}	0.31/-0.30

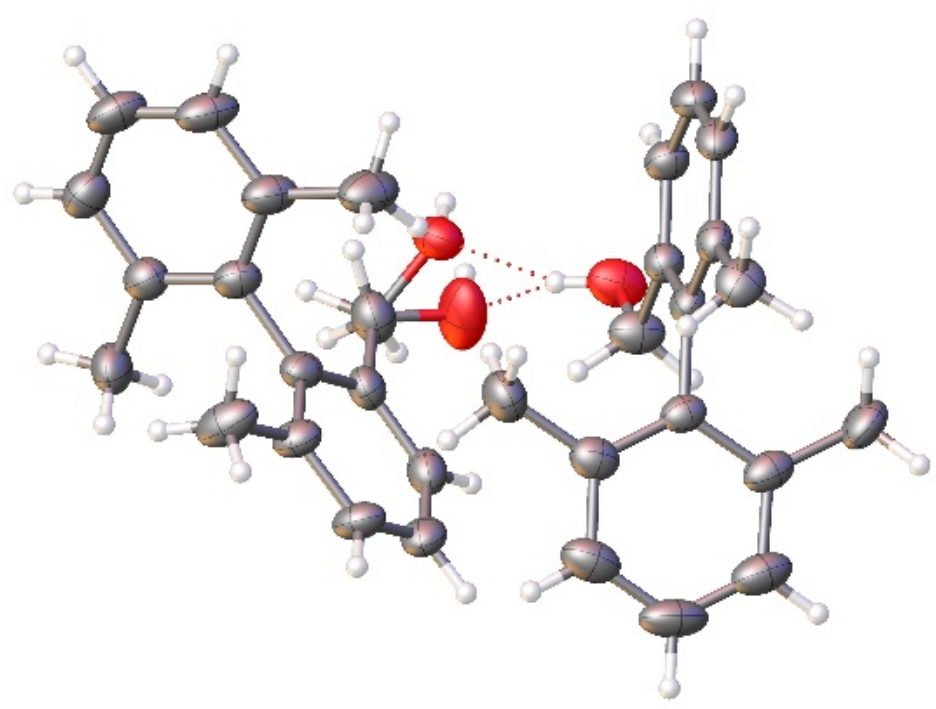

Asymmetric unit of BBA-3-Me pure crystal with thermal ellipsoids displayed at 50% probability.

Table S 5: BBA-(1-Me)-8,12-OMe conventional SCXRD crystallographic data.

CCDC Deposition Number	2341225
Empirical formula	$C_{16}H_{18}O_3$
Formula weight	258.3
Temperature/K	100.00(10)
Crystal system	triclinic
Space group	P-1
a/Å	7.7775(6)
b/Å	8.1586(6)
c/Å	10.8093(7)
α/°	76.513(6)
β/°	83.023(6)
γ/°	87.615(6)
Volume/Å^3	661.97(8)
Z	2
$ρ_{calc}$g/cm^3	1.296
μ/mm^{-1}	0.714
F(000)	276
Crystal size/mm^3	0.27 × 0.15 × 0.06
Radiation	Cu Kα (λ = 1.54184)
2Θ range for data collection/°	8.468 to 138.9
Index ranges	-9 ≤ h ≤ 9, -8 ≤ k ≤ 9, -12 ≤ l ≤ 13
Reflections collected	8282
Independent reflections	2449 [R_{int} = 0.0157, R_{sigma} = 0.0148]
Data/restraints/parameters	2449/0/176
Goodness-of-fit on F^2	1.052
Final R indexes [I>=2σ (I)]	R_1 = 0.0359, wR_2 = 0.1006
Largest diff. peak/hole / e Å^{-3}	0.25/-0.22

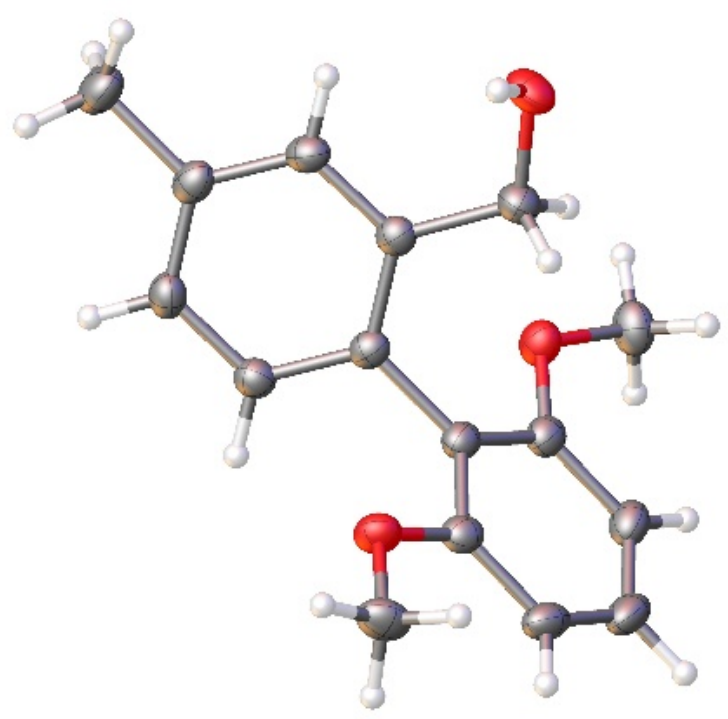

Asymmetric unit of BBA-(1-Me)-8,12-OMe pure crystal with thermal ellipsoids displayed at 50% probability.

Table S 6: BBA-(3-Me)-8,12-OMe conventional SCXRD crystallographic data.

CCDC Deposition Number	2341233
Empirical formula	$C_{16}H_{18}O_3$
Formula weight	258.3
Temperature/K	150.15
Crystal system	monoclinic
Space group	$P2_1/n$
a/Å	8.06454(8)
b/Å	13.38061(16)
c/Å	12.88656(12)
α/°	90
β/°	90.4137(10)
γ/°	90
Volume/Å^3	1390.53(3)
Z	4
$ρ_{calc}$g/cm^3	1.234
μ/mm^{-1}	0.68
F(000)	552
Crystal size/mm^3	0.255 × 0.107 × 0.05
Radiation	CuKα (λ = 1.54184)
2Θ range for data collection/°	9.528 to 152.996
Index ranges	-10 ≤ h ≤ 9, -16 ≤ k ≤ 16, -16 ≤ l ≤ 13
Reflections collected	20114
Independent reflections	2833 [R_{int} = 0.0247, R_{sigma} = 0.0157]
Data/restraints/parameters	2833/1/178
Goodness-of-fit on F^2	1.052
Final R indexes [I>=2σ (I)]	R_1 = 0.0422, wR_2 = 0.1200
Largest diff. peak/hole / e Å^{-3}	0.26/-0.22

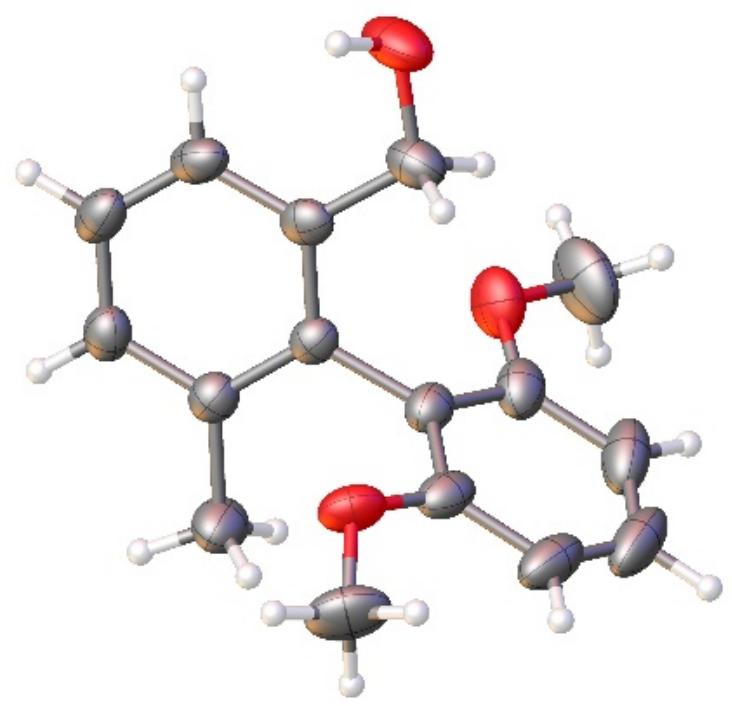

Asymmetric unit of BBA-(3-Me)-8,12-OMe pure crystal with thermal ellipsoids displayed at 50% probability.

Table S 7: BBA-8,10,12-OMe conventional SCXRD crystallographic data.

CCDC Deposition Number	2341232
Empirical formula	$C_{16}H_{18}O_4$
Formula weight	274.3
Temperature/K	100(2)
Crystal system	monoclinic
Space group	$P2_1/n$
a/Å	7.4432(4)
b/Å	14.3281(7)
c/Å	13.2190(10)
α/°	90
β/°	102.418(6)
γ/°	90
Volume/Å^3	1376.78(15)
Z	4
$ρ_{calc}$g/cm^3	1.323
μ/mm^{-1}	0.094
F(000)	584
Crystal size/mm^3	0.11 × 0.07 × 0.03
Radiation	Mo Kα (λ = 0.71073)
2Θ range for data collection/°	4.246 to 61.014
Index ranges	-10 ≤ h ≤ 10, -20 ≤ k ≤ 20, -18 ≤ l ≤ 18
Reflections collected	29796
Independent reflections	4205 [R_{int} = 0.0513, R_{sigma} = 0.0366]
Data/restraints/parameters	4205/0/253
Goodness-of-fit on F^2	1.023
Final R indexes [I>=2σ (I)]	R_1 = 0.0495, wR_2 = 0.1191
Largest diff. peak/hole / e Å^{-3}	0.42/-0.20

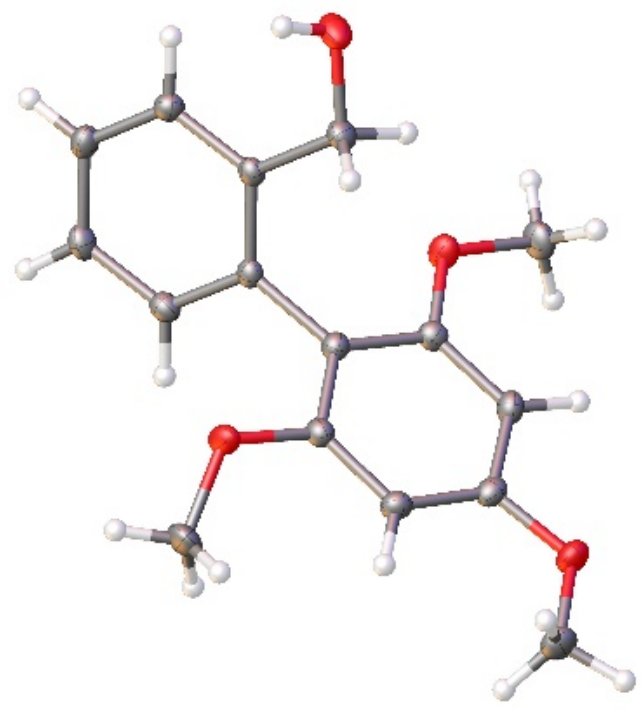

Asymmetric unit of BBA-8,10,12-OMe pure crystal with thermal ellipsoids displayed at 50% probability.

Table S 8: BBA-1,8,12-OMe conventional SCXRD crystallographic data.

CCDC Deposition Number	2341224
Empirical formula	$C_{16}H_{18}O_4$
Formula weight	274.3
Temperature/K	150.15
Crystal system	triclinic
Space group	P-1
a/Å	8.2956(2)
b/Å	10.4717(2)
c/Å	16.4353(3)
α /°	86.722(2)
β/°	76.201(2)
γ /°	77.806(2)
Volume/Å^3	1355.20(5)
Z	2
$ρ_{calc}$g/cm^3	1.344
μ/mm^{-1}	0.787
F(000)	584
Crystal size/mm^3	0.155 × 0.075 × 0.049
Radiation	CuKα (λ = 1.54184)
2Θ range for data collection/°	5.538 to 155.552
Index ranges	-10 ≤ h ≤ 10, -12 ≤ k ≤ 13, -20 ≤ l ≤ 20
Reflections collected	40808
Independent reflections	5448 [R_{int} = 0.0242, R_{sigma} = 0.0148]
Data/restraints/parameters	5448/0/373
Goodness-of-fit on F^2	1.027
Final R indexes [I>=2σ (I)]	R_1 = 0.0342, wR_2 = 0.0882
Largest diff. peak/hole / e Å^{-3}	0.24/-0.20

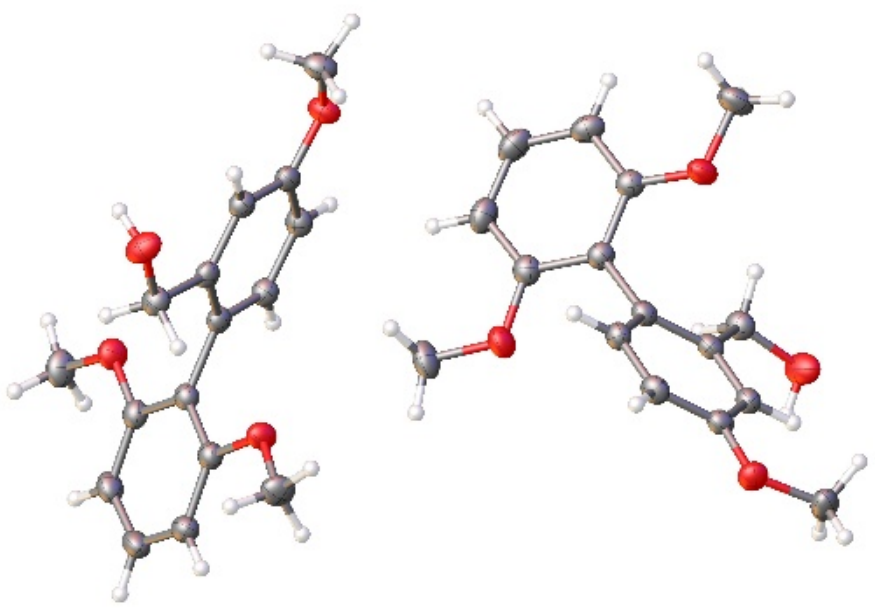

Asymmetric unit of BBA-1,8,12-OMe pure crystal with thermal ellipsoids displayed at 50% probability.

Table S 9: BBA-1-Me crystalline sponge crystallographic data.

CCDC Deposition Number	2359637
Empirical formula	$C_{107.68}H_{95.55}I_{12}N_{24}O_{1.7}Zn_6$
Formula weight	3667.93
Temperature/K	100.00(10)
Crystal system	triclinic
Space group	P-1
a/Å	14.8789(8)
b/Å	19.1162(9)
c/Å	30.3811(10)
α/°	99.970(3)
β/°	91.188(4)
γ/°	111.784(5)
Volume/Å³	7868.8(7)
Z	2
ρ_{calc}g/cm³	1.548
μ/mm⁻¹	19.839
F(000)	3478
Crystal size/mm³	0.22 × 0.14 × 0.07
Radiation	Cu Kα (λ = 1.54184)
2θ range for data collection/°	5.076 to 137.396
Index ranges	-16 ≤ h ≤ 17, -22 ≤ k ≤ 23, -36 ≤ l ≤ 36
Reflections collected	41668
Independent reflections	41668 [R_{int} = 0.3665, R_{sigma} = 0.0189]
Data/restraints/parameters	41668/894/1774
Goodness-of-fit on F²	1.012
Final R indexes [I>=2σ (I)]	R_1 = 0.0901, wR_2 = 0.2659
Largest diff. peak/hole / e Å⁻³	1.50/-1.82

SQUEEZE analysis indicated two voids with volumes of 168 and 151 and electron counts of 50 and 20 respectively. This is equivalent to 1.04 and 0.42 cyclohexane molecules, or 0.42 and 0.17 BBA-1-Me molecules

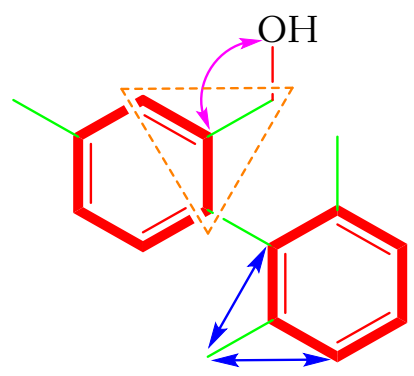

60.1%

DANG [C5A – O1A]

SIMU

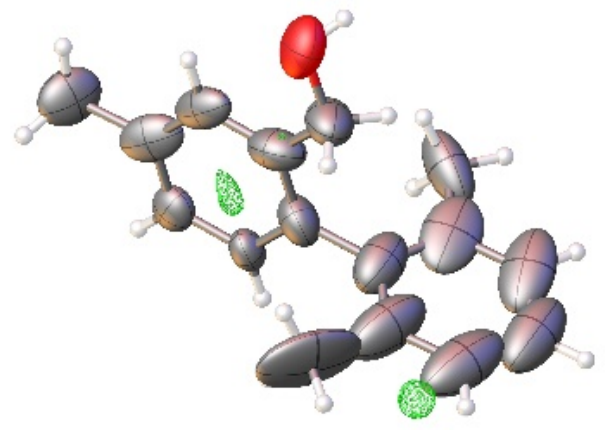

Geometric restraints and thermal ellipsoid (50% probability) for BBA-1-Me Site A

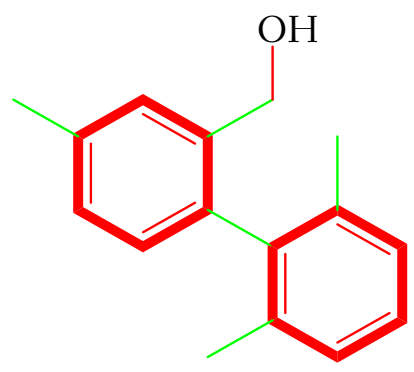

59.4%

SIMU

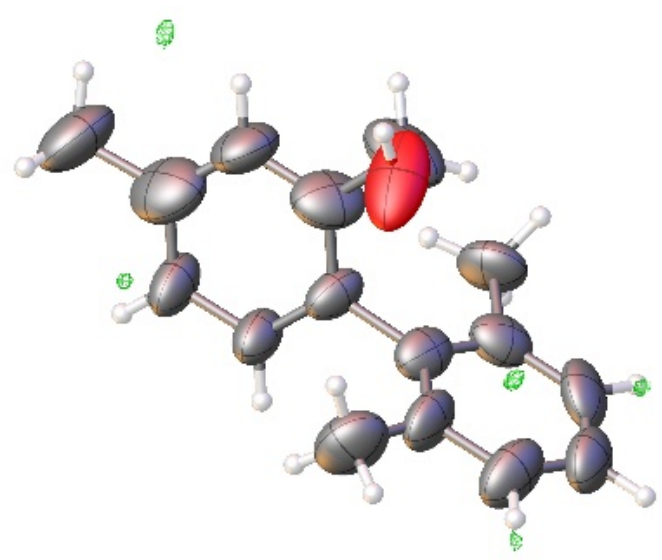

Geometric restraints and thermal ellipsoid (50% probability) for BBA-1-Me Site B

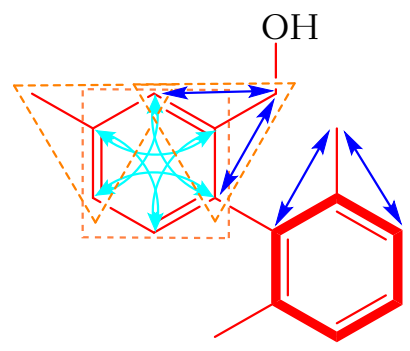

50.0%

SIMU

RIGU

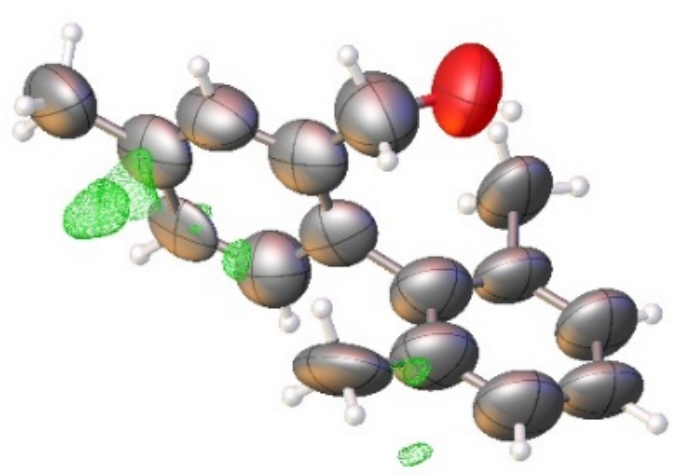

Geometric restraints and thermal ellipsoid (50% probability) for BBA-1-Me Site C

Table S 10: BBA-(1-Me)-8,12-OMe crystalline sponge crystallographic data.

CCDC Deposition Number	2359638
Empirical formula	$C_{50.41}H_{41.1}I_6N_{12}O_{2.51}Zn_3$
Formula weight	1812.58
Temperature/K	100.00(10)
Crystal system	monoclinic
Space group	C2/c
a/Å	34.3966(12)
b/Å	14.9997(4)
c/Å	31.701(2)
α/°	90
β/°	101.865(5)
γ/°	90
Volume/Å³	16006.1(14)
Z	8
$□_{calc}$g/cm³	1.504
μ/mm⁻¹	19.516
F(000)	6845
Crystal size/mm³	0.17 × 0.16 × 0.1
Radiation	Cu Kα (λ = 1.54184)
2Θ range for data collection/°	5.25 to 136.496
Index ranges	-28 ≤ h ≤ 41, -18 ≤ k ≤ 16, -38 ≤ l ≤ 37
Reflections collected	59435
Independent reflections	14515 [R_{int} = 0.0348, R_{sigma} = 0.0263]
Data/restraints/parameters	14515/1058/1104
Goodness-of-fit on F²	1.071
Final R indexes [I>=2σ (I)]	R_1 = 0.1124, wR_2 = 0.3185
Largest diff. peak/hole / e Å⁻³	0.90/-1.11

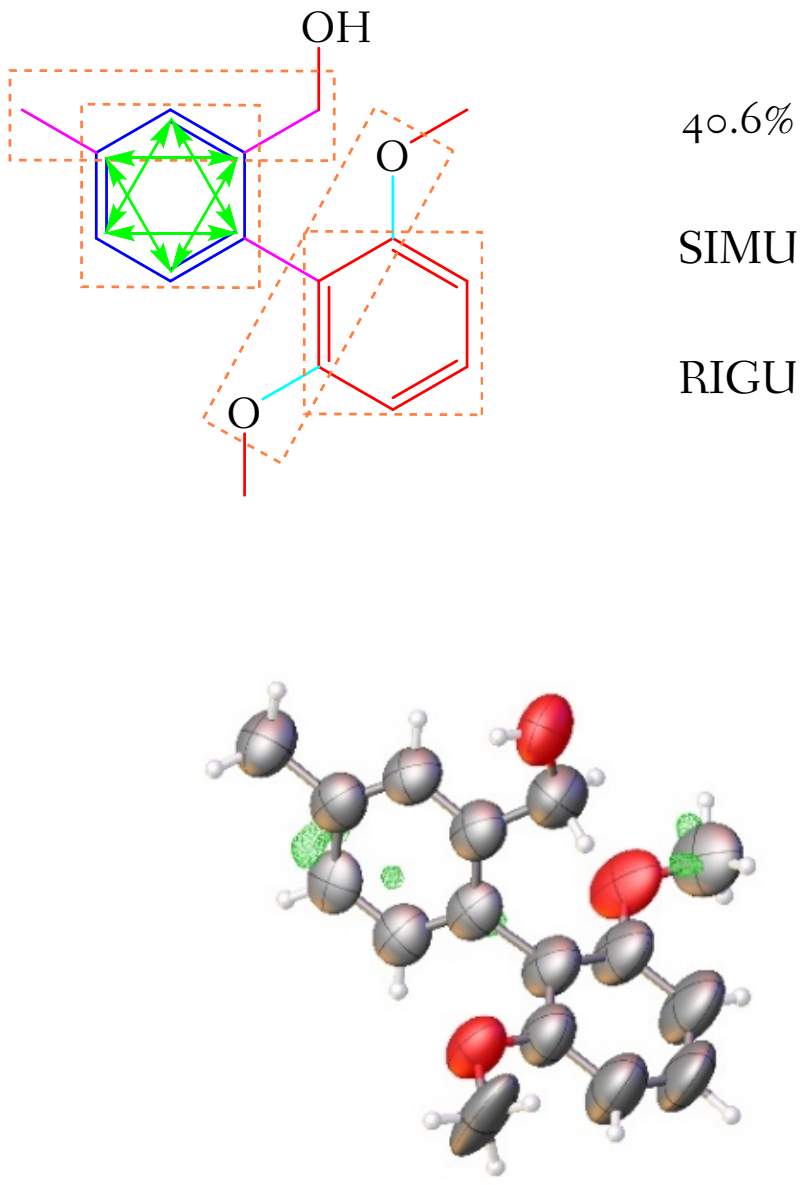

Geometric restraints and thermal ellipsoid (50% probability) for BBA-(1-Me)-8,12-OMe Site A

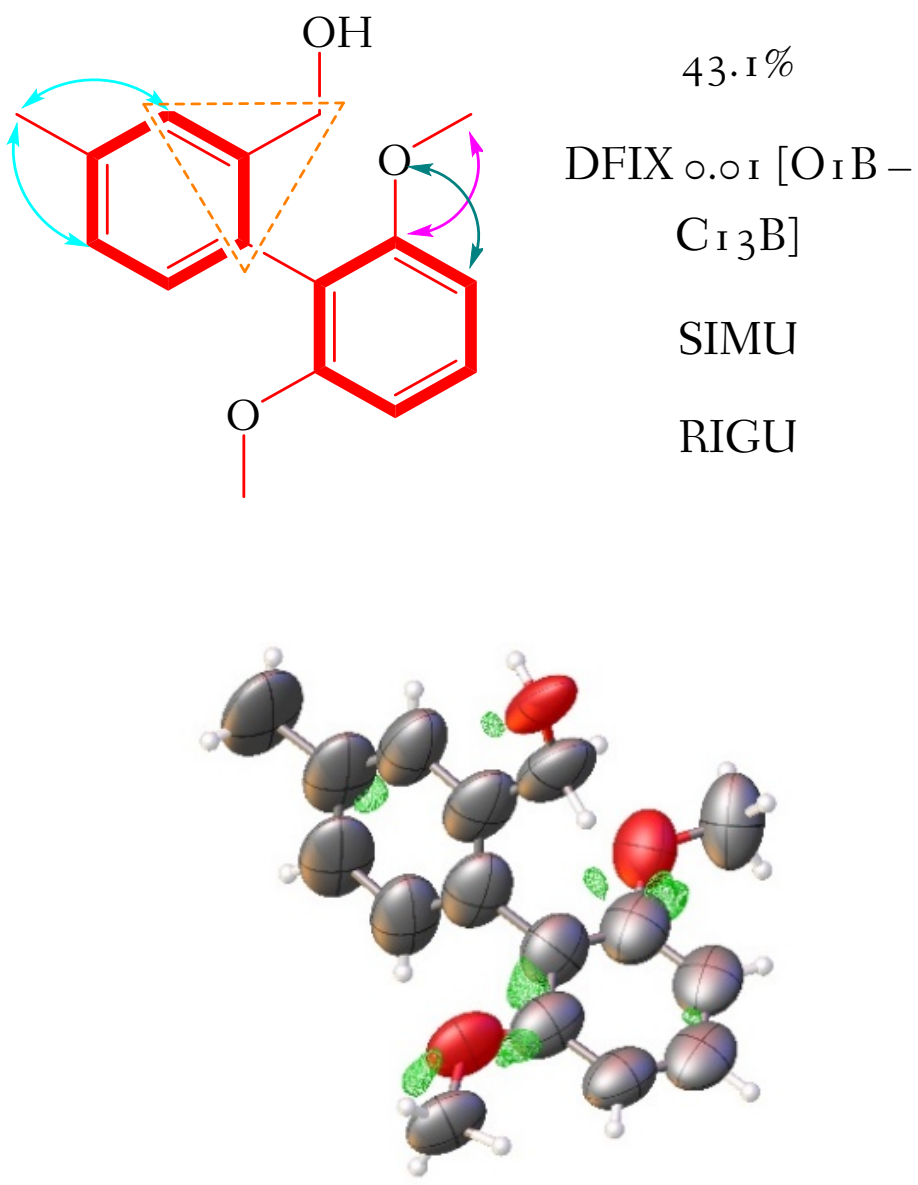

Geometric restraints and thermal ellipsoid (50% probability) for BBA-(1-Me)-8,12-OMe Site B

Table S 11: BBA-(3-Me)-8,12-OMe crystalline sponge crystallographic data.

CCDC Deposition Number	2359639
Empirical formula	$C_{38.36}H_{27.18}I_6N_{12}O_{0.33}Zn_3$
Formula weight	1619.03
Temperature/K	100(2)
Crystal system	monoclinic
Space group	C2/c
a/Å	34.8186(7)
b/Å	14.9253(2)
c/Å	31.1729(7)
α/°	90
β/°	102.160(2)
γ/°	90
Volume/Å³	15836.4(5)
Z	8
$□_{calc}$g/cm³	1.358
μ/mm⁻¹	19.636
F(000)	6016
Crystal size/mm³	0.15 × 0.12 × 0.05
Radiation	Cu Kα (λ = 1.54184)
2Θ range for data collection/°	5.192 to 136.492
Index ranges	-36 ≤ h ≤ 41, -17 ≤ k ≤ 12, -37 ≤ l ≤ 37
Reflections collected	52880
Independent reflections	14371 [R_{int} = 0.0293, R_{sigma} = 0.0266]
Data/restraints/parameters	14371/407/821
Goodness-of-fit on F²	1.067
Final R indexes [I>=2σ (I)]	R_1 = 0.0735, wR_2 = 0.2307
Largest diff. peak/hole / e Å⁻³	1.00/-1.00

SQUEEZE analysis indicated one voids with a volume of 552 and electron count of 133. This is equivalent to 2.77 cyclohexane molecules, or 0.0.96 BBA-(3-Me)-8,12-OMe molecules.

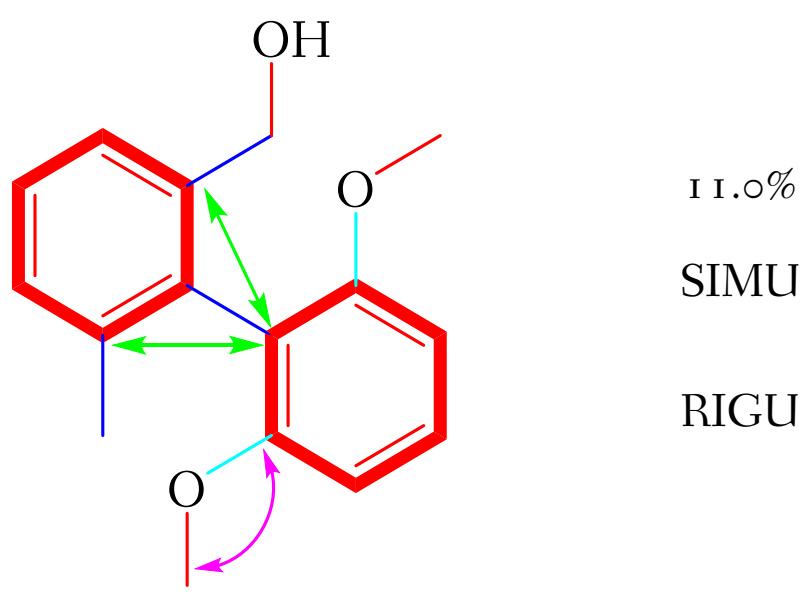

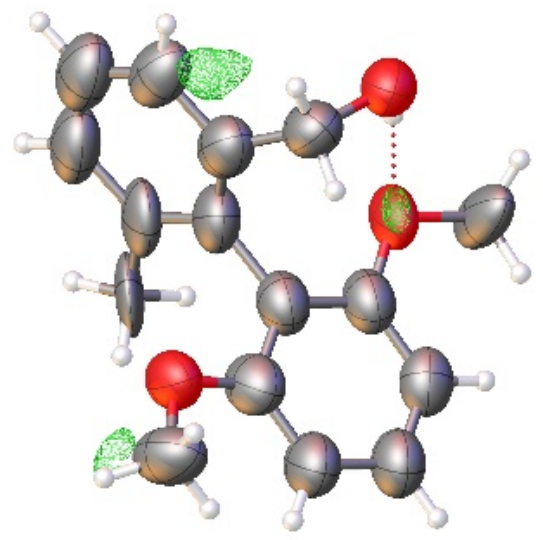

Geometric restraints and thermal ellipsoid (50% probability) for BBA-(3-Me)-8,12-OMe Site A

Table S 12: BBA-1,8,12-OMe crystalline sponge crystallographic data.

CCDC Deposition Number	2359640
Empirical formula	$C_{46.79}H_{37.58}I_6N_{12}O_{2.29}Zn_3$
Formula weight	1762.09
Temperature/K	100.00(10)
Crystal system	monoclinic
Space group	C2/c
a/Å	34.6263(10)
b/Å	14.9166(3)
c/Å	31.5522(8)
α/°	90
β/°	101.904(2)
γ/°	90
Volume/Å³	15946.4(7)
Z	8
□$_{calc}$g/cm³	1.468
μ/mm^{-1}	19.569
F(000)	6629
Crystal size/mm³	0.23 × 0.11 × 0.08
Radiation	Cu Kα (λ = 1.54184)
2Θ range for data collection/°	5.216 to 136.49
Index ranges	-41 ≤ h ≤ 41, -17 ≤ k ≤ 17, -38 ≤ l ≤ 32
Reflections collected	113488
Independent reflections	14588 [R_{int} = 0.0718, R_{sigma} = 0.0328]
Data/restraints/parameters	14588/755/1016
Goodness-of-fit on F²	1.065
Final R indexes [I>=2σ (I)]	R_1 = 0.1476, wR_2 = 0.3710
Largest diff. peak/hole / e Å^{-3}	0.96/-1.01

.

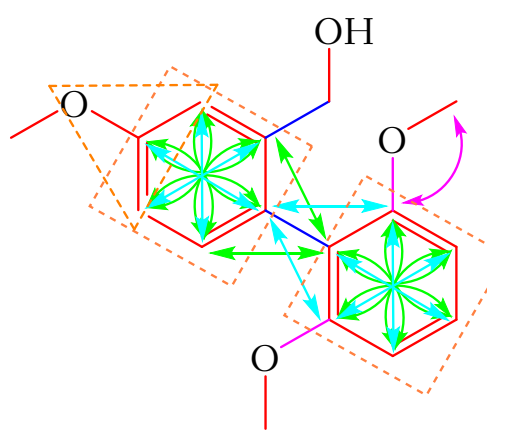

29.6%

SIMU

RIGU

DFIX 0.01 [C1A – O4A]

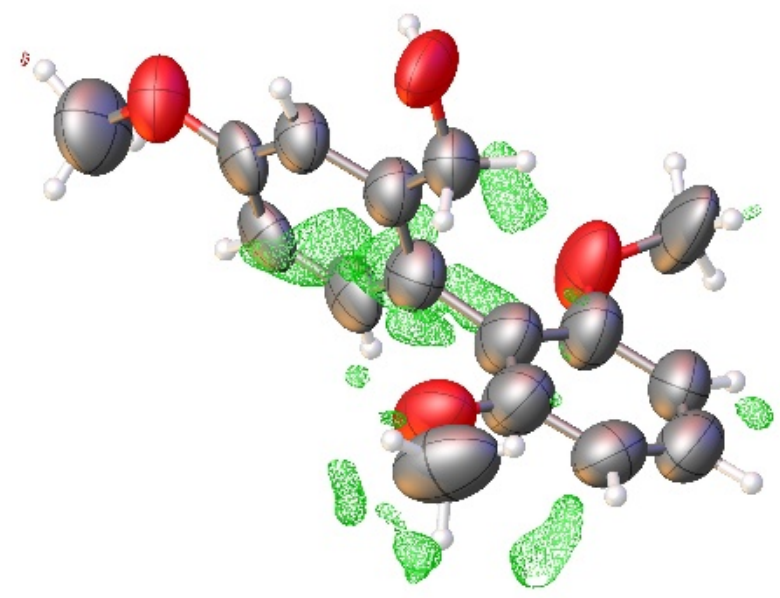

Geometric restraints and thermal ellipsoid (50% probability) for BBA-1,8,12-OMe Site A

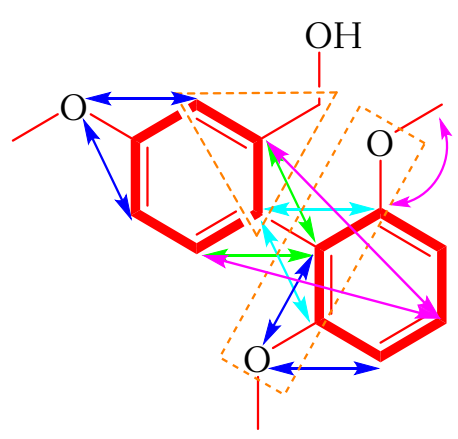

27.5%

SIMU

RIGU

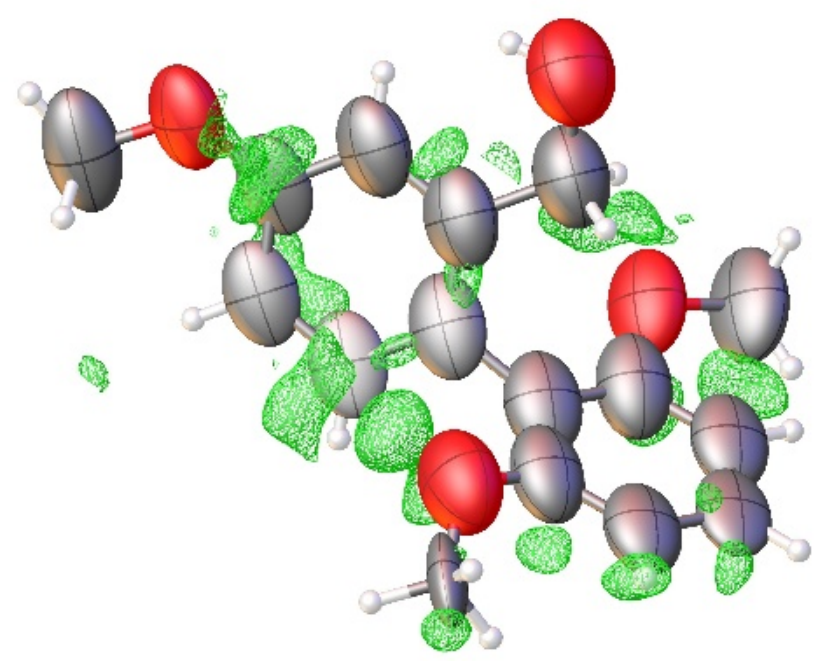

Geometric restraints and thermal ellipsoid (50% probability) for BBA-1,8,12-OMe Site B

2. Calculation of Intramolecular Energetics

2.1. Methods

All information on the preparation of the experimental datasets for computational study including notes on interpreting refinement statistics, and the computational methods for single-molecule and crystal geometry optimisations, conformational searches, and assessing the stability of conformers, can be found in the Supporting Information for the paper by Soper et. al *CrystEngComm*, 2025, https://doi.org/10.1039/D4CE00699B .

2.2. Definitions

$\square E_{strain}$ is an energetic measure of the distortion of the molecule away from its nearest stable gas phase geometry, and is calculated from the energetic difference between the crystalline molecular geometry ($E^{mol}_{crystal}$) and its local energy minimum conformer ($E^{opt,mol}_{local}$), obtained by unconstrained optimisation of the isolated experimental molecule (Equation S1):

$$\square E_{strain} = E^{mol}_{crystal} - E^{opt.\ mol}_{local} \tag{S1}$$

$\square E_{conf}$ describes the energetic difference between the global minimum conformer (E_{GM}) and the local energy minimum conformer corresponding to the experimental molecular geometry ($E^{opt,mol}_{local}$) (Equation S2):

$$\square E_{conf} = E^{opt.\ mol}_{local} - E_{GM} \tag{S2}$$

The total relative energy of the isolated molecule from the experimental crystal structure, $\square E^{intra}_{total}$, represents the energy difference between the molecular conformation in the optimised experimental structures ($E^{mol}_{crystal}$) and the most stable gas phase molecular geometry (E_{GM}), and can be calculated as the sum of $\square E_{strain}$ and $\square E_{conf}$ (Equation S3):

$$\square E^{intra}_{total} = \square E_{strain} + \square E_{conf} = E^{mol}_{crystal} - E_{GM} \tag{S3}$$

3. PIXEL Calculations

3.1. Workflow

To address the issue of compatibility of the PIXEL method with extended framework systems, the workflow illustrated in Figure S 4 was adopted for calculation of intermolecular interaction energies in CS structures.

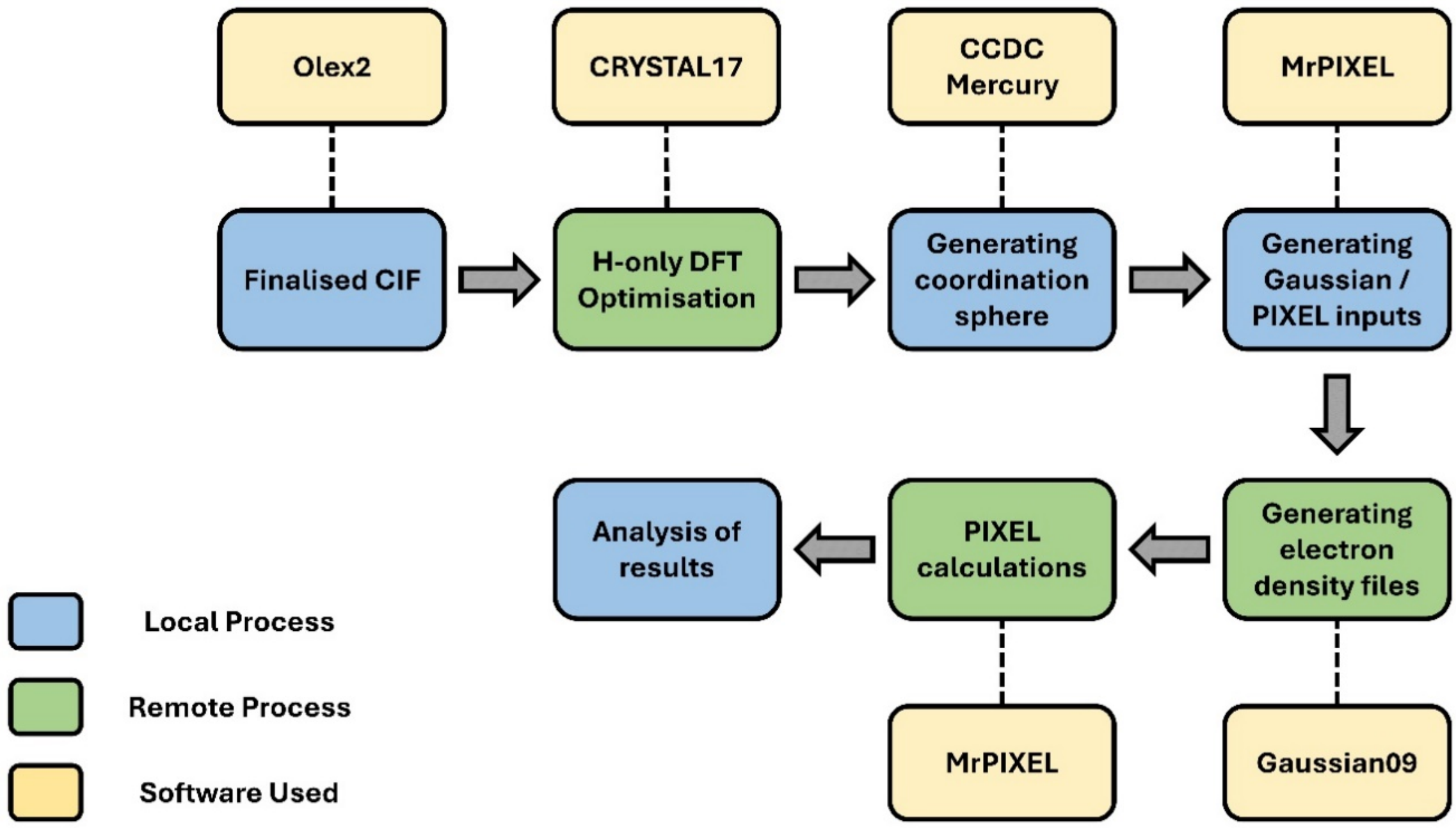

Figure S 4: Representative workflow for PIXEL intermolecular energy calculations of CS guests.

The process is notably more manual than the intended use of MrPIXEL for molecular organic crystal structures but similarly begins with a finalised CIF from CS data collection. This is followed by a constrained optimisation, relaxing only the hydrogen atoms of the prepared crystallographic model, to ensure hydrogen atoms are modelled in energetically sensible positions before intermolecular interaction calculations. The constrained geometry optimisation calculations were carried out using the same methods as for the relaxed geometry optimisation of crystal structures for calculation of intramolecular energetics in ESI Section 2, with the addition of the keywords ATOMONLY and FRAGMENT in the geometry section of the input file to define the hydrogen atoms. This is necessitated by the difficulty of reliably identifying hydrogen atoms in CS structures which can have significant impacts on calculations due to the exponential nature of interaction energies with respect to distance. The optimised structures are visualised in CCDC Mercury software, where unique guest exchange sites are isolated into individual CIFs before generation of coordination spheres to define local environments.[6] This process involves manual editing of larger framework fragments into consistent sizes and the rationale behind this is discussed in detail in ESI Section 3.2.

Following preparation of individual CIFs for unique exchange sites, structures are submitted to the MrPIXEL software which is integrated into CCDC Mercury via Python API.[7] The software generates Gaussian 09 job files and PIXEL input files for all possible pairwise intermolecular interactions within the coordination sphere.[8] Upon completion of file generation, manual checking is required to identify the relevant files for the central guest molecule and this also enables editing of any file format errors.

Submission of files to Gaussian 09 software is conducted on the University of Southampton IRIDIS high-performance computing facility to decrease the time taken for generation of density files. These files are used

in combination with the previously generated PIXEL input files for calculation of intermolecular interaction energies with MrPIXEL executable programmes, this is also conducted on the IRIDIS servers to increase the number of calculations which can be run simultaneously. Finally, users obtain intermolecular interaction energies which can then be related to structural motifs or molecular geometries.

3.2. Defining Coordination Spheres and Framework Fragments

As previously stated, MrPIXEL software is not directly compatible with extended framework systems. To address this issue, chemically reasonable fragments must be generated around a central guest molecule for the dimer energy calculations to be performed.[9,10]

This most commonly involves the addition of hydrogen atoms to terminate otherwise uncompleted chemical fragments. The implementation of PIXEL into CCDC's Mercury software via the MrPIXEL interface enables editing of CIFs graphically.[6] Although the generation of framework fragments and investigation of intermolecular interaction energies has previously been accomplished for a selection of framework materials, the ZnI_2 – tpt CS host presents an alternative challenge.[9,10]

The variability of guest exchange locations and changes to ligand geometry upon guest encapsulation prevent the use of consistent framework fragments for all samples. Consequently, each CS structure, and unique guest exchange site within, must have a bespoke set of nearby guest and framework molecules defined. This is considerably more time consuming than the standard implementation of PIXEL calculations for discrete molecular organic systems, and requires consideration of fragment sizes, or more broadly the radius of the coordination sphere from the central guest molecule.

The previously conducted investigation with PIXEL for framework systems and gas adsorbates maintained a consistent framework unit and varied the location of guest molecules.[10] Therefore, benchmarking studies to determine a coordination sphere radius which accounts for the majority of intermolecular interactions have not been completed for the new type of study proposed here. To identify suitable framework fragment sizes, intermolecular interaction energies were calculated for a range of coordination sphere sizes (3.0 – 5.0 Å). These studies also varied completeness of ligand molecules as indicated by the '+' suffix, i.e., whether the tpt ligand would be fully or partially modelled if most of the molecule was beyond the chosen coordination sphere radius. An example fragment is shown in Figure S 5 to illustrate the variation of molecule size.

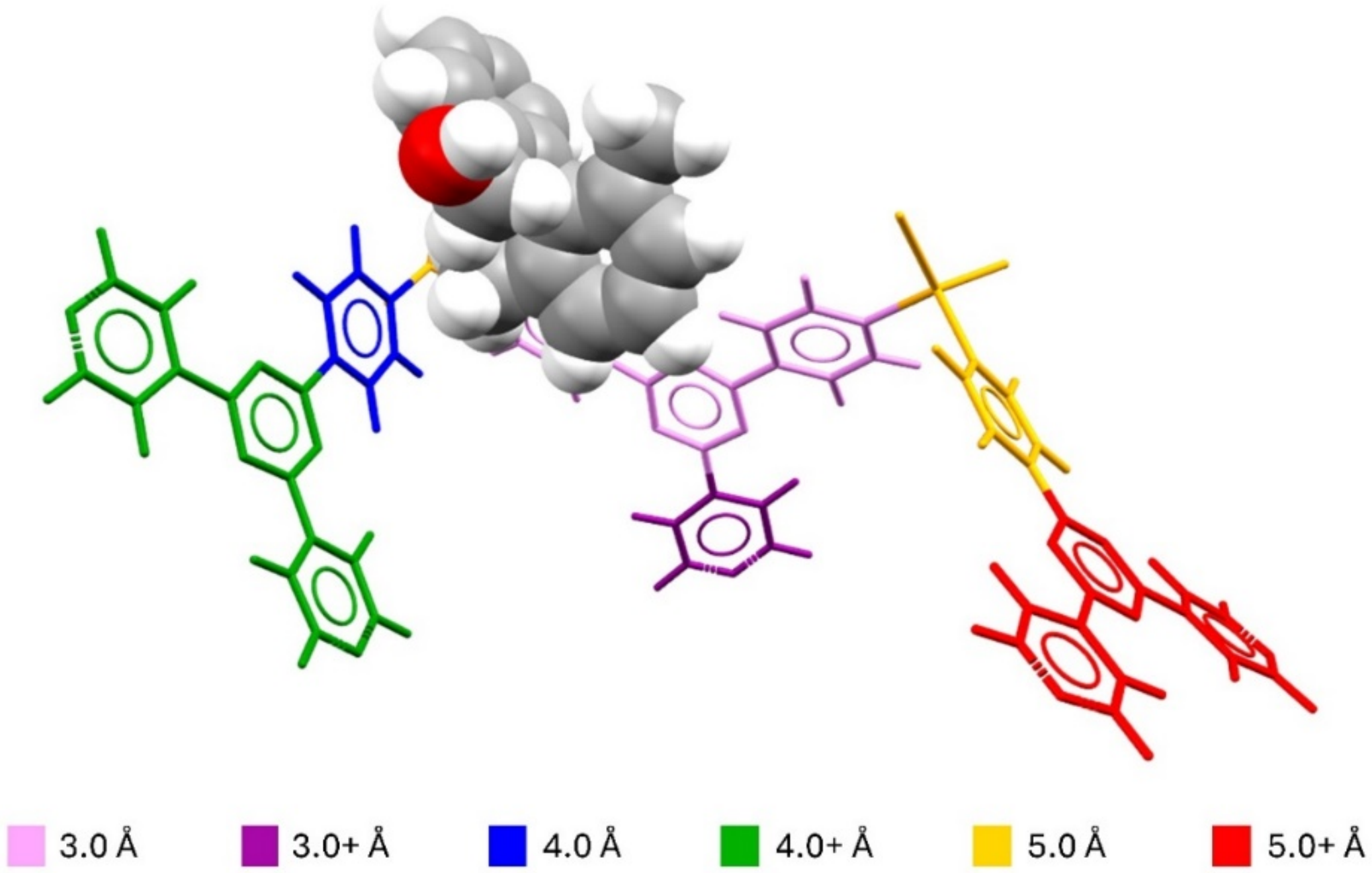

Figure S 5: Consistent central guest (BBA CS_A) with systematic expansion of coordination sphere and fragment size.

Comparative studies were conducted on BBA CS_A which is an ideal candidate because of its mixed host-guest and guest-guest interaction behaviour. To assess which coordination sphere radius and fragment sizes accurately account for most intermolecular interactions, interaction energy components were plotted as a function of coordination sphere radius, as displayed in Figure S 6.

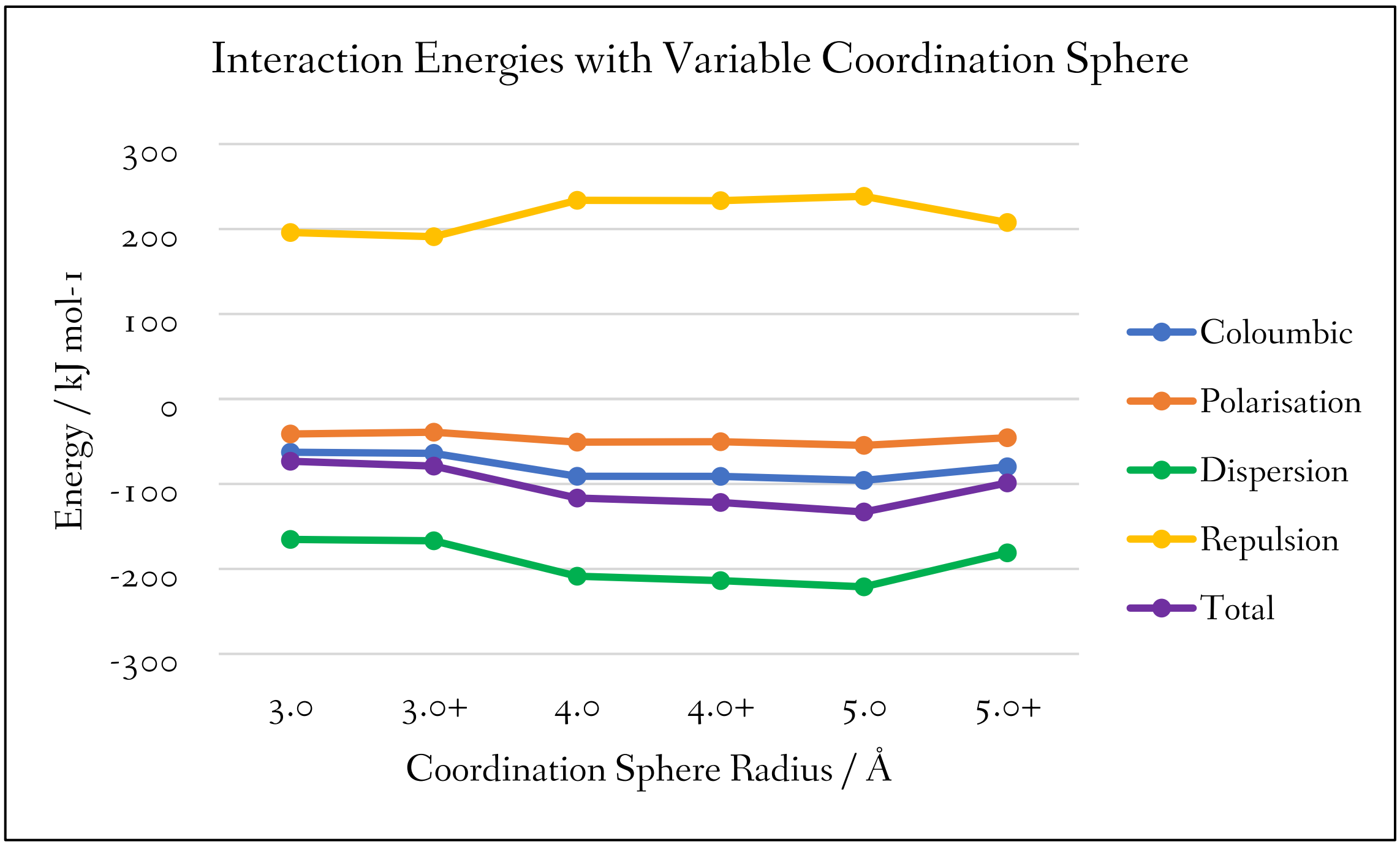

Figure S 6: Interaction energy components as a function of coordination sphere radius.

The relative plateau in all energetic components and total interaction energy (E_{TOT}) indicates that modelling framework fragments within a 4.0 Å radius, with shortened ligand components, represents the best compromise between accuracy of interaction energies balanced against computational resource requirement. The 4.0 Å coordination sphere radius has been applied to all pore environments modelled for this study, however it is suggested that new applications should conduct similar benchmarking studies to assess suitable ranges.

The changes in energy observed at 5.0+ Å in Figure S 6 are due to missing contributions from one framework fragment. The large size of the fragment ($C_{54}H_{36}N_{18}Zn_2I_4$) and the small density steps required for accurate modelling of iodine electron density led to issues with computer memory capacity during generation of density files in Gaussian 09 software. Attempts to run calculations on servers with larger memory capacities have not yet proved successful. Nevertheless, we believe that the larger fragment will not significantly impact the interaction environment. This insight has been informed by the trends observed up to the 5.0 Å radius, in combination with more distant fragments exhibiting weaker influence, as highlighted in Table S 13.

3.3. Coordination Sphere Benchmarking Tabulation

Table S 13: BBA CS_A MrPIXEL coordination sphere benchmarking results.

Coordination Sphere Radius	Fragment Formula	E_{COU}	E_{POL}	E_{DISP}	E_{REP}	E_{TOT}
3.0	C13 H9 N5	-6.8	-8.0	-32.6	39.5	-7.9
	C10 H10 N2 Zn I2	-26.2	-15.2	-33.3	52.7	-22.0
	C13 H9 N5	-8.9	-4.9	-24.8	24	-14.6
	C15 H16 O	-2.2	-2.2	-15.6	11.1	-8.9
	C15 H16 O	-4.0	-4.8	-24.2	28.7	-4.3
	C15 H16 O	-10.1	-5.3	-18.5	25.8	-8.1
	C15 H16 O	-3.6	-1.3	-16.5	10.7	-10.7
3.0+	C18 H12 N6	-8.1	-6.1	-32.2	34.6	-11.8
	C36 H24 N12 Zn I2	-26.2	-15	-35.3	52.7	-23.8
	C18 H12 N6	-9.8	-4.4	-24.6	22	-16.8
	C15 H16 O	-2.2	-2.2	-15.6	11.1	-8.9
	C15 H16 O	-4.0	-4.8	-24.2	28.7	-4.3
	C15 H16 O	-10.1	-5.3	-18.5	25.8	-8.1
	C15 H16 O	-3.6	-1.3	-16.5	10.7	-10.7
4.0	C18 H14 N6 Zn I2	-18.3	-9.4	-47.6	44.4	-30.9
	C10 H10 N2 Zn I2	-26.2	-15.2	-33.3	52.7	-22.0
	C13 H9 N5	-8.9	-4.9	-24.8	24	-14.6
	C5 H5 N	-1.6	-0.6	-7.2	4.1	-5.3
	C15 H16 O	-2.2	-2.2	-15.6	11.1	-8.9
	C15 H16 O	-4.0	-4.8	-24.2	28.7	-4.3
	C15 H16 O	-10.1	-5.3	-18.5	25.8	-8.1
	C10 H10 N2 Zn I2	-15.2	-7.6	-21.1	28.9	-15
	C15 H16 O	-3.6	-1.3	-16.5	10.7	-10.7
4.0+	C36 H24 N12 Zn I2	-18.7	-9.6	-49	44.5	-32.8
	C36 H24 N12 Zn I2	-26.2	-15	-35.3	52.7	-23.8
	C18 H12 N6	-9.8	-4.4	-24.6	22	-16.8
	C18 H12 N6	-1.5	-0.4	-8.7	3.7	-6.9
	C15 H16 O	-2.2	-2.2	-15.6	11.1	-8.9
	C15 H16 O	-4.0	-4.8	-24.2	28.7	-4.3
	C15 H16 O	-10.1	-5.3	-18.5	25.8	-8.1

Table S 16 (cont.)

4.0+	C36 H24 N12 Zn I2	-15	-7.3	-21.4	28.8	-14.9
	C15 H16 O	-3.6	-1.3	-16.5	10.7	-10.7
5.0	C21 N10 H15 Zn I2	-18.4	-9.5	-48.4	44.5	-31.8
	C13 H11 N5 Zn I2	-27.1	-15.2	-34.7	53	-24
	C26 H20 N10 Zn2 I4	-15.2	-8.6	-41.5	30.4	-34.9
	C15 H16 O	-2.2	-2.2	-15.6	11.1	-8.9
	C15 H16 O	-4.0	-4.8	-24.2	28.7	-4.3
	C15 H16 O	-10.1	-5.3	-18.5	25.8	-8.1
	C15 H16 O	0	-0.1	-0.6	0	-0.7
	C10 H8 N2 Zn I2	-15.2	-7.6	-21.1	28.9	-15
	C15 H16 O	-3.6	-1.3	-16.5	10.7	-10.7
5.0+	C36 H24 N12 Zn I2	-18.7	-9.6	-49	44.5	-32.8
	C36 H24 N12 Zn I2	-26.2	-15	-35.3	52.7	-23.8
	C54 H36 N18 Zn2 I4	*	*	*	*	*
	C15 H16 O	-2.2	-2.2	-15.6	11.1	-8.9
	C15 H16 O	-4.0	-4.8	-24.2	28.7	-4.3
	C15 H16 O	-10.1	-5.3	-18.5	25.8	-8.1
	C15 H16 O	0	-0.1	-0.6	0	-0.7
	C36 H24 N12 Zn I2	-15	-7.3	-21.4	28.8	-14.9
	C15 H16 O	-3.6	-1.3	-16.5	10.7	-10.7

*Intermolecular interaction energies were unable to be determined with the current workflow as described above.

4. Intramolecular Energies

4.1. Conventional Single-component Crystal Structures

Table S 14: Calculated strain, conformational, and total relative energies for symmetry-independent molecules in conventional SCXRD structures, and RMS deviation of atomic positions of the experimental molecular geometry with its optimised isolated conformer. Structures are labelled with their associated CSD Refcode. For crystal structures with Z'>1, the unique molecules are labelled alphabetically.

	CSD Refcode	Space Group	Config.[1]	Mol[2]	ΔE_{strain} (kJ/mol)	RMSD (Å)	ΔE_{conf} (kJ/mol)	ΔE_{total} (kJ/mol)
BBA	2341223	*Ia*		P_A	22.53	0.433	0.03	22.56
				P_B	16.34	0.390	0.03	16.37
BBA-1-Me	2341234	*Iba2*	1	P_{A1}	17.62	0.437	0.01	17.64
			2	P_{A2}	17.34	0.437	0.01	17.35
BBA-3-Me	2341231	*P*-1		P_A	9.73	0.103	9.58	19.31
				P_B	20.94	0.446	0.03	20.97
BBA-8,12-OMe	BOWHUU (2341226)	*I*2/*a*		P_A	9.06	0.326	20.95	30.01
				P_B	23.98	0.431	3.80	27.79
BBA-(1-Me)-8,12-OMe	2341228	*P*-1		P_A	22.25	0.394	4.00	26.25
BBA-(3-Me)-8,12-OMe	2359639	*P*21/*n*		P_A	27.85	0.422	2.26	30.11
BBA-8,10,12-OMe	2341232	*P*21/*n*		P_A	28.27	0.598	5.02	33.29
BBA-1,8,12-OMe	2341224	*P*-1		P_A	26.38	0.403	4.21	30.59
				P_B	28.82	0.415	4.21	33.03

4.2. Crystalline Sponge Structures

Table S 15: Calculated strain, conformational, and total relative energies of the observed molecular geometries in crystalline sponge structures, and RMS deviation of atomic positions of the experimental molecular geometry with its optimised isolated conformer. Structures are numbered according to configuration. Multiple guests are labelled A to C.

Molecule	CSD Refcode	Space Group (Exp)	Space Group (Opt)	Config.[1]	Guest[2]	ΔE_{strain} (kJ/mol)	RMSD (Å)	ΔE_{conf} (kJ/mol)	ΔE_{total} (kJ/mol)
BBA	LIRKIK (2254203)	*C*2/*c*	*Cc*	1	CS_A	19.07	0.449	0.04	19.10
					CS_B	4.84	0.065	9.47	14.31
					CS_C	21.54	0.495	0.03	21.57
				2	CS_A	19.78	0.447	0.05	19.83
					CS_B	13.96	0.188	0.03	14.00
					CS_C	12.05	0.377	9.44	21.49
BBA-1-Me	2359637	*P*-1	*P*-1		CS_A	10.20	0.364	9.44	19.64
					CS_B	16.33	0.194	0.00	16.33
					CS_C	5.59	0.106	0.23	5.82
BBA-3-Me	LIRKOQ (2254204)	*P*-1	*P*-1	1	CS_A	18.22	0.460	0.00	18.22
					CS_B	18.55	0.418	0.00	18.55
				2	CS_A	18.94	0.456	0.00	18.94
					CS_B	6.94	0.044	12.65	19.59
				3	CS_A	18.05	0.459	0.00	18.05
					CS_B	18.37	0.466	0.00	18.37
				4	CS_A	19.47	0.463	0.00	19.47

[1] For disordered structures, multiple configurations have been considered.

[2] Structures that contain a guest molecule that sits over a rotation axis/symmetry site have been labelled numerically, e.g. A1 and A2.

					CS_B	6.85	0.056	12.59	19.44

Table S 18 (cont.)

BBA-8,12-OMe	LIRLEH (2254207)	*P*2/*n*	*Pc*	1	CS_A	19.93	0.463	3.81	23.73
					CS_B	5.29	0.169	20.99	26.28
				2	CS_A	19.88	0.423	3.81	23.70
					CS_B	5.77	0.118	20.99	26.77
BBA-(1-Me)-8,12-OMe	2359638	*C*2/*c*	*C*2/*c*		CS_A	17.27	0.428	4.08	21.35
					CS_B	23.24	0.410	4.02	27.26
BBA-(3-Me)-8,12-OMe	2359639	*C*2/*c*	*C*2/*c*		CS_A	6.89	0.109	2.28	9.17
BBA-8,10,12-OMe	LIRLIL (2254208)	*C*2/*c*	*Cc*	1	CS_A	6.03	0.108	22.53	28.55
					CS_B	23.48	0.577	5.13	28.61
				2	CS_A	6.78	0.377	21.74	28.53
					CS_B	23.40	0.549	5.13	28.52
BBA-1,8,12-OMe	2359640	*C*2/*c*	*C*2/*c*	1	CS_A	19.95	0.389	5.63	25.58
			Cc	2	CS_{B1}	6.52	0.104	5.65	12.17
			Cc	3	CS_{B2}	5.62	0.068	5.64	11.26

4.3. Comparison of CS Structures to Pharmaceutical Subset

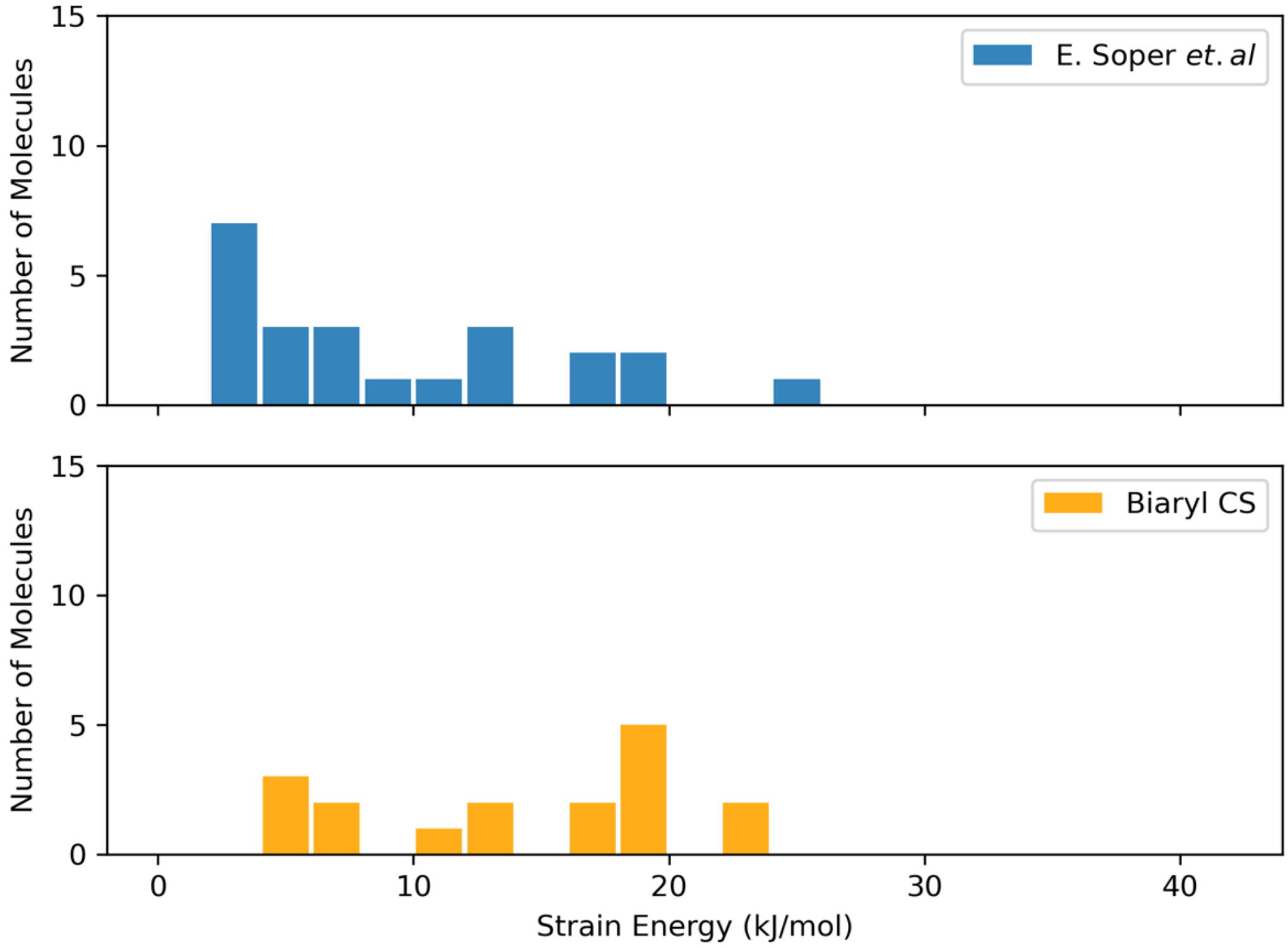

Figure S 7: Comparison of strain energies for symmetry-independent molecules in crystalline sponge structures to pharmaceutical subset in previous study (E. Soper *et. al*).

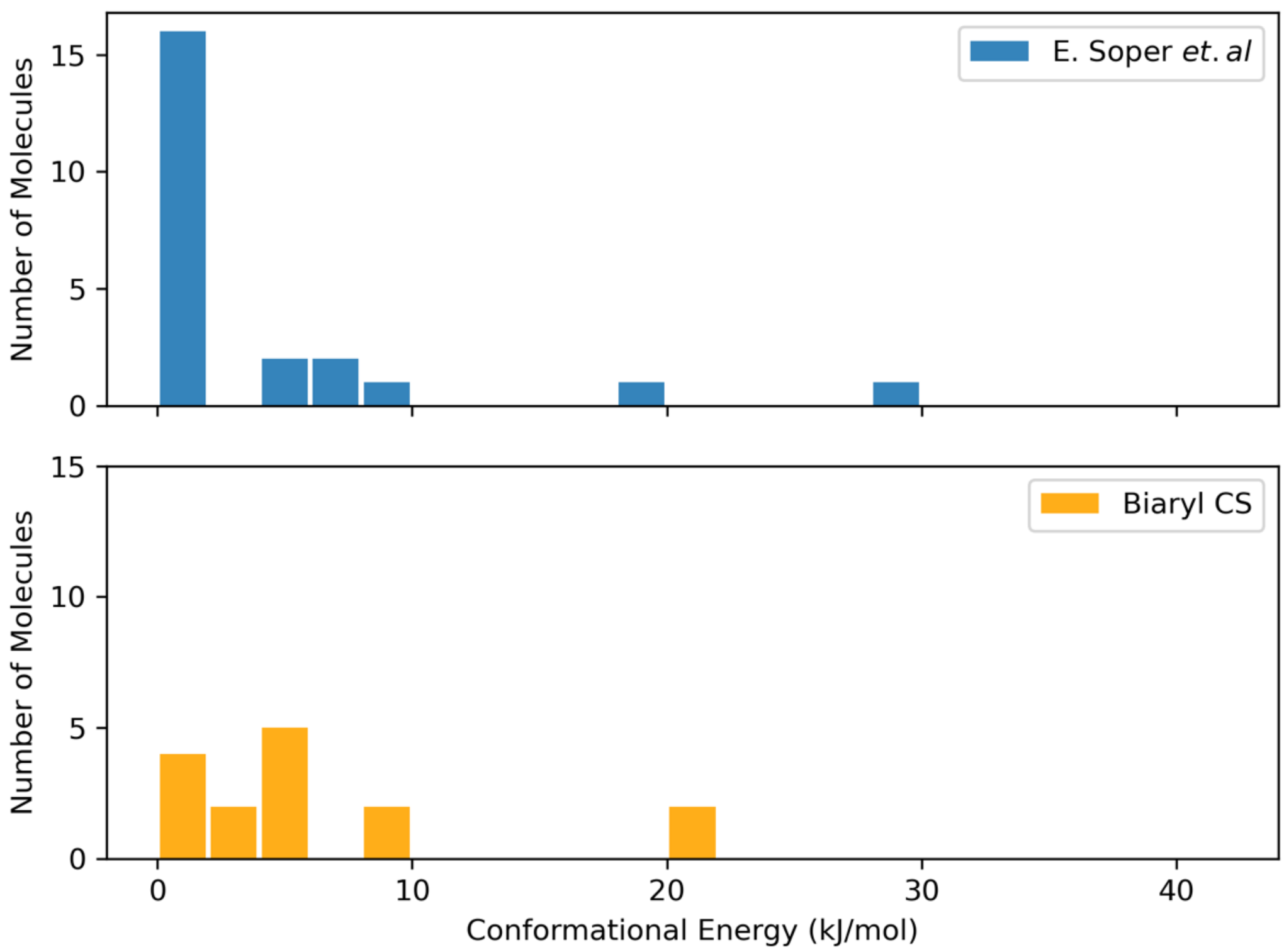

Figure S 8: Comparison of conformational energies for symmetry-independent molecules in crystalline sponge structures to pharmaceutical subset in previous study (E. Soper *et. al*).

4.4. Comparison of Conventional Crystal Structures to Pharmaceutical Subset

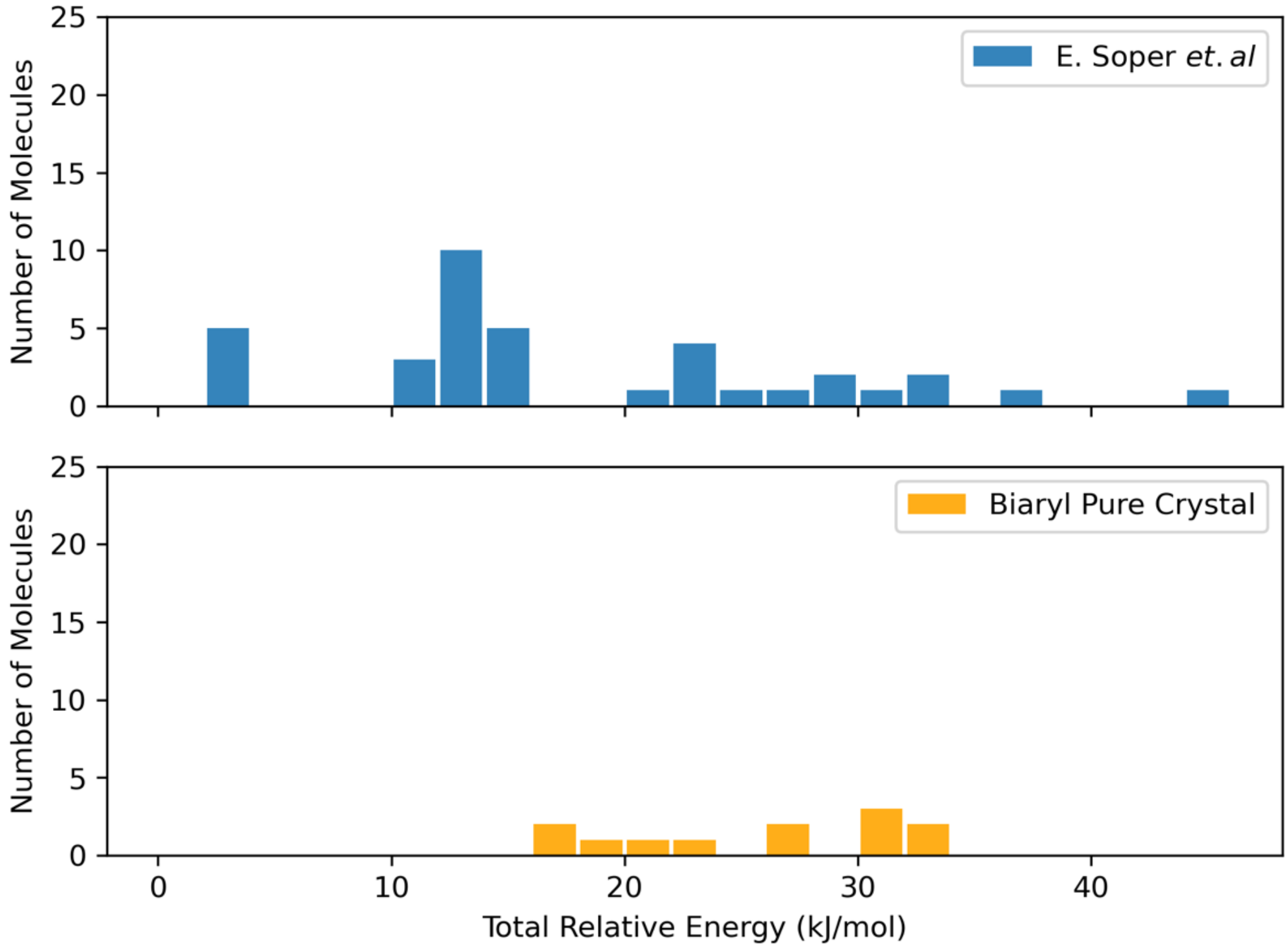

Figure S 9: Comparison of total relative intramolecular energies for symmetry-independent molecules in conventional SCXRD structures to pharmaceutical subset in previous study (E. Soper *et. al*).

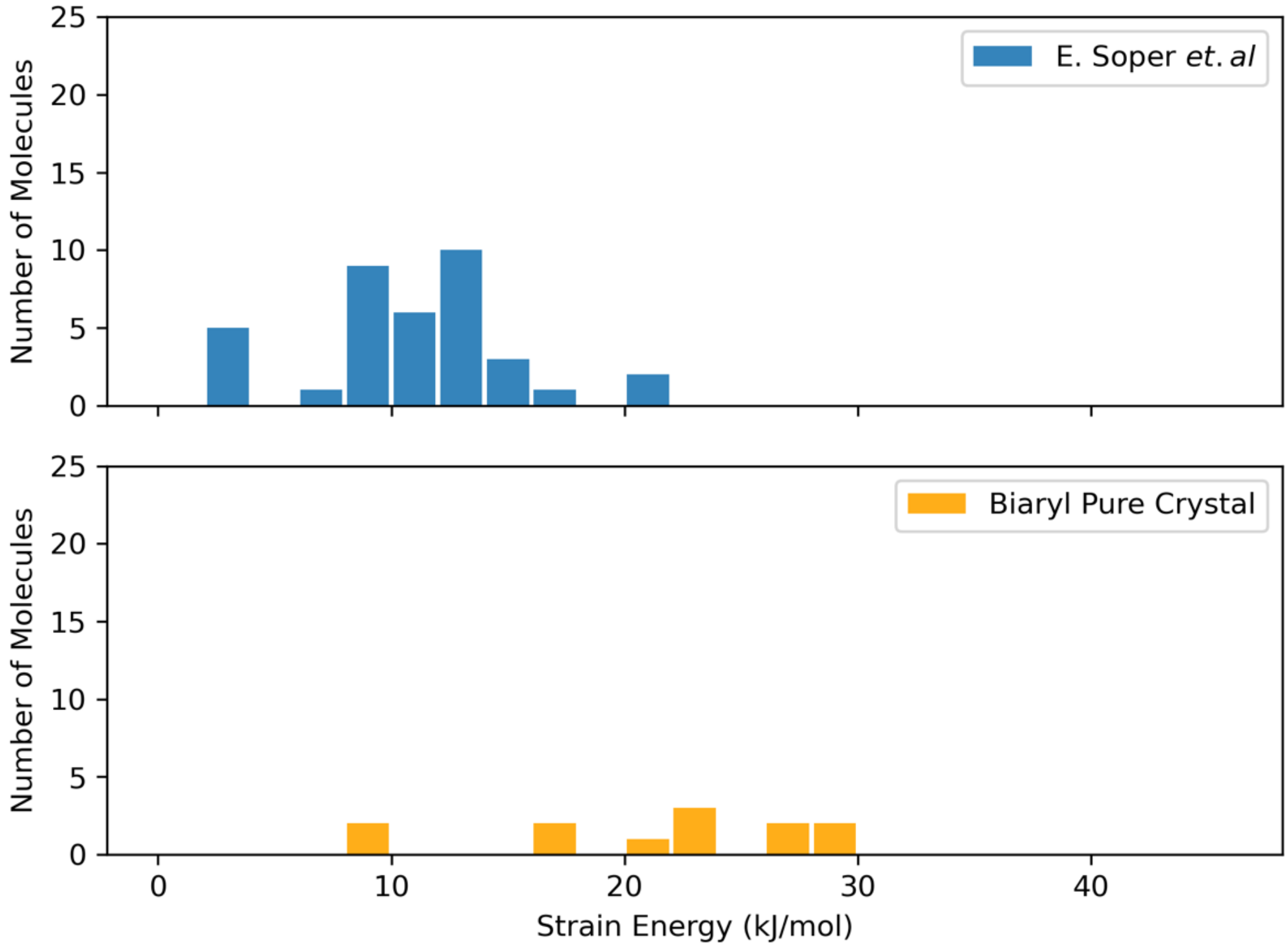

Figure S 10: Comparison of strain energies for symmetry-independent molecules in conventional SCXRD structures to pharmaceutical subset in previous study (E. Soper *et. al*).

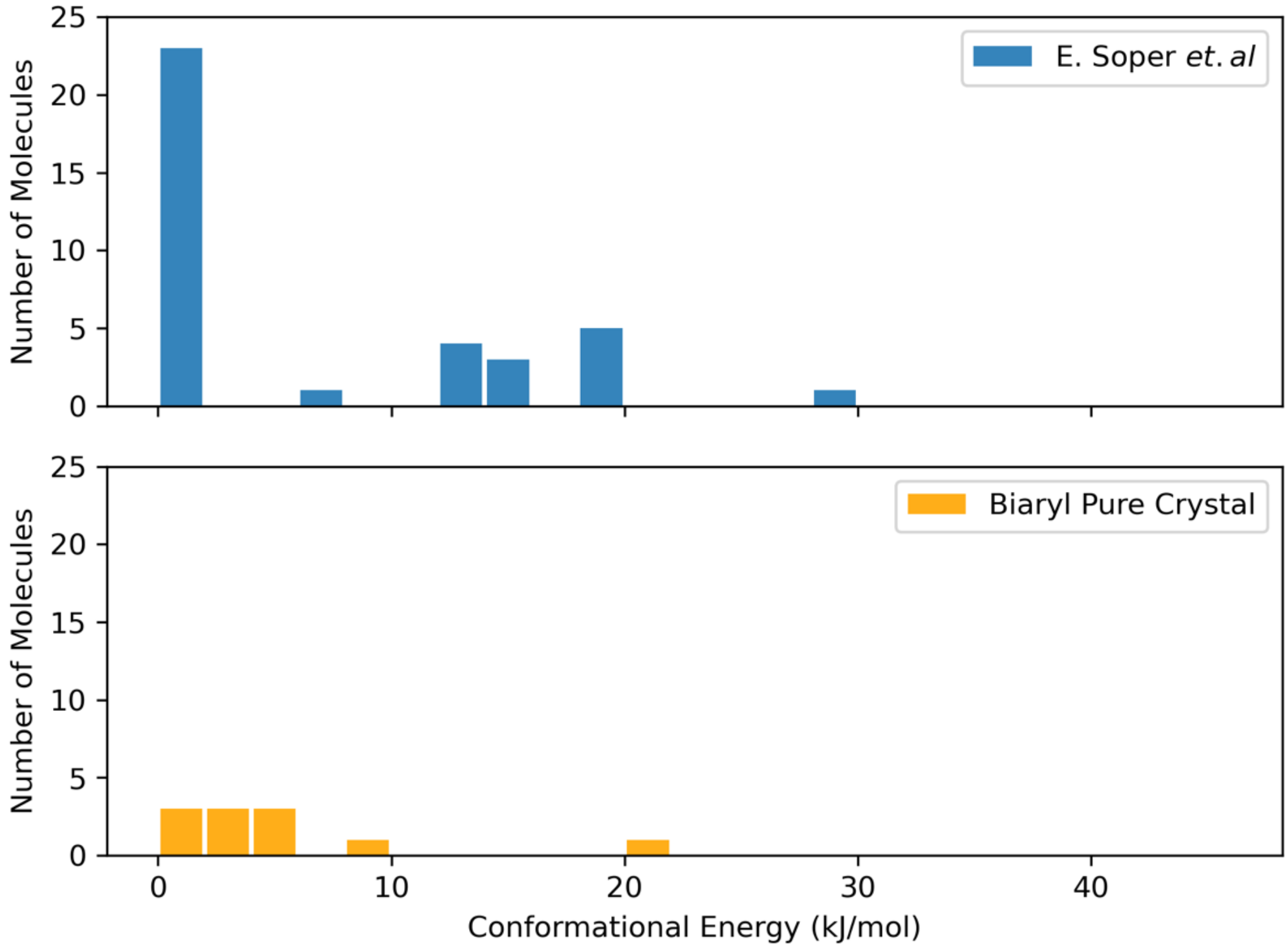

Figure S 11: Comparison of conformational energies for symmetry-independent molecules in conventional SCXRD structures to pharmaceutical subset in previous study (E. Soper *et. al*).

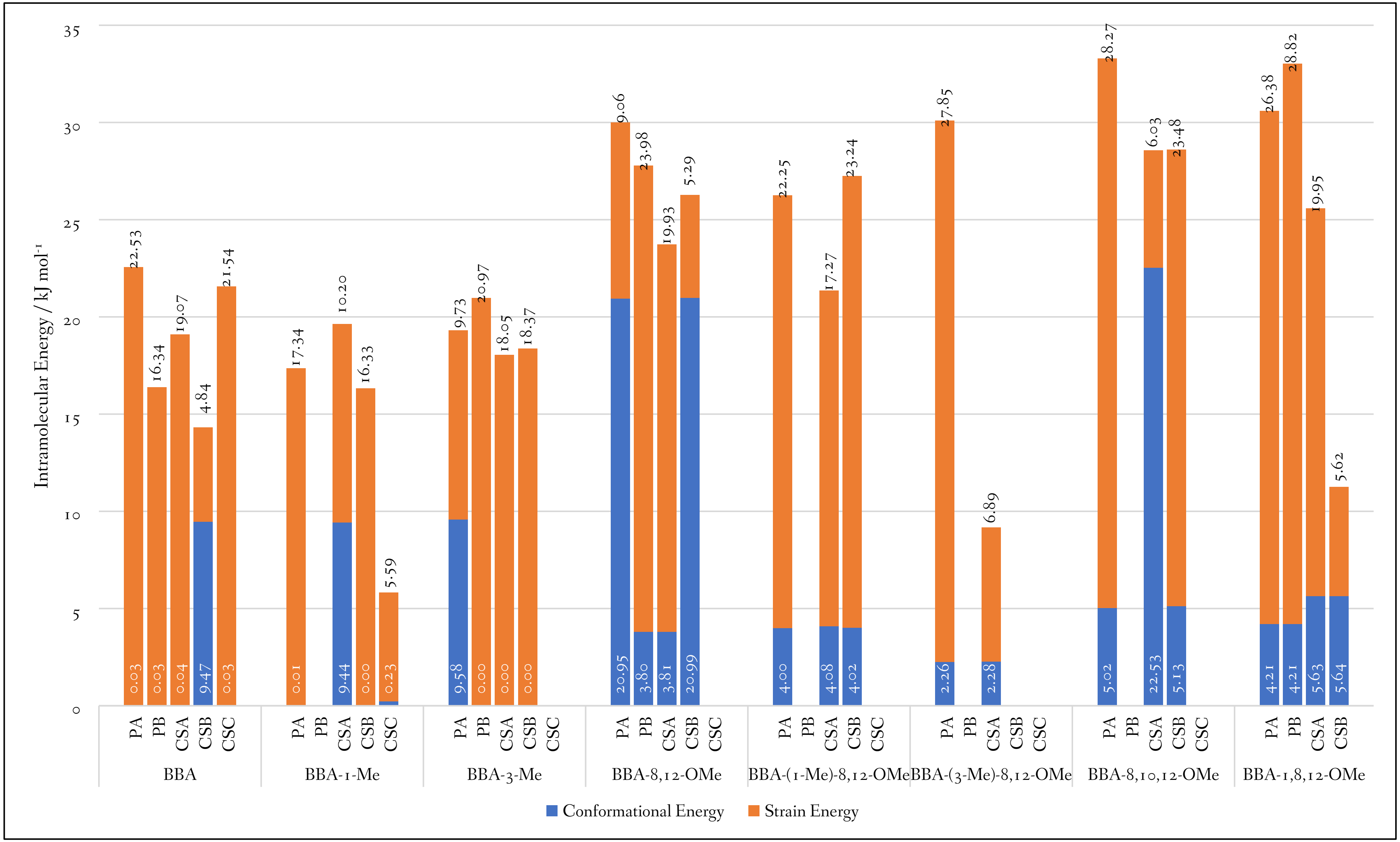

Figure S 12: Conformational and strain energies for all symmetry-independent molecules in conventional SCXRD and CS structures.

5. Geometric Changes Influencing Intramolecular Energy for Biaryl Molecules

The geometric changes which influence relative energies can be investigated with comparison of graphical overlays and root-mean-square-deviation (RMSD) values for experimental structures, as illustrated in Figure S 13.

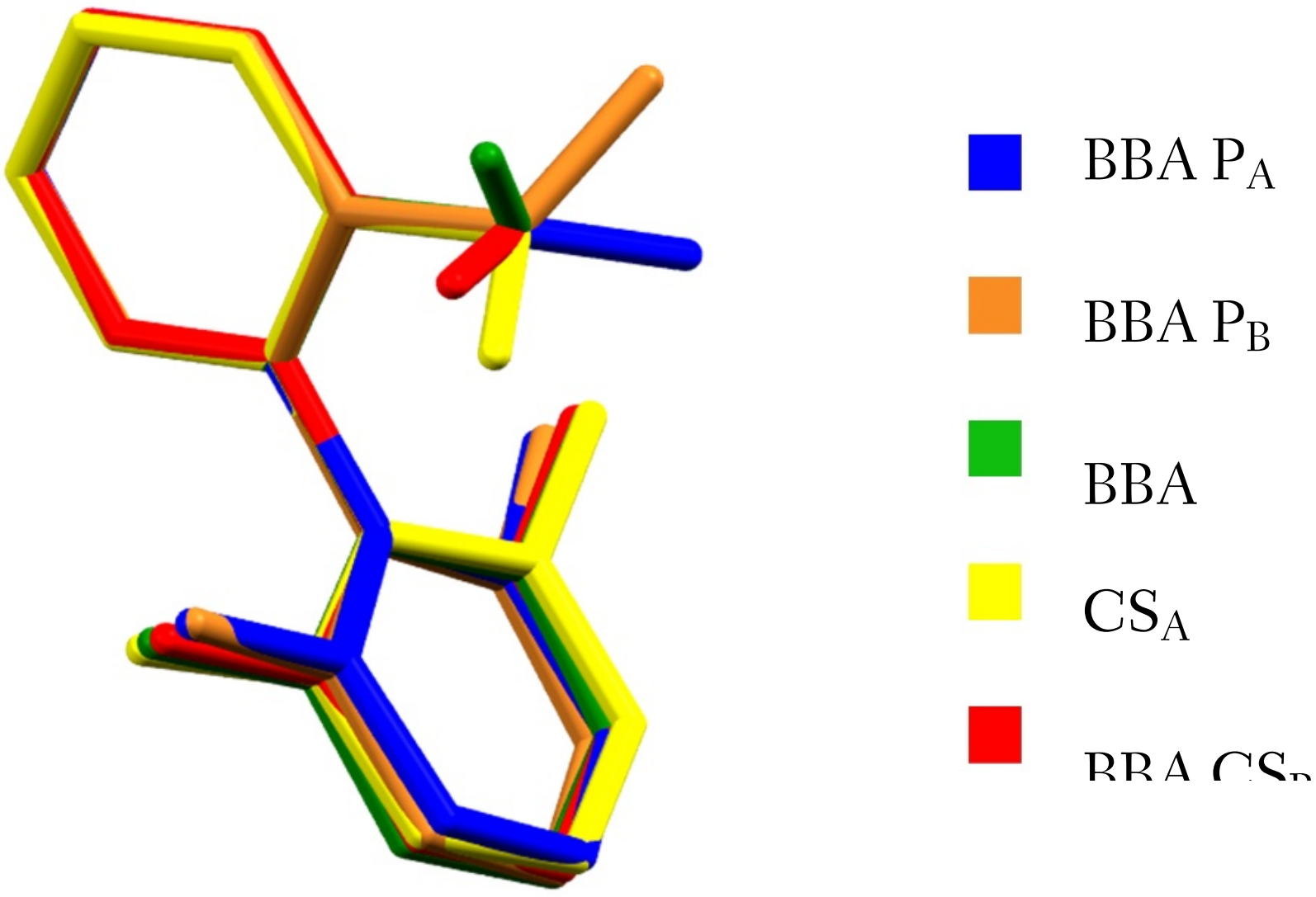

Figure S 13: Exemplary overlay of experimental geometries for BBA (hydrogen atoms omitted for clarity).

Movement of the benzyl alcohol ($-CH_2OH$) arm is the most evident factor visually, but rotation around the central aryl-aryl bond is also significant for relative energies. Notably, stable gas phase conformers tend to adopt intramolecular oxygen ··· hydrogen (O ··· H) contacts when methoxy groups are present and the resultant movement of methoxy-methyl groups have a relatively low energetic impact.

These intramolecular hydrogen bonds are observed only once in the obtained CS data for BBA-(3-Me)-8,12-OMe, a low efficiency exchanged analyte with a single guest molecule resolved at 11.0% occupancy. The remaining CS structures and all conventional SCXRD show preference of the biaryl molecules for intermolecular contact in the solid state. The difficulty of accurately determining hydrogen atom positions in CS structures, due to the high residual electron density within framework pores, may also be considered a contributing factor to the lack of observed intramolecular contacts. However, it should be noted that most guests do not adopt conformations which would enable self-interaction.

6. Intermolecular Interaction Energies

6.1. Conventional SCXRD

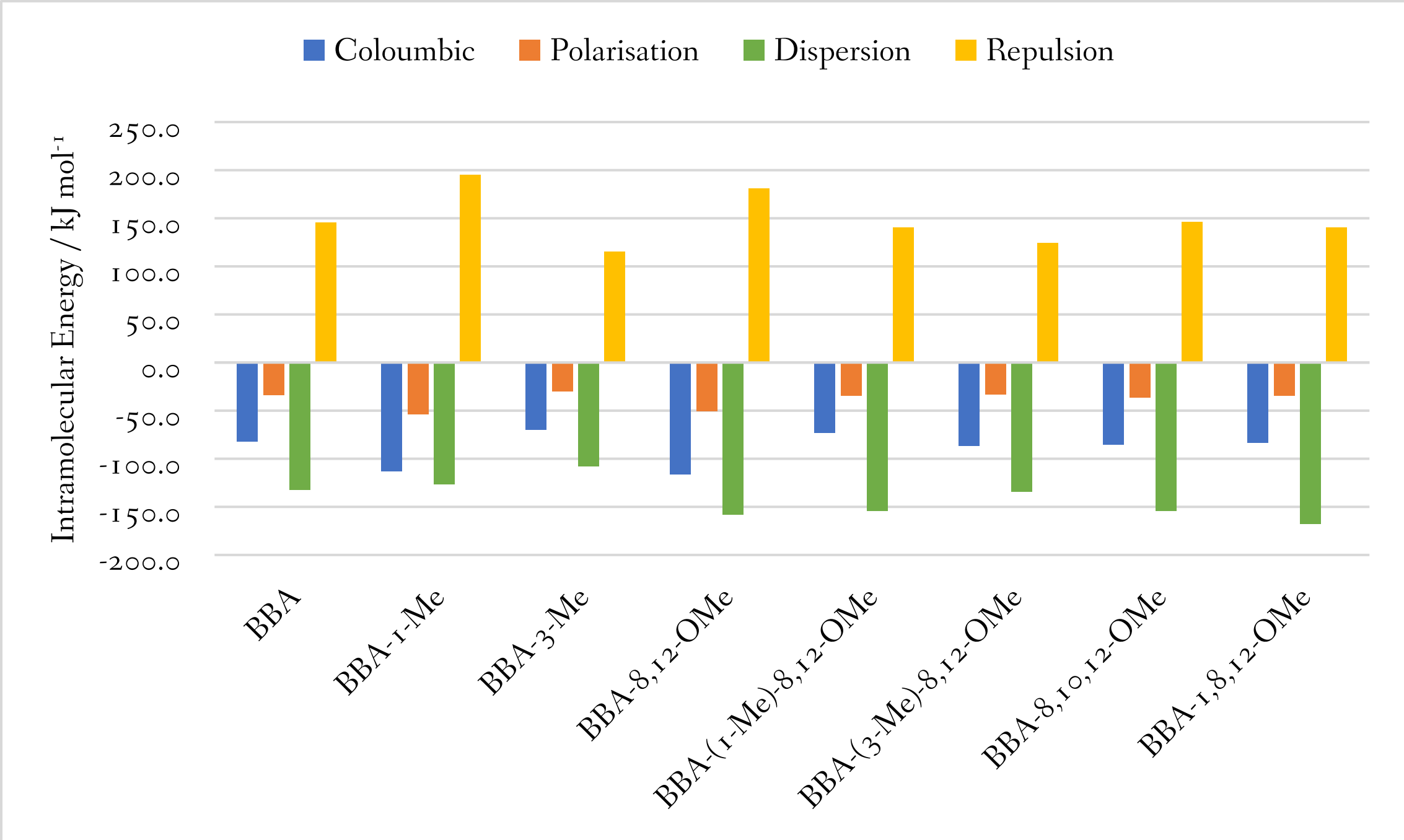

Figure S 14: Intermolecular interaction energy components for conventional SCXRD structures.

Table S 16: Intermolecular interaction energy components for conventional SCXRD structures.

	E_{COU}	E_{POL}	E_{DISP}	E_{REP}	E_{DIP}	E_{TOT}
BBA	-81.8	-33.8	-132.1	145.8	-1.0	-102.9
BBA-1-Me	-112.8	-53.7	-126.4	195.2	-1.5	-99.2
BBA-3-Me	-69.8	-29.7	-108.0	115.4	0.0	-92.1
BBA-8,12-OMe	-116.5	-50.5	-158.3	181.2	0.0	-144.1
BBA-(1-Me)-8,12-OMe	-73.4	-34.4	-154.3	140.6	0.0	-121.5
BBA-(3-Me)-8,12-OMe	-86.4	-32.9	-134.2	124.1	0.0	-129.4
BBA-8,10,12-OMe	-85.5	-36.2	-154.5	146.4	0.0	-129.8
BBA-1,8,12-OMe	-83.1	-34.2	-167.9	140.3	0.0	-144.9

Where E_{DIP} is the cell dipole and E_{TOT} is the total intermolecular interaction energy.

6.2. Crystalline Sponge

6.2.1. Overview

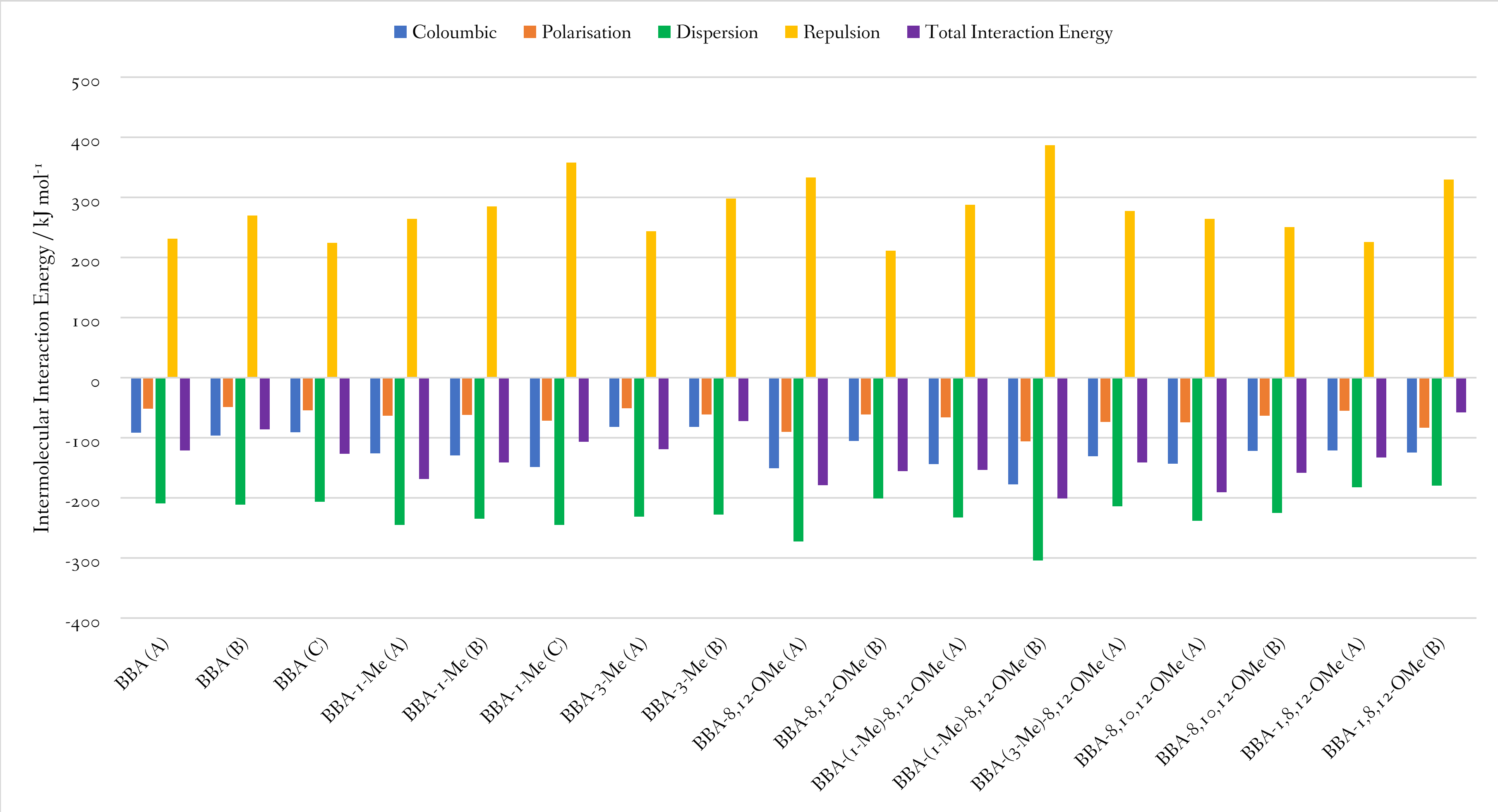

Figure S 15: Intermolecular interaction energy components for symmetry-independent molecules in crystalline sponge structures.

Table S 17: Intermolecular interaction energy components for symmetry-independent molecules in crystalline sponge structures.

		Intermolecular Interaction Energies / kJ mol^{-1}				
		E_{COU}	E_{POL}	E_{DISP}	E_{REP}	E_{TOT}
BBA	CS_A	-91.1	-51.3	-208.8	230.7	-120.5
	CS_B	-95.8	-48.7	-210.8	269.7	-85.6
	CS_C	-90.2	-53.9	-206.4	224.2	-126.3
BBA-1-Me	CS_A	-125.4	-62.7	-244.4	263.9	-168.6
	CS_B	-129.2	-61.6	-234.2	284.3	-140.7
	CS_C	-148.1	-71.1	-244.8	357.9	-106.1
BBA-3-Me	CS_A	-81.4	-50.4	-230.7	243.6	-118.9
	CS_B	-81.3	-61.0	-227.3	297.8	-71.8
BBA-8,12-OMe	CS_A	-150.5	-89.5	-272.0	333.1	-178.9
	CS_B	-104.8	-60.9	-200.7	211.0	-155.4
BBA-(1-Me)-8,12-OMe	CS_A	-143.3	-65.4	-232.1	287.5	-153.3
	CS_B	-177.2	-105.9	-304.1	386.8	-200.4
BBA-(3-Me)-8,12-OMe	CS_A	-130.6	-73.3	-214.0	277.0	-140.9
BBA-8,10,12-OMe	CS_A	-142.8	-74.0	-237.6	264.0	-190.4
	CS_B	-121.2	-62.9	-224.4	250.3	-158.2
BBA-1,8,12-OMe	CS_A	-121.1	-54.7	-182.2	225.5	-132.5
	CS_B	-124.0	-83.1	-179.3	329.1	-57.3

6.2.2. Host – Guest and Guest – Guest Contributions

Table S 18: Intermolecular interaction energy components separated into host – guest and guest – guest contributions for symmetry-independent molecules in crystalline sponge structures.

			Intermolecular Interaction Energies / kJ mol^{-1}				
			E_{COU}	E_{POL}	E_{DISP}	E_{REP}	E_{TOT}
BBA	CS_A	H ··· G	-71.2	-37.7	-134	154.4	-88.5
		G ··· G	-19.9	-13.6	-74.8	76.3	-32.0
	CS_B	H ··· G	-61.7	-33.8	-148.2	155.0	-80.7
		G ··· G	-44.1	-20.2	-81.1	140.5	-4.9
	CS_C	H ··· G	-71.5	-43.8	-151.6	164.6	-102.3
		G ··· G	-18.7	-10.1	-54.8	59.6	-24.0
BBA-1-Me	CS_A	H ··· G	-91.7	-44.7	-152.9	156.3	-133.0
		G ··· G	-23.7	-11.3	-63.3	72.3	-26.0
	CS_B	H ··· G	-108.0	-51.3	-169.6	219.9	-109.0
		G ··· G	-17.9	-8.6	-46.9	50.9	-22.5
	CS_C	H ··· G	-123.8	-57.8	-174.6	269.7	-86.5
		G ··· G	-0.8	-0.7	-11.8	4.4	-8.9
BBA-3-Me	CS_A	H ··· G	-58.9	-35.7	-138.7	155.9	-77.4
		G ··· G	-17.0	-12.2	-73.1	68.8	-33.5
	CS_B	H ··· G	-50.7	-38.8	-144.0	181.2	-52.3
		G ··· G	-8.3	-7.1	-31.3	32.8	-13.9
BBA-8,12-OMe	CS_A	H ··· G	-86.6	-50.7	-160.7	188.2	-109.8
		G ··· G	-56.7	-33.3	-73.3	113.5	-49.8
	CS_B	H ··· G	-99.3	-56.4	-166.7	186.0	-136.4
		G ··· G	0.0	0.0	0.0	0.0	0.0
BBA-(1-Me)-8,12-OMe	CS_A	H ··· G	-118.5	-53.0	-166.5	225.7	-112.3
		G ··· G	-23.9	-11.8	-61.7	58.9	-38.5
	CS_B	H ··· G	-85.4	-54.8	-169.3	202.8	-106.7
		G ··· G	-91.8	-51.1	-134.8	184.0	-93.7
BBA-(3-Me)-8,12-OMe	CS_A	H ··· G	-58.4	-40.3	-140.0	131.3	-107.4
		G ··· G	-72.2	-33.0	-74.0	145.7	-33.5

Table S18 (cont.)

BBA-8,10,12-OMe	CS_A	H···G	-123.9	-64.6	-193.7	222.8	-159.4
		G···G	-18.8	-9.3	-41.5	40.8	-28.8
	CS_B	H···G	-97.5	-50.2	-156.4	191.2	-112.9
		G···G	-18.8	-9.3	-41.5	40.8	-28.8
BBA-1,8,12-OMe	CS_A	H···G	-121.1	-53.6	-172.5	223.6	-123.6
		G···G	0.0	-1.1	-9.7	1.9	-8.9
	CS_B	H···G	-124	-83.1	-179.3	329.1	-57.3
		G···G	0.0	0.0	0.0	0.0	0.0

7. Distribution of Conformational and Strain Energies

7.1. Conventional SCXRD

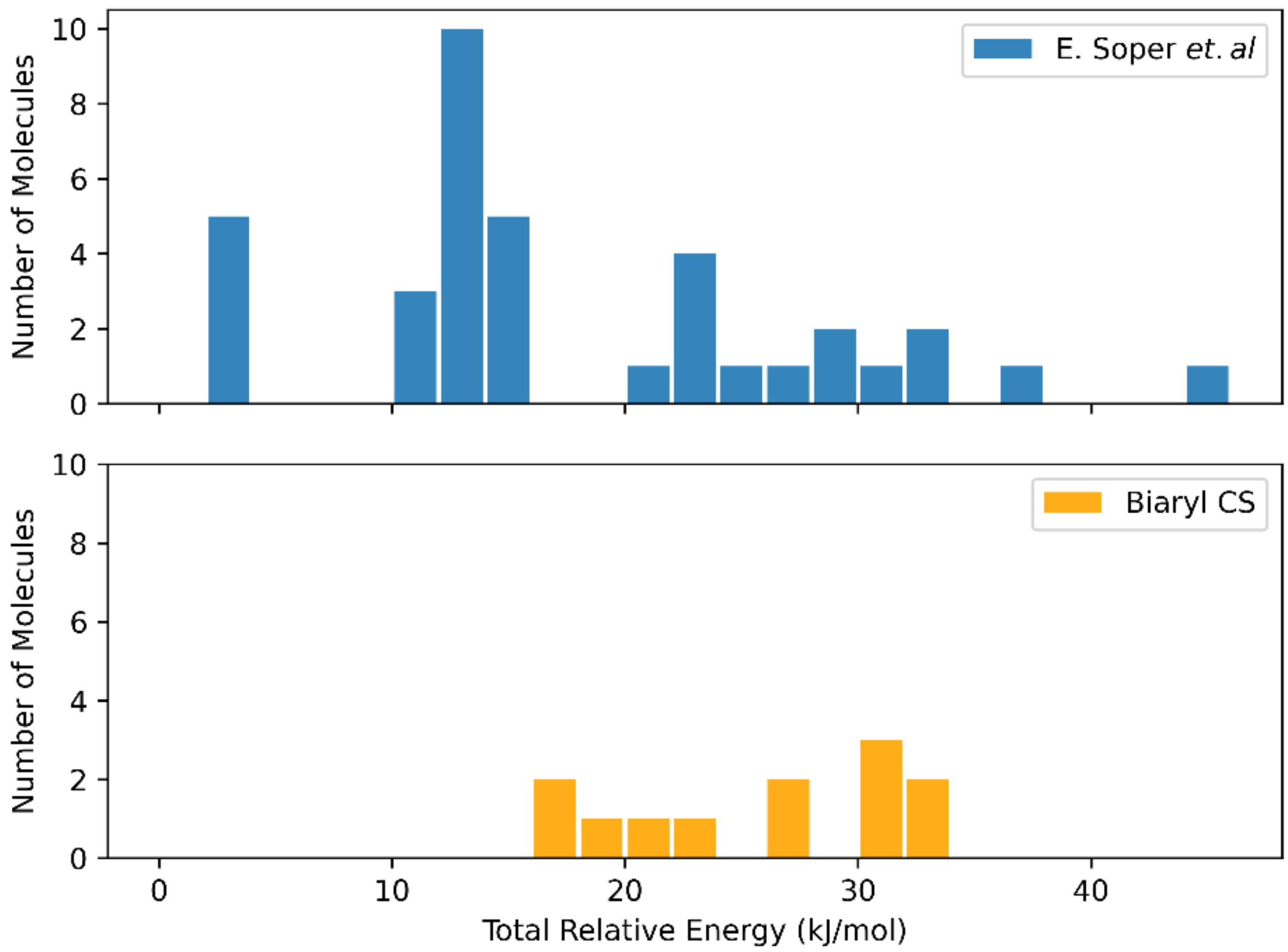

Figure S 16: Comparison of total relative intramolecular energies for symmetry-independent molecules in conventional SCXRD structures to molecules previously investigated by Soper *et al.*

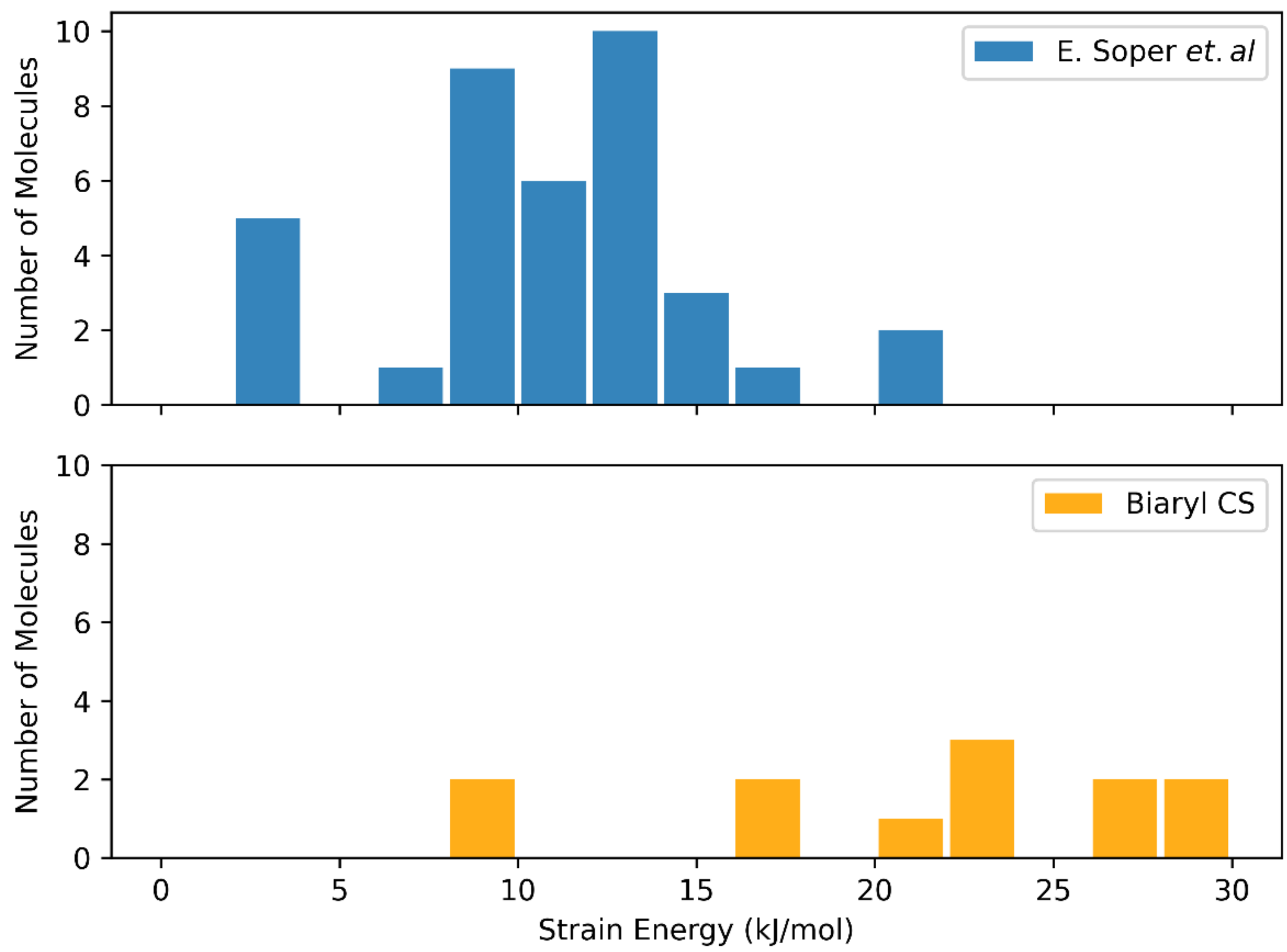

Figure S 17: Comparison of strain energies for symmetry-independent molecules in conventional SCXRD structures to molecules previously investigated by Soper *et al.*

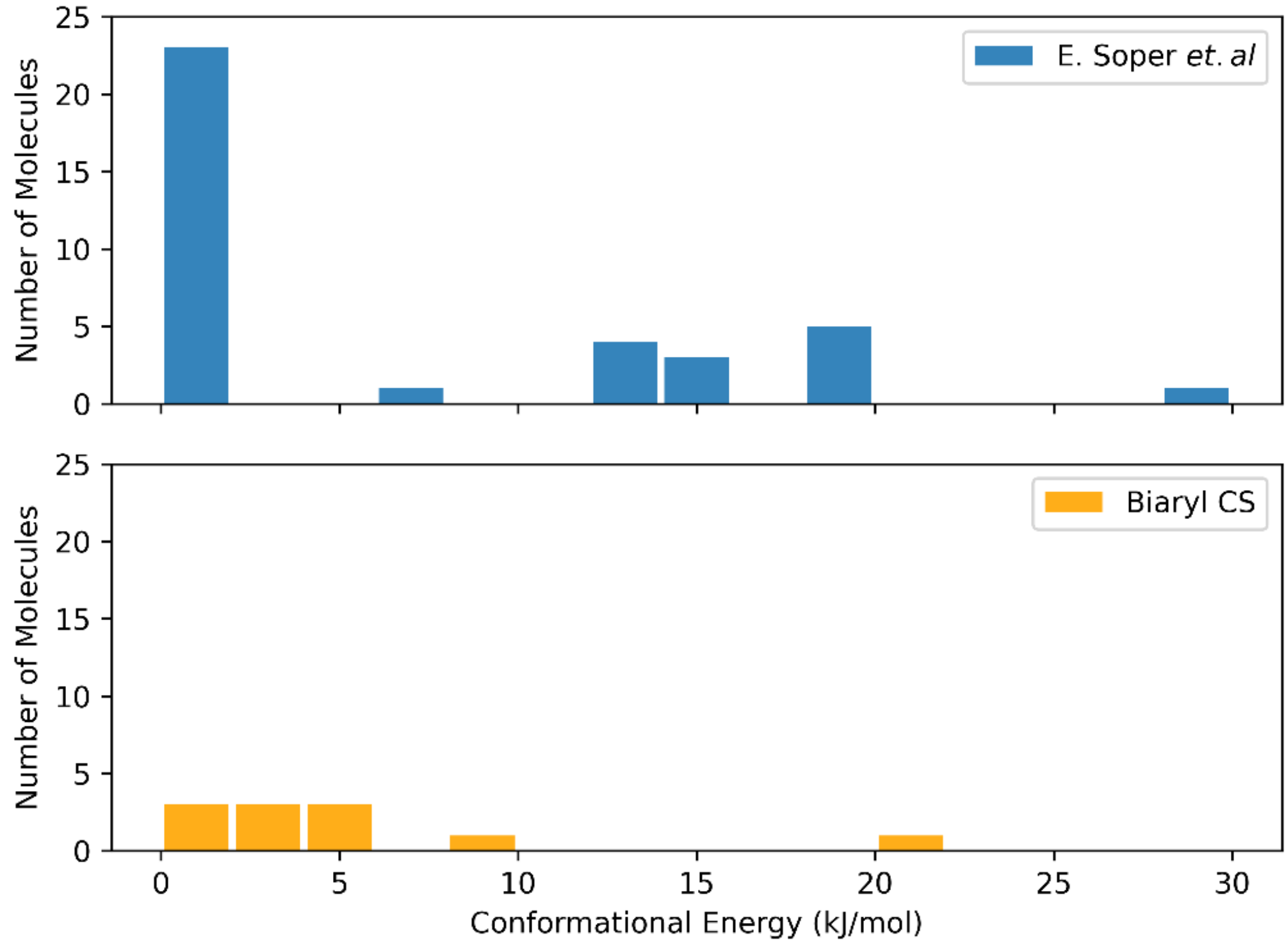

Figure S 18: Comparison of conformational energies for symmetry-independent molecules in conventional SCXRD structures to molecules previously investigated by Soper *et al.*

7.2. Crystalline Sponge Structures

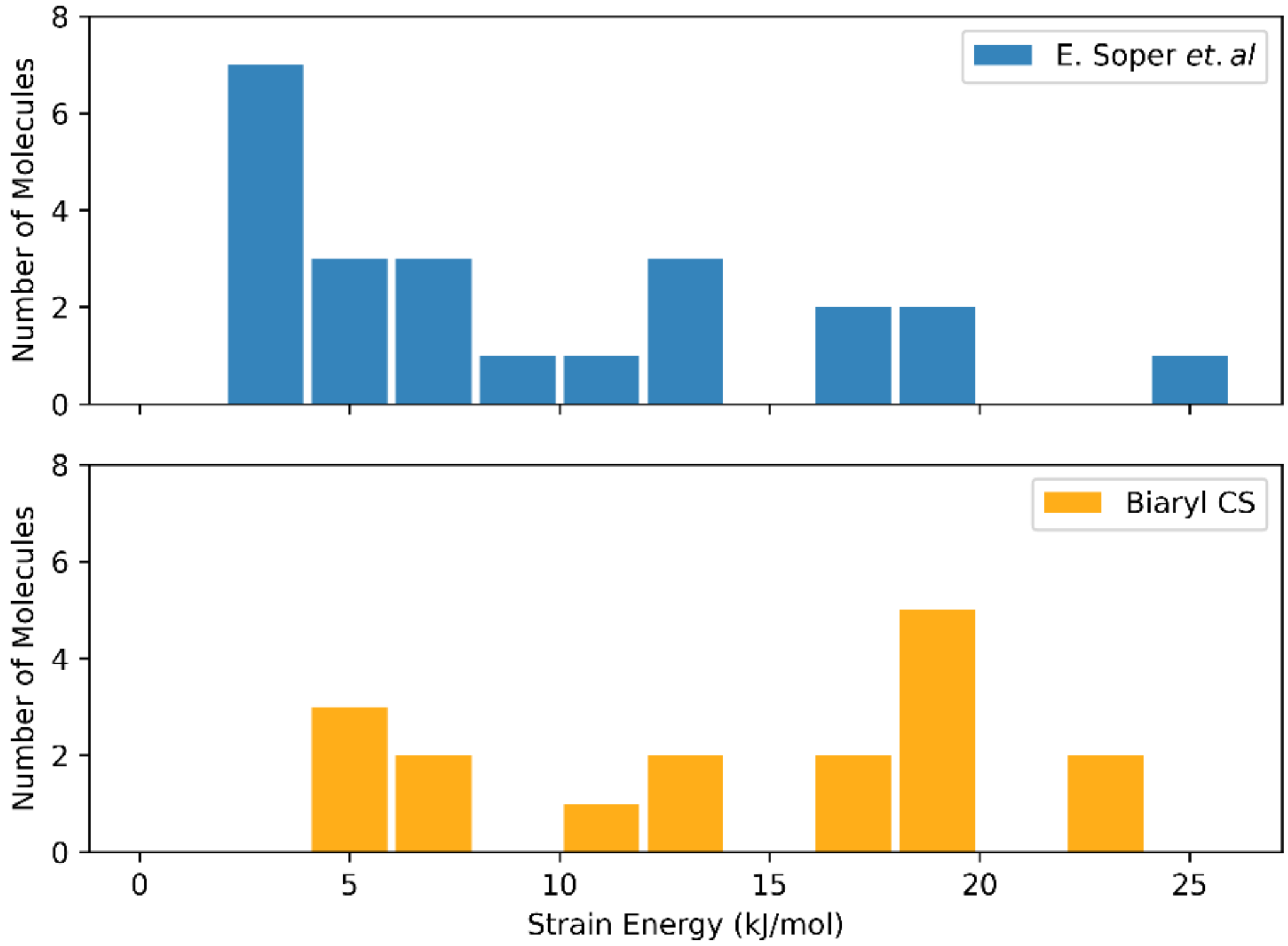

Figure S 19: Comparison of strain energies for symmetry-independent molecules in crystalline sponge structures to molecules previously investigated by Soper *et al.*

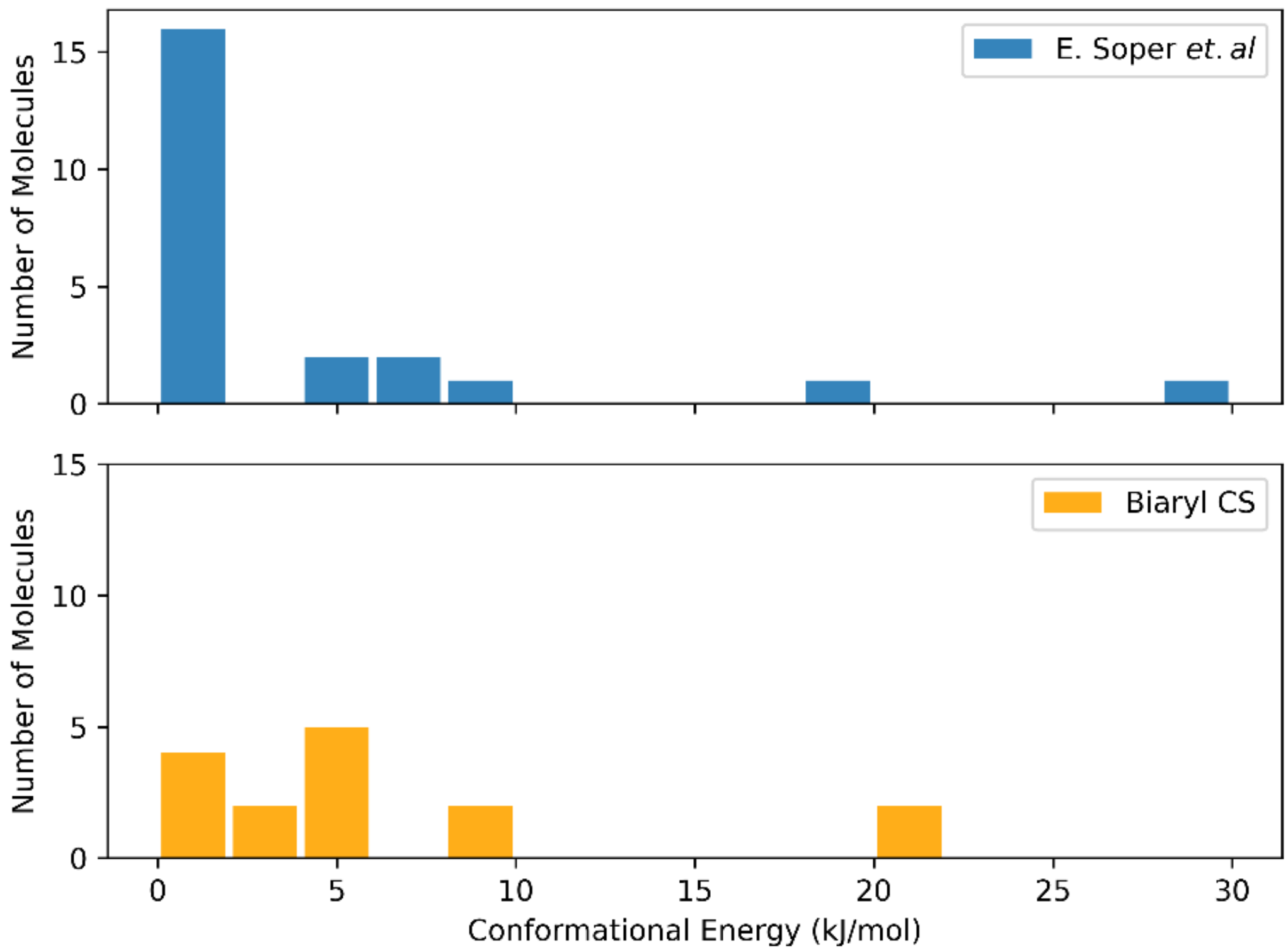

Figure S 20: Comparison of conformational energies for symmetry-independent molecules in crystalline sponge structures to molecules previously investigated by Soper *et al.*

8. NMR Spectra and Tabulation

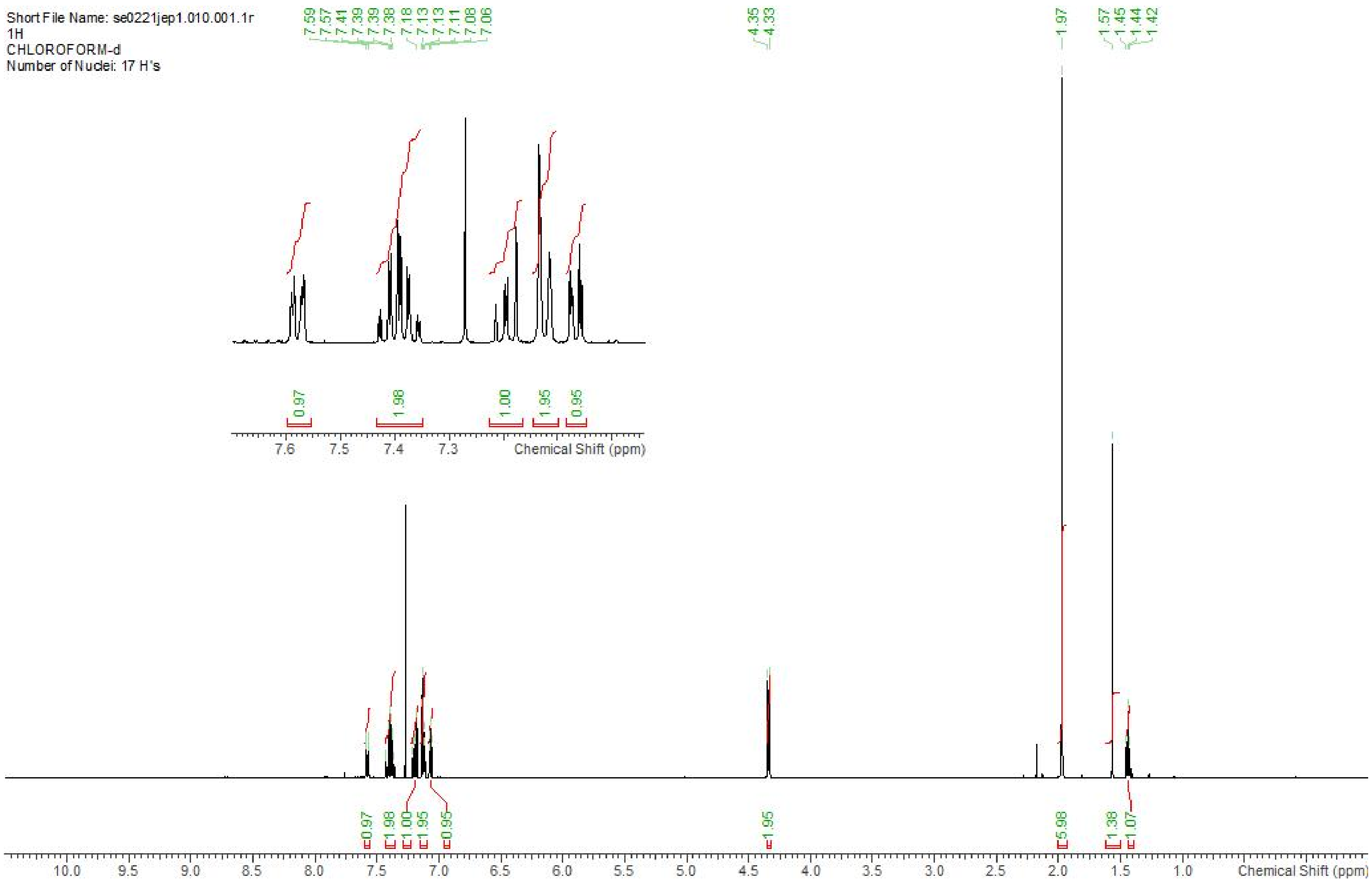

Figure S 21: ^{1}H-NMR spectrum of BBA (400 MHz, $CDCl_3$).

Table S 19: Tabulated ^{1}H-NMR spectrum of BBA (400 MHz, $CDCl_3$).

Peak Shift / ppm	Normalised Integration	Observed Splitting Pattern	Chemical Group	Atoms Assigned
1.44	1.07	*Triplet*	-OH	H1A
1.97	5.98	*Singlet*	$-CH_3$	H14A – 14C<br>H15A – 15C
4.34	1.95	*Doublet*	$-CH_2$	H13A – 13B
7.07	0.95	*Multiplet*	Aromatic -CH	H10
7.10 – 7.15	1.95	*Multiplet*	Aromatic -CH	H9 + H11
7.37	1.00	Apparent *td*	Aromatic -CH	H1 – H3
7.41	1.98	Apparent *td*	Aromatic -CH	H1 – H3
7.58	0.97	*Multiplet*	Aromatic -CH	H6

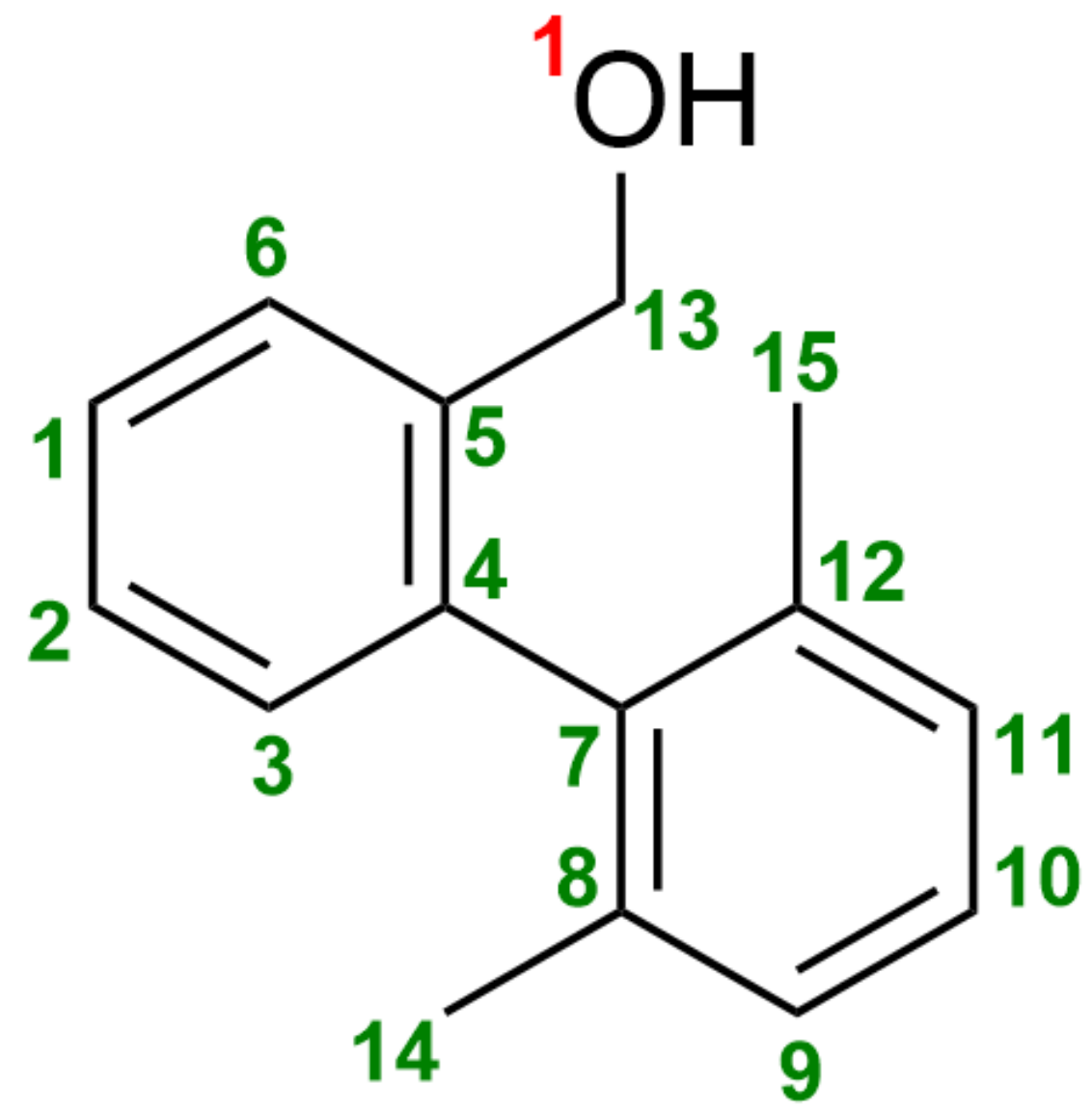

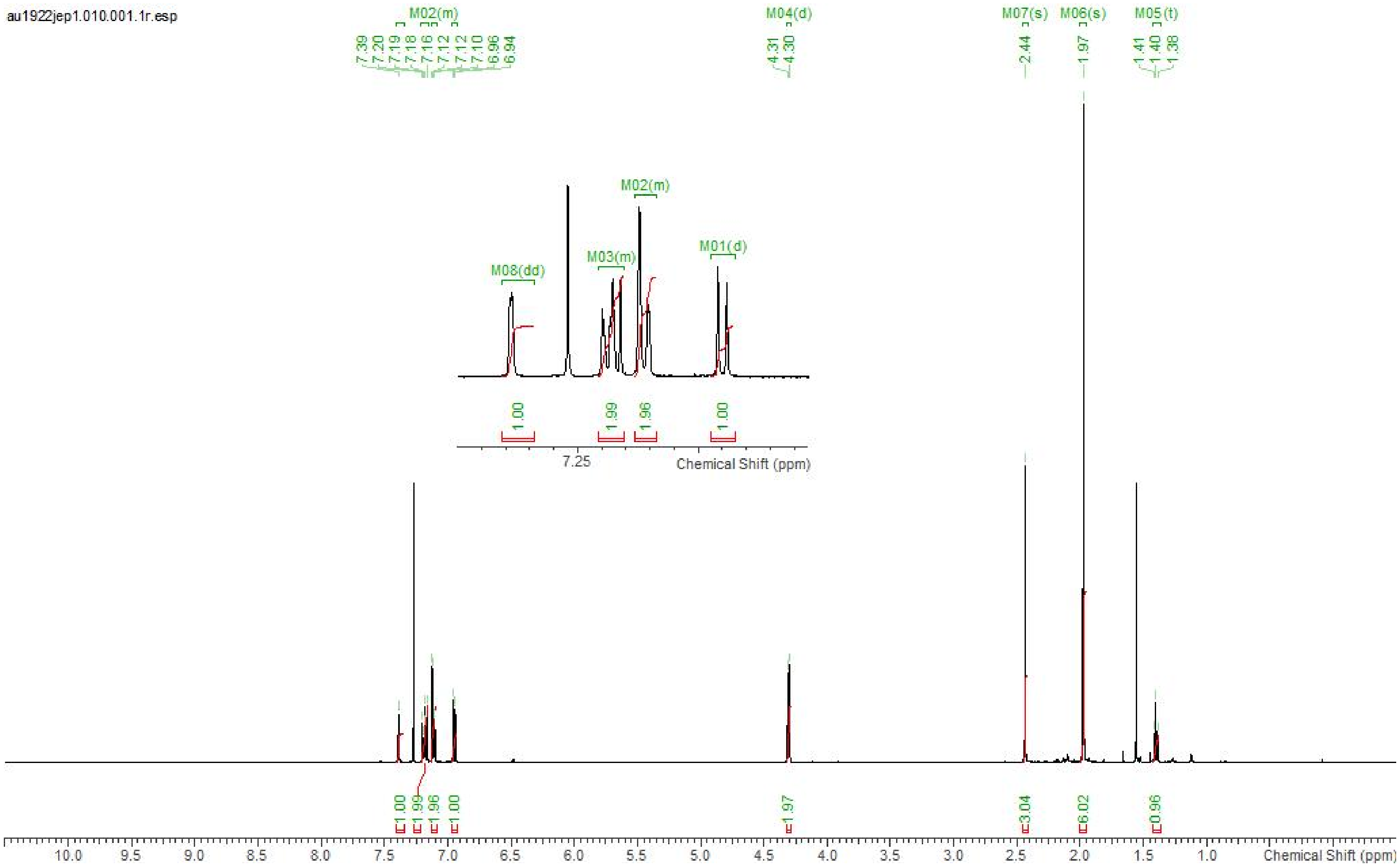

Figure S 22: ^{1}H-NMR spectrum of BBA-1-Me (400 MHz, $CDCl_3$).

Table S 20: Tabulated ¹H-NMR spectrum of BBA-1-Me (400 MHz, $CDCl_3$).

Peak Shift / ppm	Normalised Integration	Observed Splitting Pattern	Chemical Group	Atoms Assigned
1.40	0.96	*Triplet*	-OH	H1A
1.97	6.02	*Singlet*	$-CH_3$	H14A – 14C<br>H15A – 15C
2.44	3.04	*Singlet*	$-CH_3$	H16A – 16C
4.31	1.97	*Doublet*	$-CH_2$	H13A – 13B
6.95	1.00	*Doublet*	Aromatic -CH	H10
7.09 – 7.13	1.96	*Multiplet*	Aromatic -CH	H9 + H11
7.15 – 7.21	1.99	*Multiplet*	Aromatic -CH	H2 + H3
7.39	1.00	*dd*	Aromatic -CH	H6

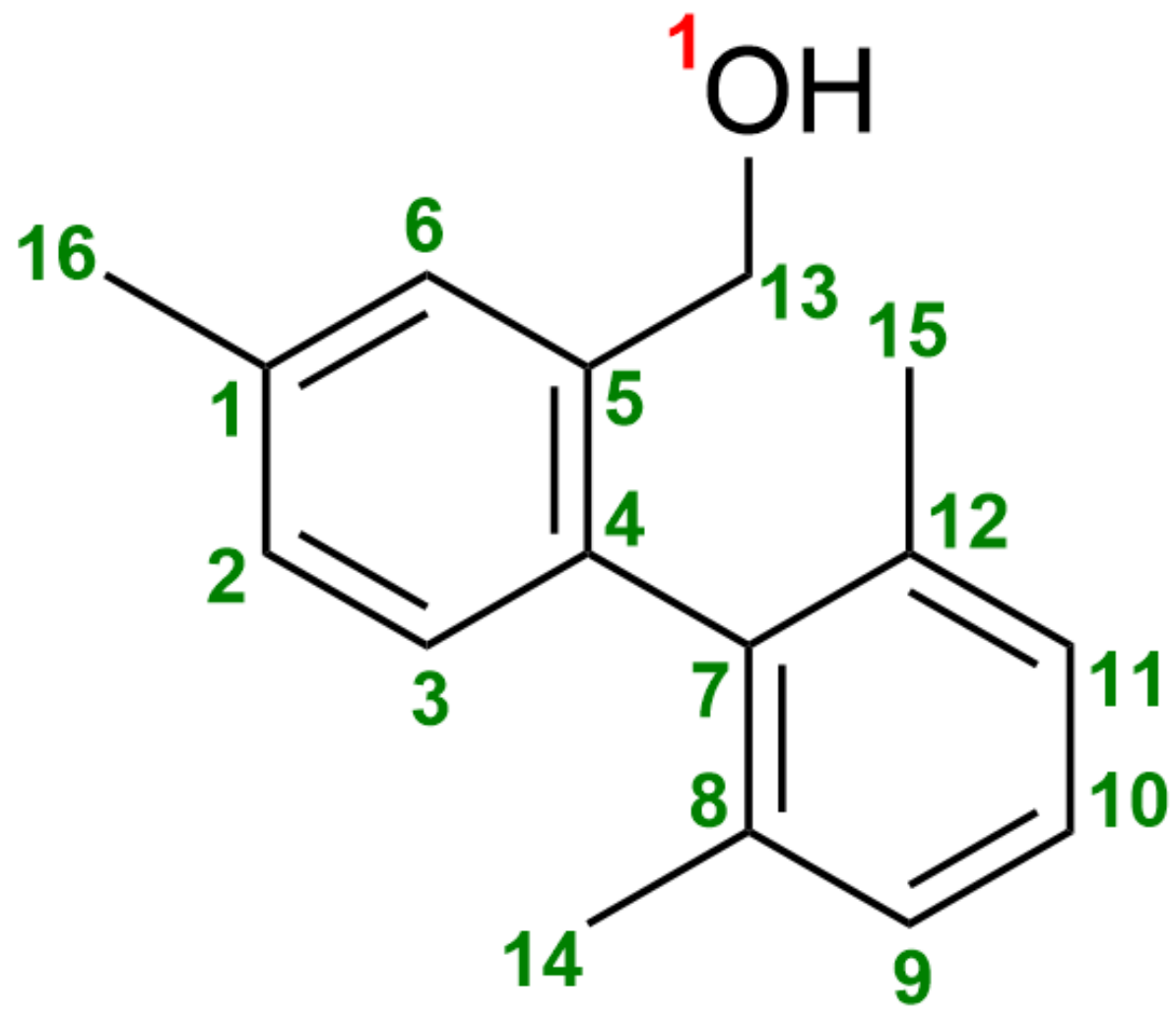

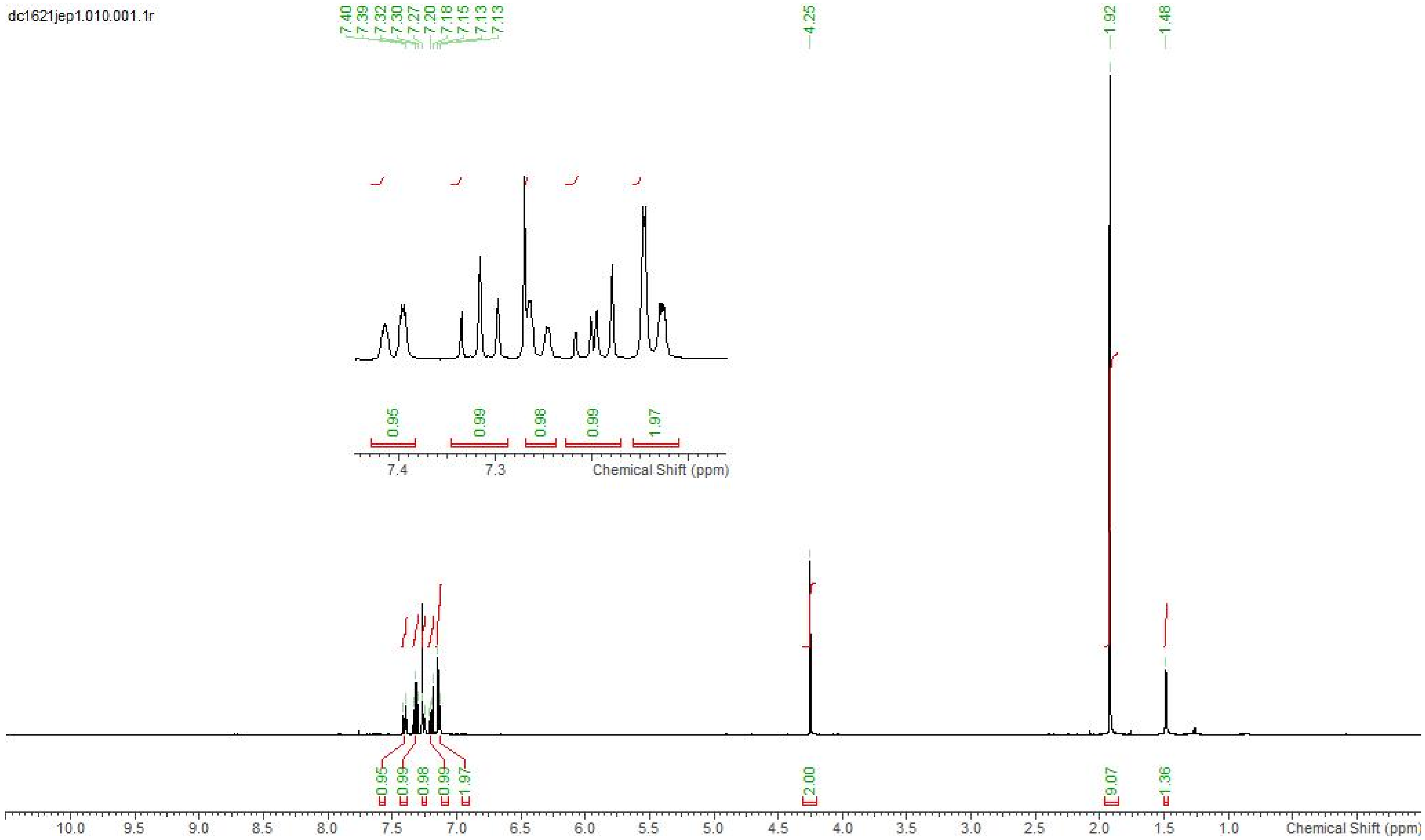

Figure S 23: ^{1}H-NMR spectrum of BBA-3-Me (400 MHz, $CDCl_3$).

Table S 21: Tabulated ^{1}H-NMR spectrum of BBA-3-Me (400 MHz, $CDCl_3$).

Peak Shift / ppm	Normalised Integration	Observed Splitting Pattern	Chemical Group	Atoms Assigned
1.39	1.36	*Singlet*	-OH	H1A
1.92	9.07	*Singlet*	$-CH_3$	H14A – 14C<br>H15A – 15C<br>H16A – 16C
4.25	2.00	*Singlet*	$-CH_2$	H13A – 13B
7.11 – 7.16	1.97	*Multiplet*	Aromatic -CH	H9 + H11
7.20	0.99	*dd*	Aromatic -CH	H10
7.26	0.98	*Multiplet*	Aromatic -CH	H1 / H2 / H6
7.32	0.99	Apparent *t*	Aromatic -CH	H1 / H2 / H6
7.41	0.95	*ddd*	Aromatic -CH	H1 / H2 / H6

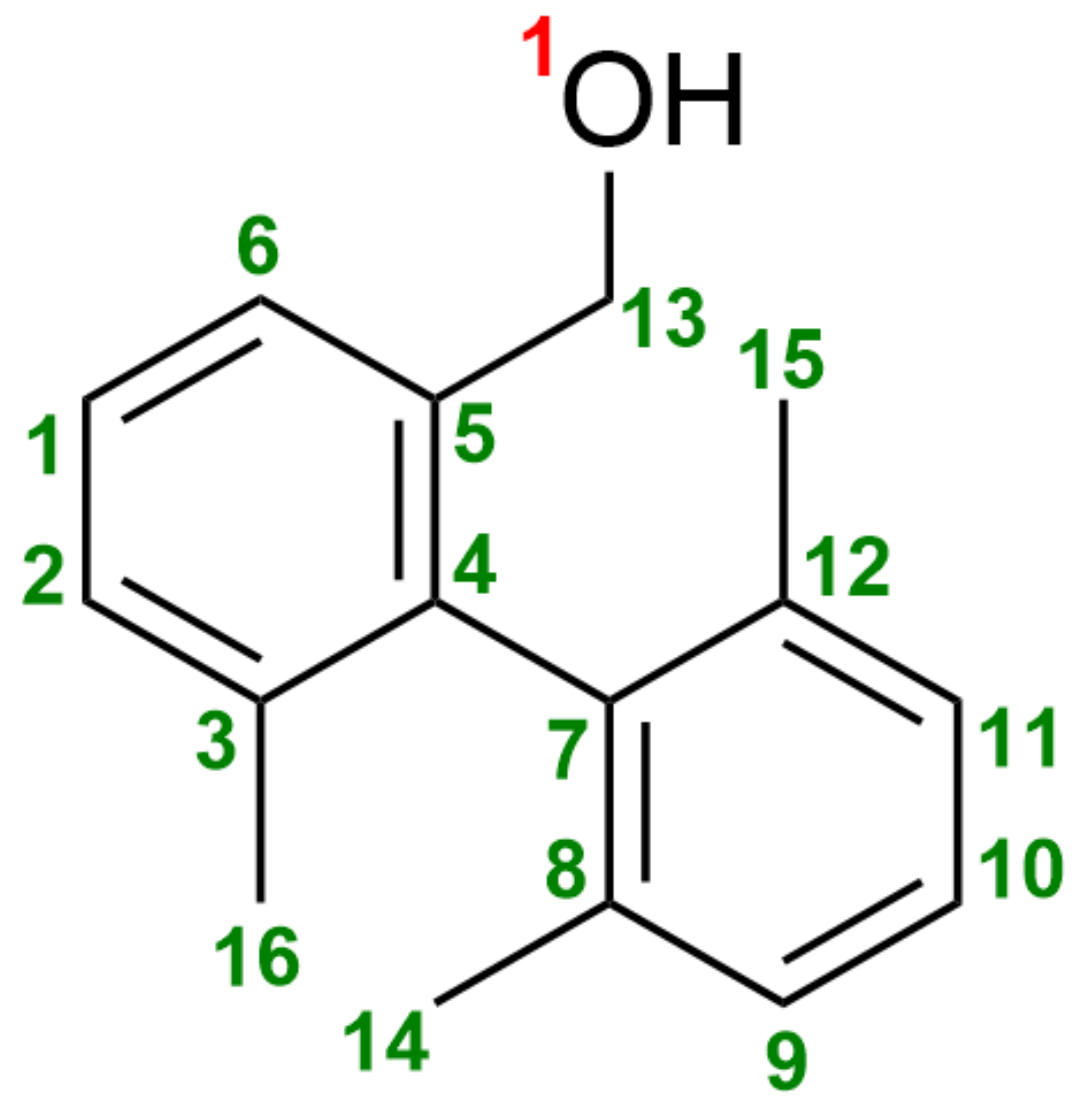

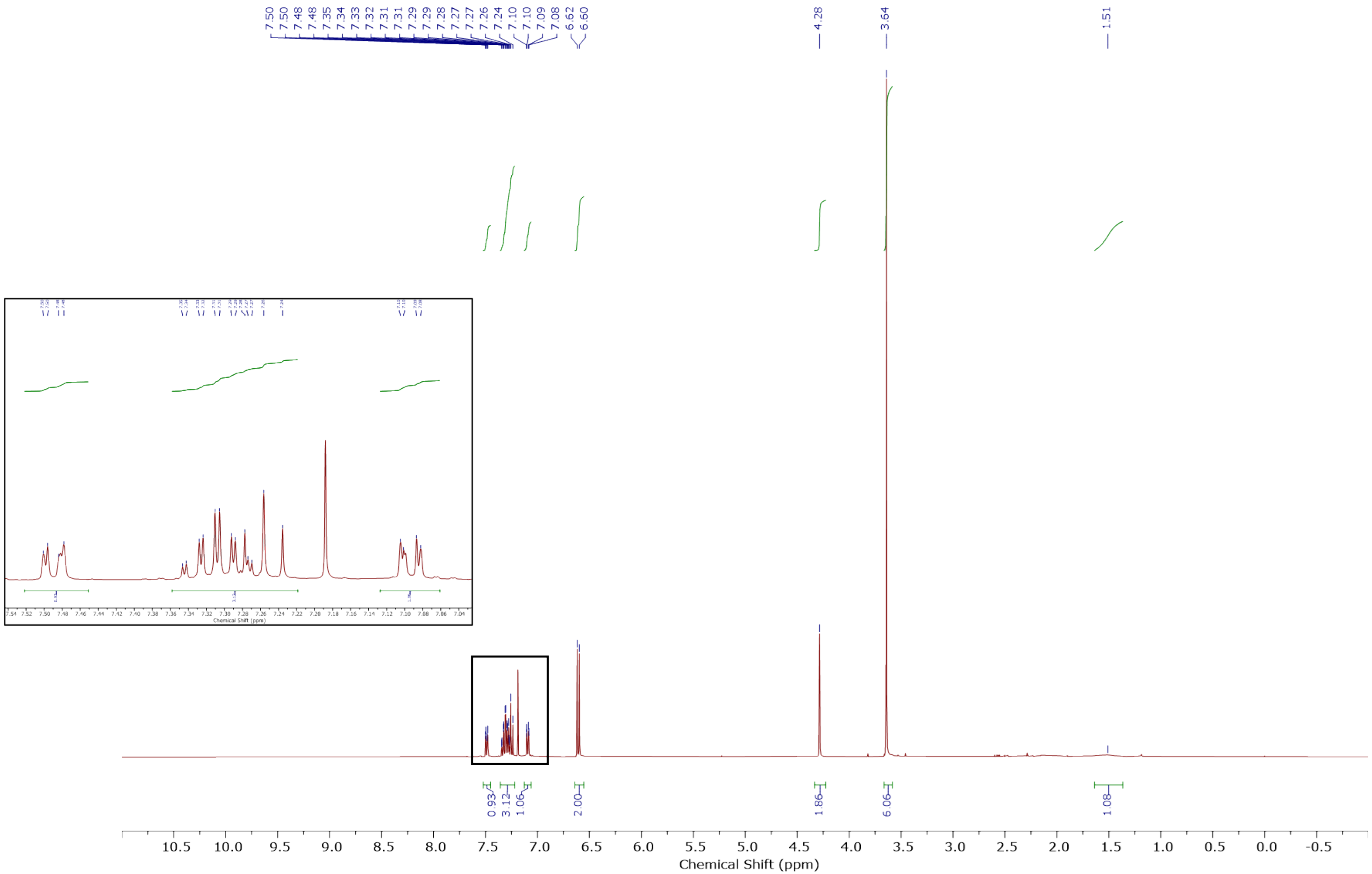

Figure S 24: ^{1}H-NMR spectrum of BBA-8,12-OMe (400 MHz, $CDCl_3$).

Table S 22: Tabulated ^{1}H-NMR spectrum of BBA-8,12-OMe (400 MHz, $CDCl_3$).

Peak Shift / ppm	Normalised Integration	Observed Splitting Pattern	Chemical Group	Atoms Assigned
1.50	1.08	Broad *Singlet*	-OH	H1A
3.62	6.06	*Singlet*	$-CH_3$	H14A – 14C<br>H15A – 15C
4.28	1.86	*Singlet*	$-CH_2$	H13A – 13B
6.59	2.00	*Doublet*	Aromatic -CH	H9 + H11
7.10	1.06	*dd*	Aromatic -CH	H10
7.29	3.12	*Multiplet*	Aromatic -CH	H1 / H2 / H3 / H6
7.49	0.93	*dd*	Aromatic -CH	H1 / H2 / H3 / H6

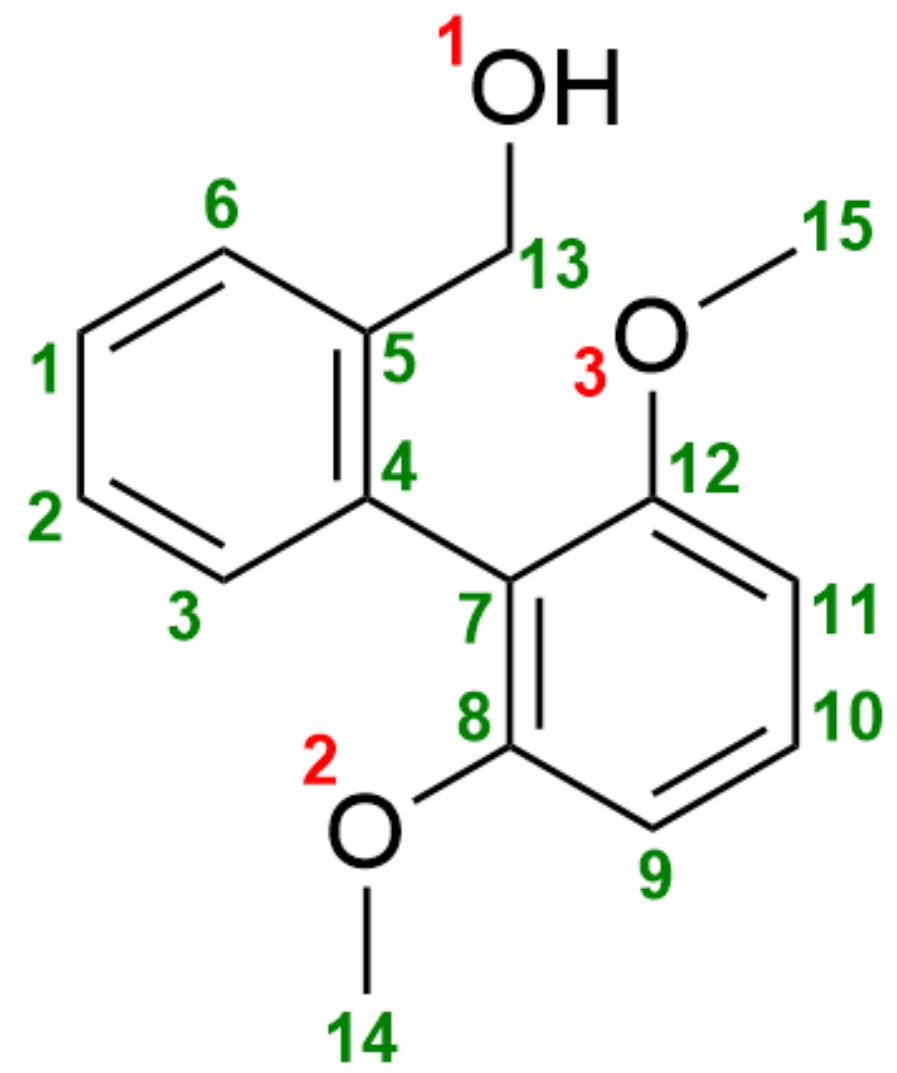

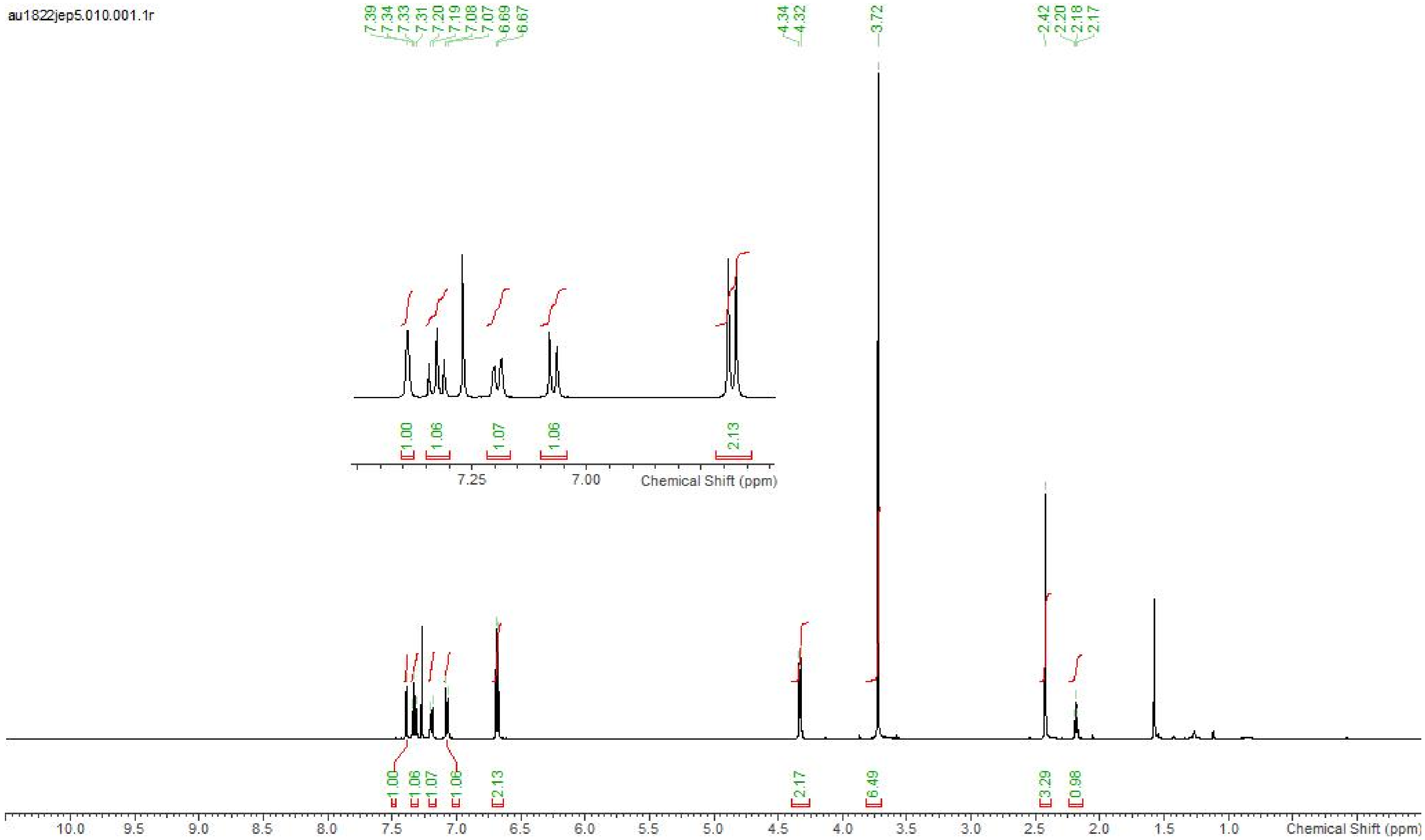

Figure S 25: ^{1}H-NMR spectrum of BBA-(1-Me)-8,12-OMe (400 MHz, $CDCl_3$).

Table S 23: Tabulated ^{1}H-NMR spectrum of BBA-(1-Me)-8,12-OMe (400 MHz, $CDCl_3$).

Peak Shift / ppm	Normalised Integration	Observed Splitting Pattern	Chemical Group	Atoms Assigned
2.18	0.98	*Triplet*	-OH	H1A
2.42	3.29	*Singlet*	$-CH_3$	H16A – 16C
3.72	6.49	*Singlet*	$-OCH_3$	H14A – 14C<br>H15A – 15C
4.33	2.17	*Doublet*	$-CH_2$	H13A – 13B
6.68	2.13	*Doublet*	Aromatic -CH	H9 + H11
7.07	1.06	*Doublet*	Aromatic -CH	H1 / H2
7.19	1.07	*Doublet*	Aromatic -CH	H1 / H2
7.33	1.06	*Triplet*	Aromatic -CH	H10
7.39	1.00	*Singlet*	Aromatic -CH	H6

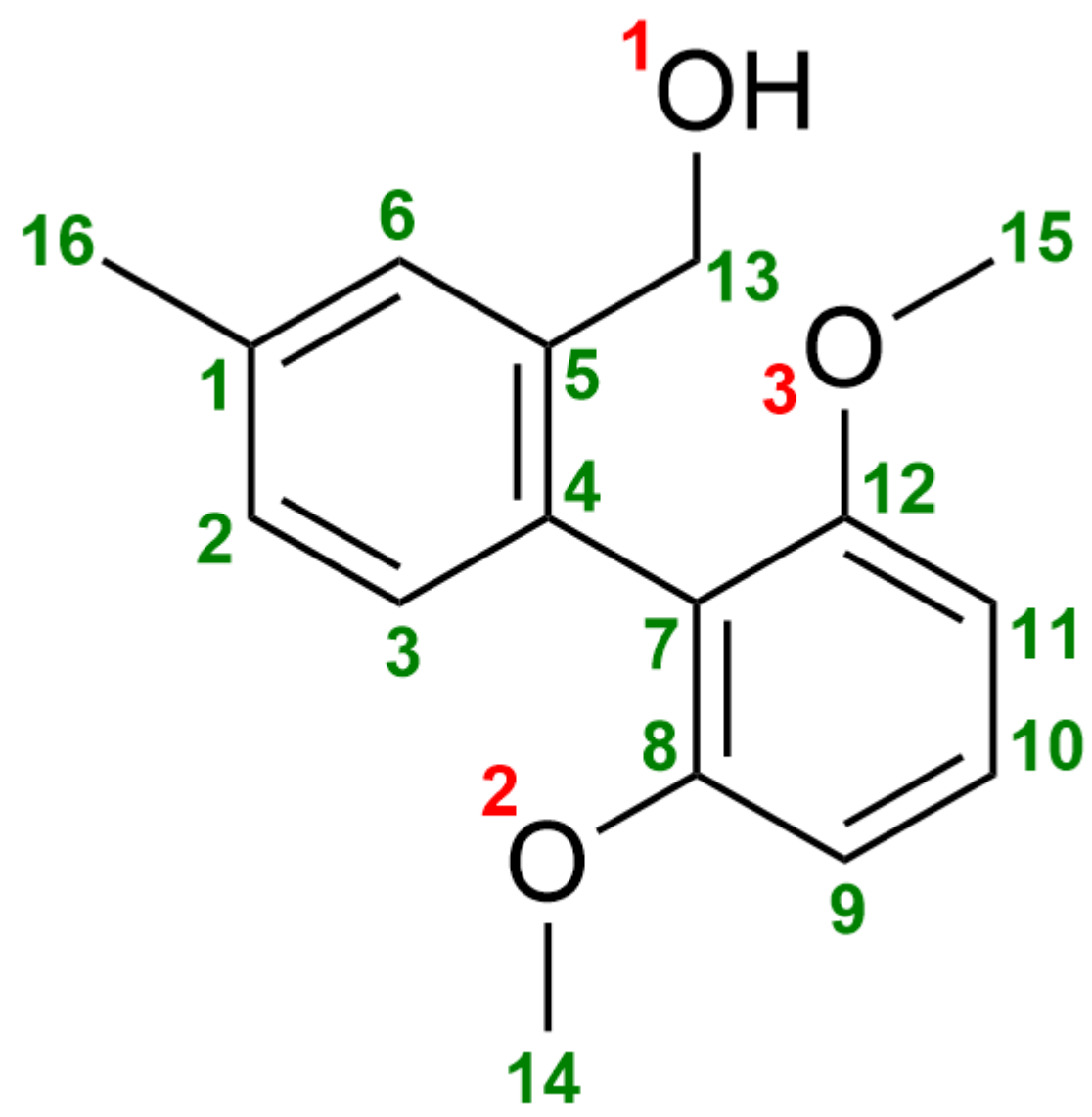

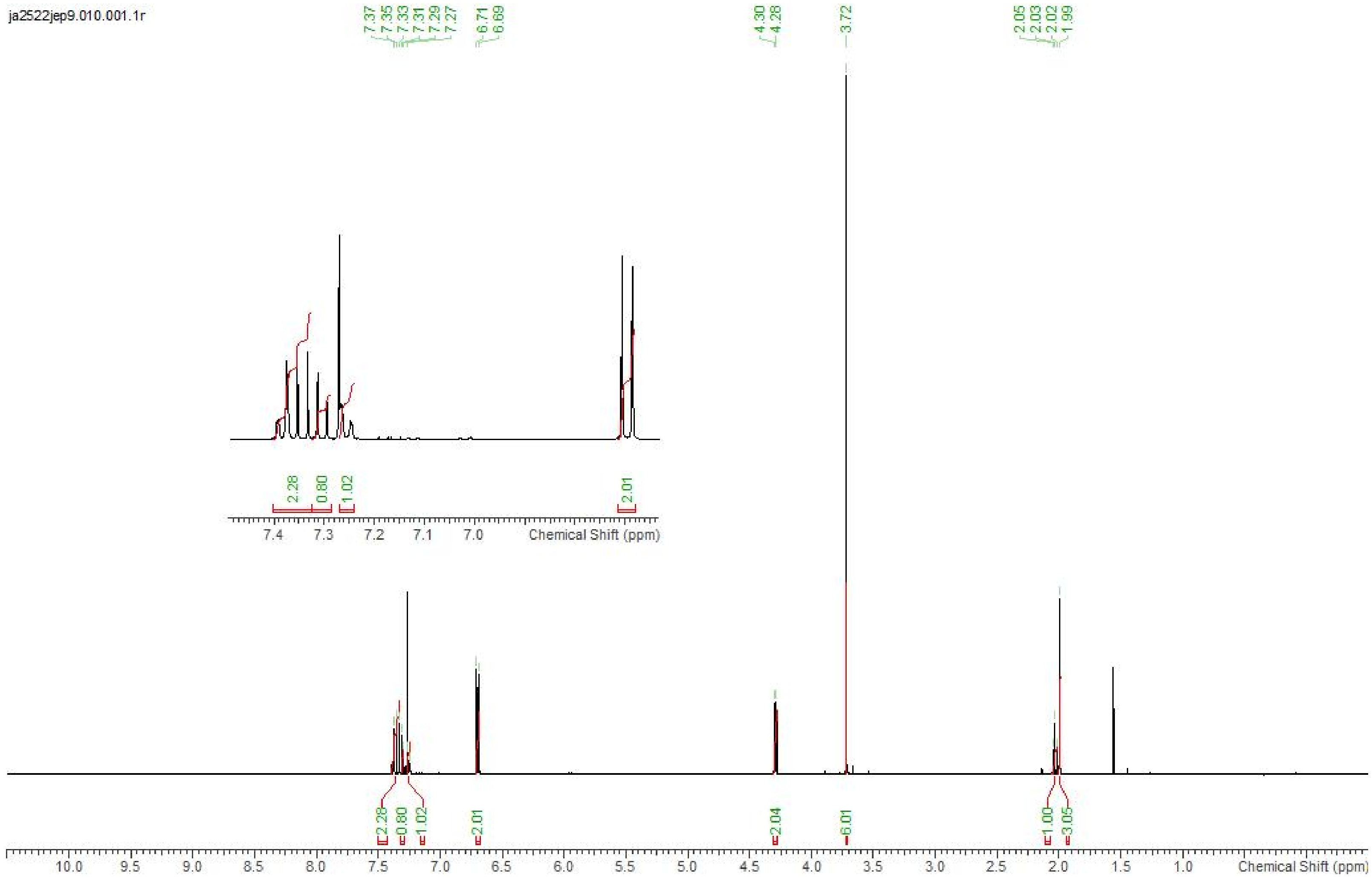

Figure S 26: ^{1}H-NMR spectrum of BBA-(3-Me)-8,12-OMe (400 MHz, $CDCl_3$).

Table S 24: Tabulated ^{1}H-NMR spectrum of BBA-(3-Me)-8,12-OMe (400 MHz, $CDCl_3$).

Peak Shift / ppm	Normalised Integration	Observed Splitting Pattern	Chemical Group	Atoms Assigned
1.99	3.05	*Singlet*	$-CH_3$	H16A – 16C
2.03	1.00	*Triplet*	-OH	H1A
3.72	6.01	*Singlet*	$-OCH_3$	H14A – 14C<br>H15A – 15C
4.29	2.04	*Doublet*	$-CH_2$	H13A – 13B
6.70	2.01	*Doublet*	Aromatic -CH	H9 + H11
7.26	1.02	*Multiplet*	Aromatic -CH	H10
7.31	0.80	*Apparent t*	Aromatic -CH	H1 / H2 / H6
7.35	2.28	*Triplet*	Aromatic -CH	H1 / H2 / H6

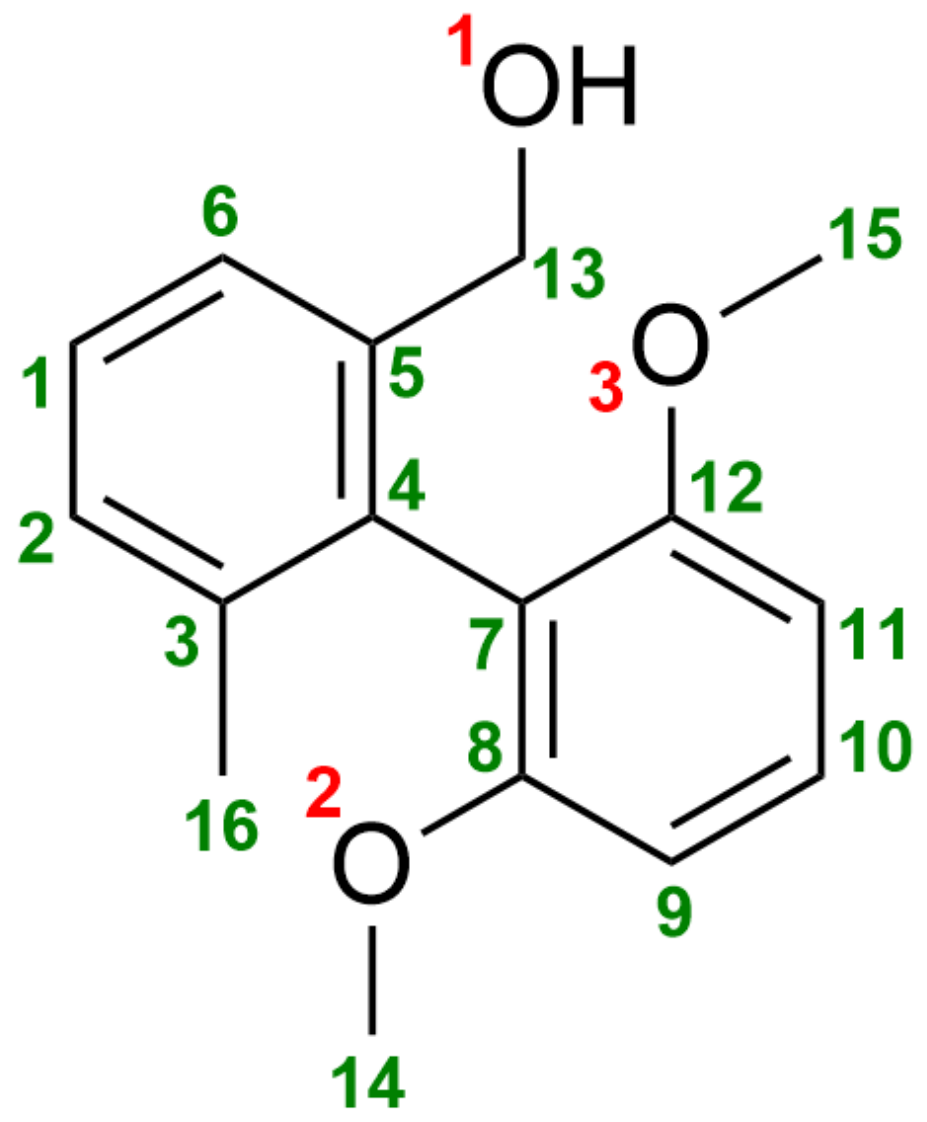

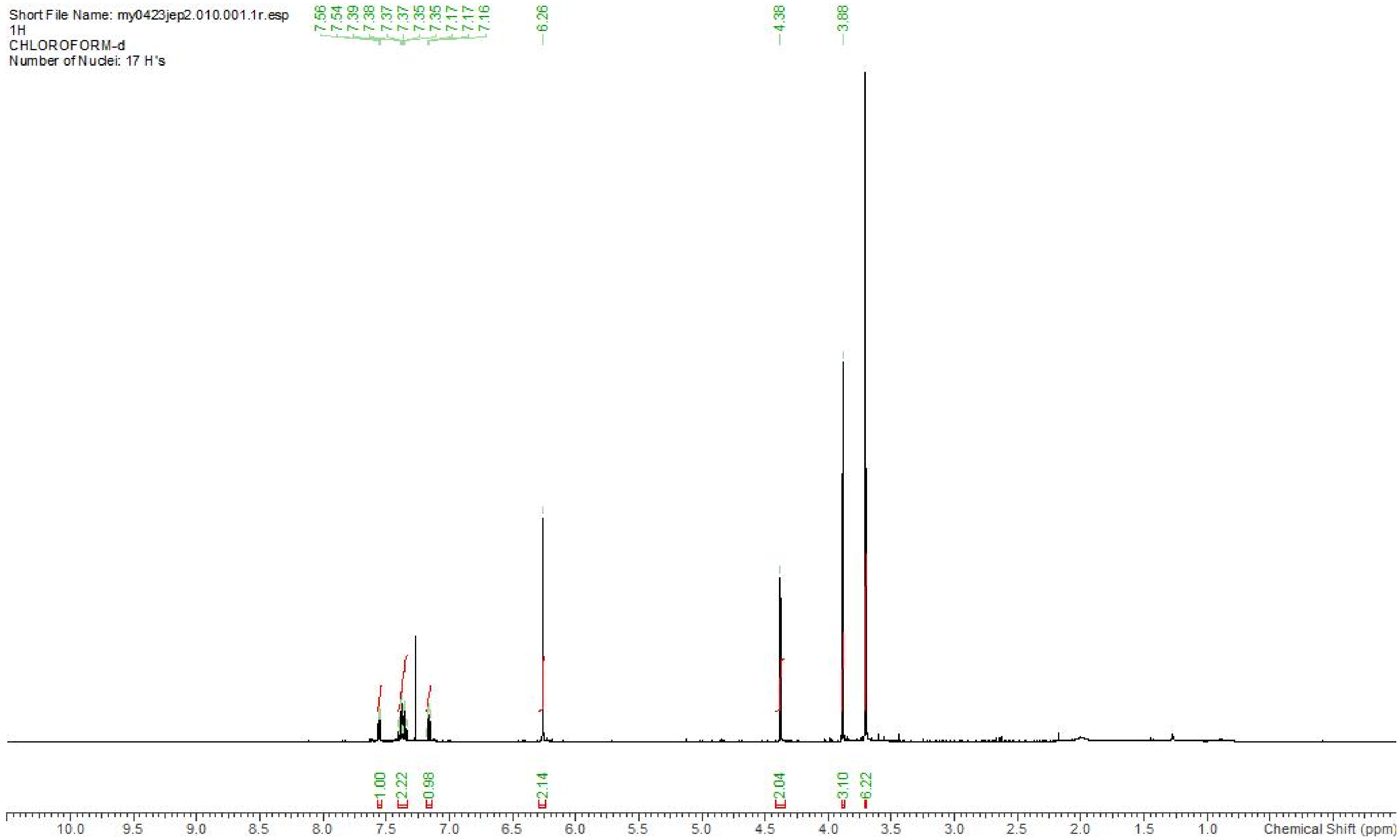

Figure S 27: ^{1}H-NMR spectrum of BBA-8,10,12-OMe at 298K (400 MHz, $CDCl_3$).

Table S 25: Tabulated ^{1}H-NMR spectrum of BBA-8,10,12-OMe (500 MHz, $CDCl_3$).

Peak Shift / ppm	Normalised Integration	Observed Splitting Pattern	Chemical Group	Atoms Assigned
3.69 – 3.71	6.22	*Singlet*	$-OCH_3$	H14A – 14C<br>H15A – 15C
3.88	3.10	*Singlet*	$-OCH_3$	H16A – 16C
4.38	2.04	*Singlet*	$-CH_2$	H13A – 13B
6.26	2.14	*Singlet*	Aromatic -CH	H9 + H11
7.16	0.98	*dd*	Aromatic -CH	H1 / H2 / H3 / H6
7.37	2.22	*Multiplet*	Aromatic -CH	H1 / H2 / H3 / H6
7.55	1.00	*dd*	Aromatic -CH	H1 / H2 / H3 / H6

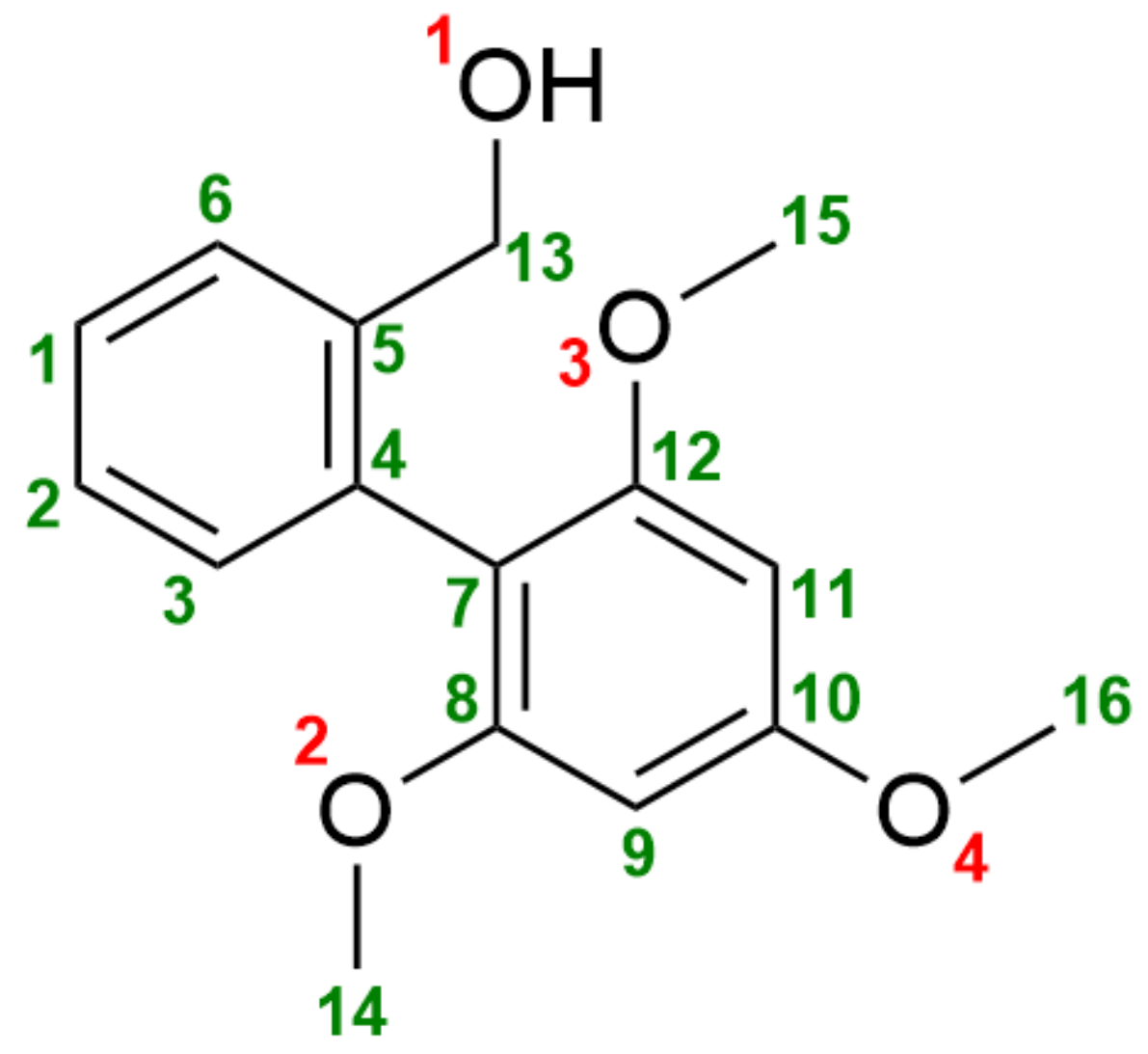

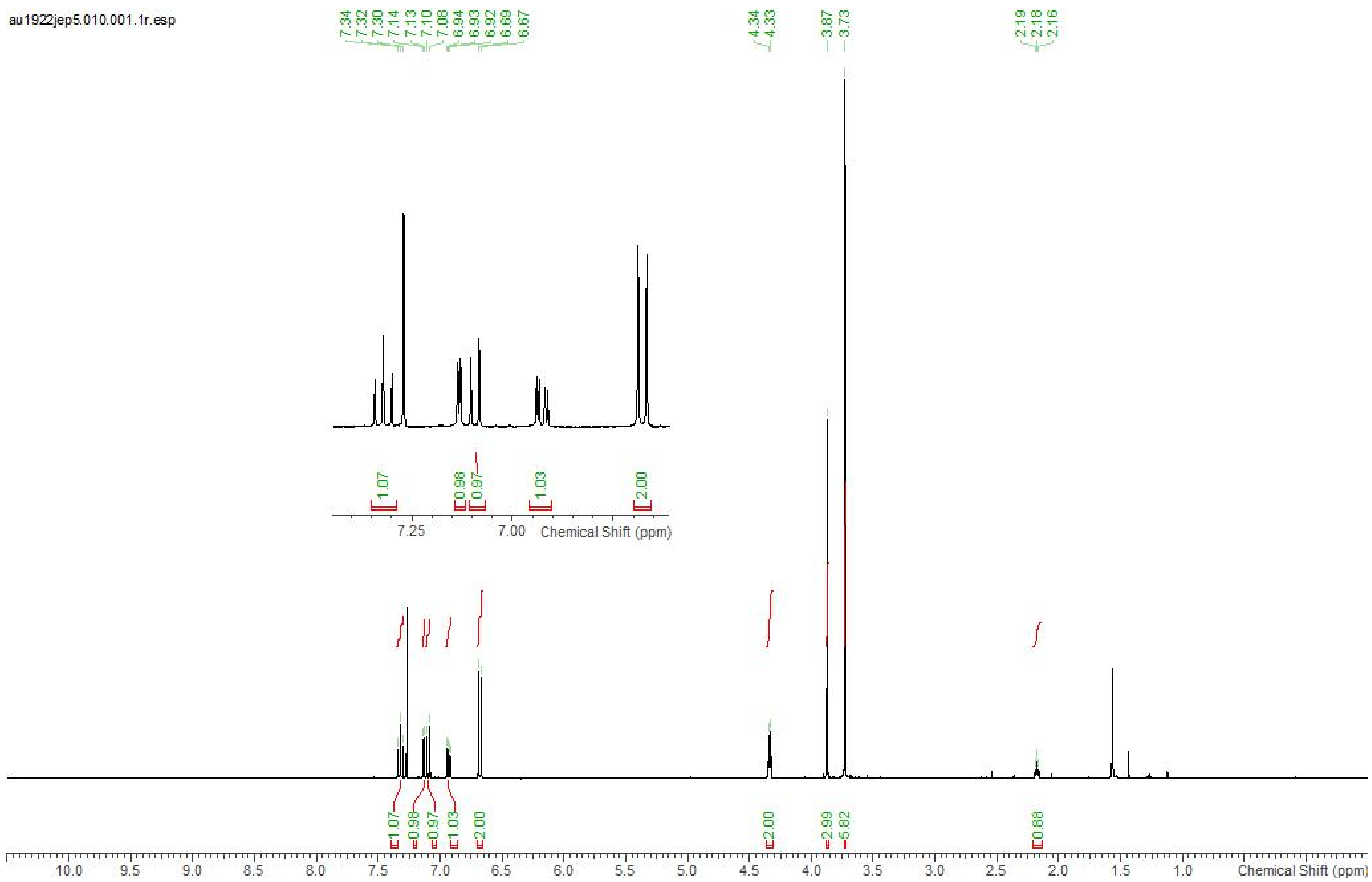

Figure S 28: ^{1}H-NMR spectrum of BBA-1,8,12-OMe (400 MHz, $CDCl_3$).

Table S 26: Tabulated ^{1}H-NMR spectrum of BBA-8,10,12-OMe (400 MHz, $CDCl_3$).

Peak Shift / ppm	Normalised Integration	Observed Splitting Pattern	Chemical Group	Atoms Assigned
2.18	0.88	*Triplet*	-OH	H1A
3.73	5.92	*Singlet*	$-OCH_3$	H14A – 14C<br>H15A – 15C
3.87	2.99	*Singlet*	$-OCH_3$	H16A – 16C
4.34	2.00	*Doublet*	$-CH_2$	H13A – 13B
6.68	2.00	*Doublet*	Aromatic -CH	H9 + H11
6.93	1.03	*dd*	Aromatic -CH	H2
7.09	0.97	*Doublet*	Aromatic -CH	H3 / H6
7.13	0.98	*Doublet*	Aromatic -CH	H3 / H6
7.32	1.07	*Triplet*	Aromatic -CH	H10

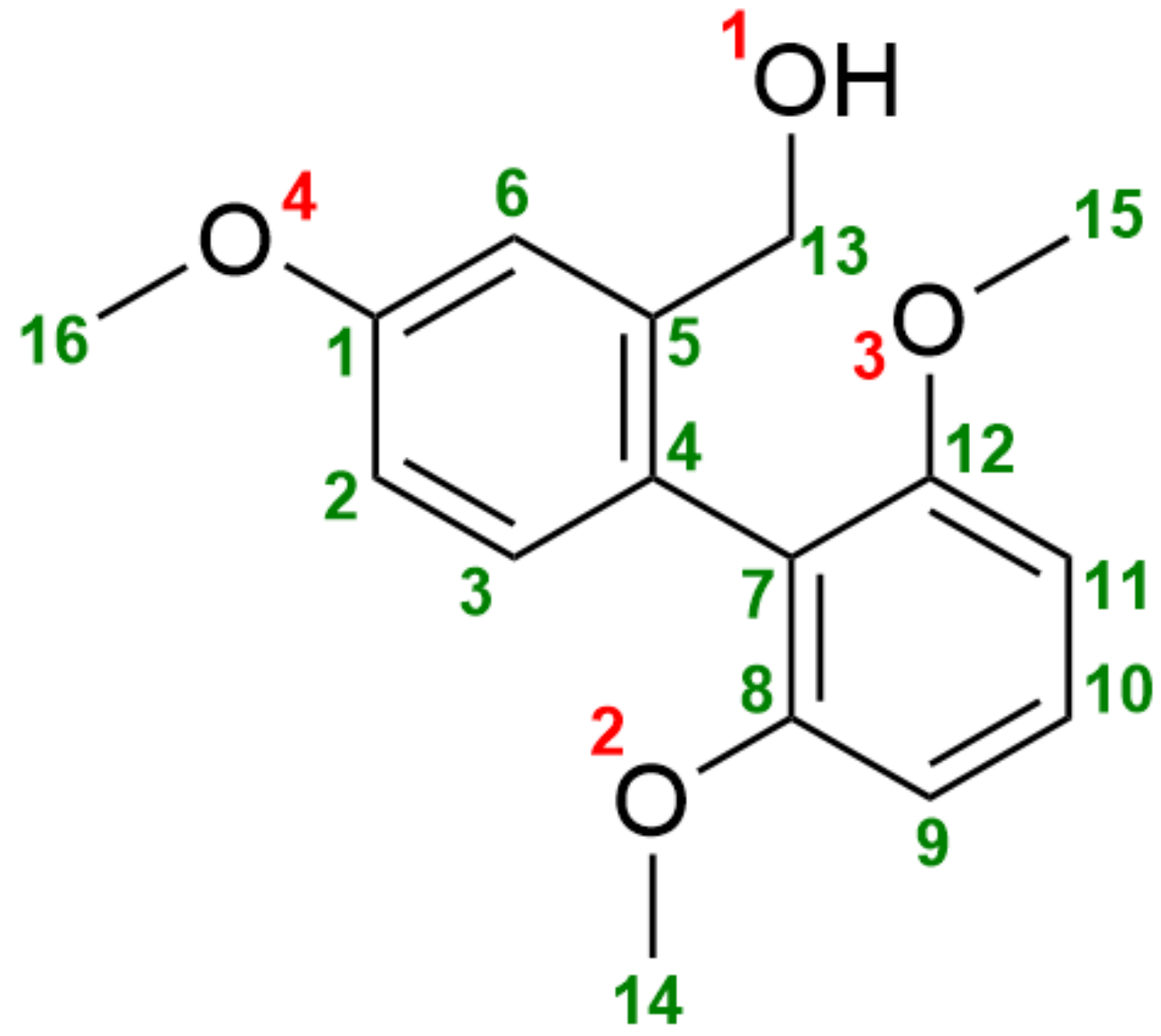

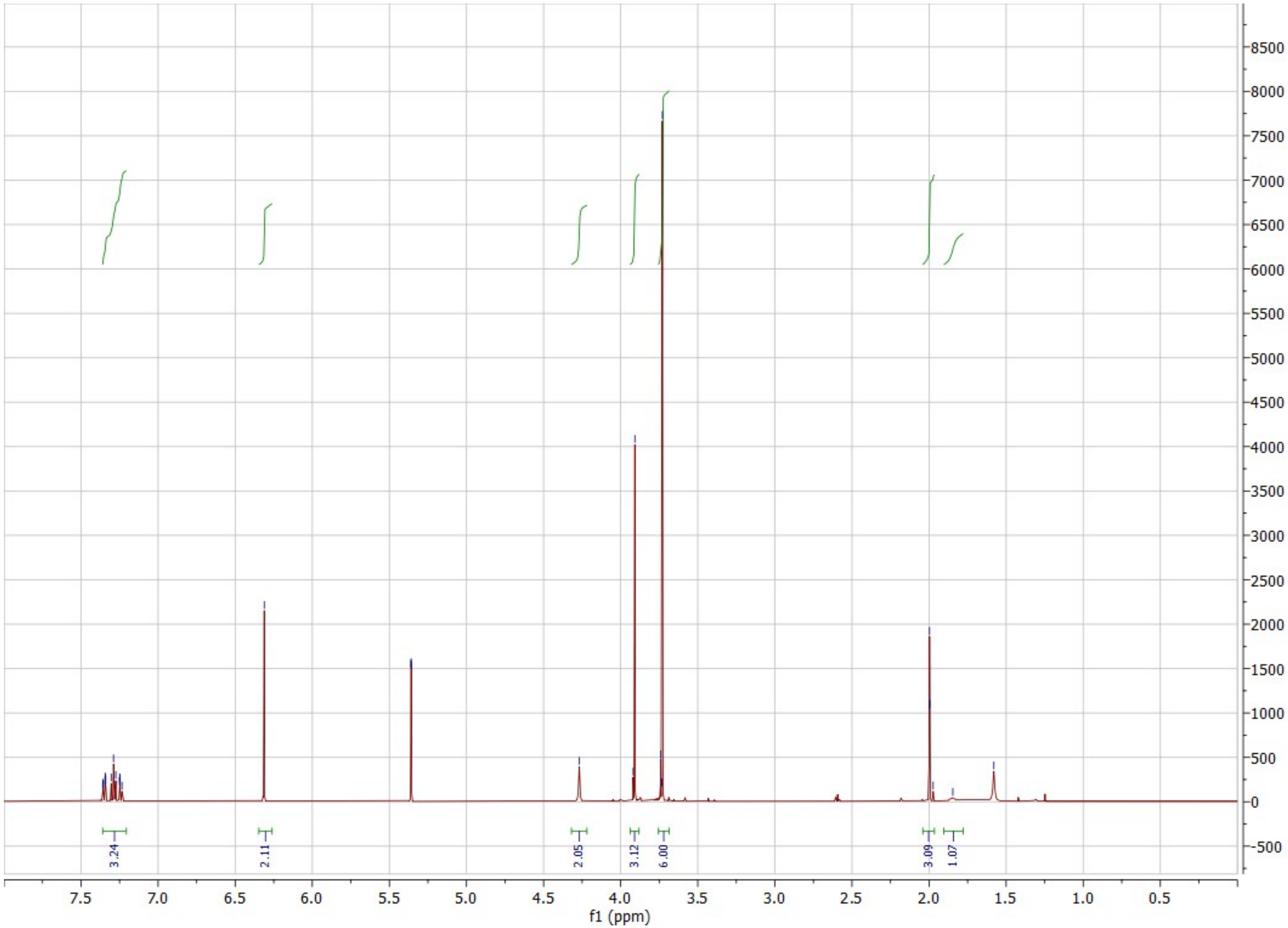

Figure S 29: ^{1}H-NMR spectrum of BBA-(3-Me)-8,10,12-OMe at 298K (500 MHz, CD_2Cl_2).

Table S 27: Tabulated ^{1}H-NMR spectrum of BBA-(3-Me)-8,10,12-OMe at 298K (500 MHz, CD_2Cl_2).

Peak Range / ppm	Normalised Integration	Observed Splitting Pattern	Chemical Group	Atoms Assigned
1.78 – 1.90	1.07	*Singlet*	-OH	H1A
1.96 – 2.04	3.09	*Singlet*	$-CH_3$	H16A – H16C
3.69 – 3.75	6.00*	*Singlet*	$-OCH_3$	H14A – H14C<br>H15A – H15C
3.86 – 3.95	3.12	*Singlet*	$-OCH_3$	H17A – H17C
4.22 – 4.32	2.05	*Singlet*	$-CH_2$	H13A – H13B
6.26 – 6.35	2.11	*Singlet*	Aromatic -CH	H9 + H11
7.20 – 7.38	3.24	*Multiplet*	Aromatic -CH	H1 + H2 + H6

*Reference peak for integration. Signals at 1.58 ppm and 5.36 ppm were caused by water and non-deuterated DCM respectively.

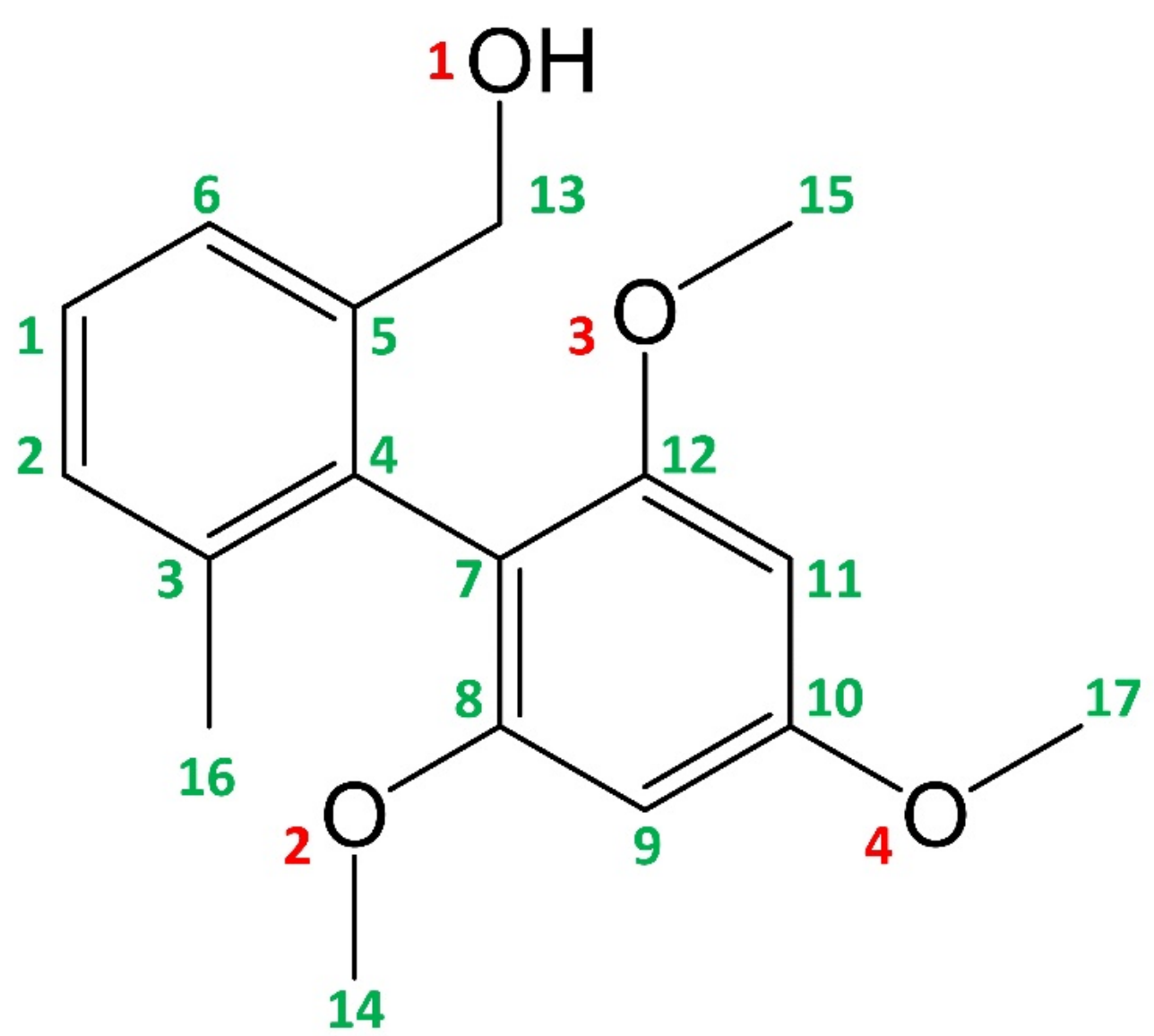

Figure S 30: BBA-(3-Me)-8,10,12-OMe molecular structure and atom numbering scheme.

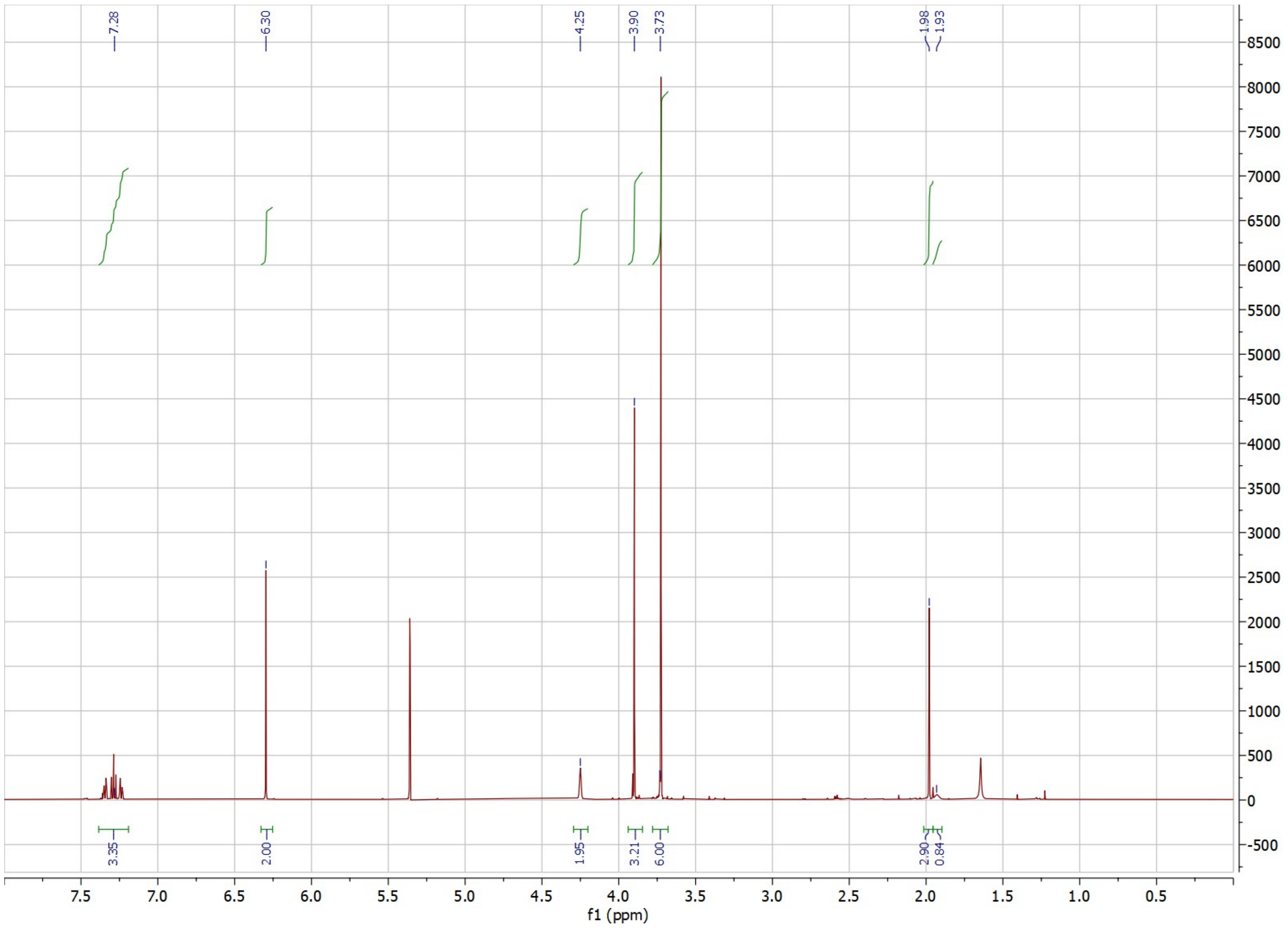

Figure S 31: ^{1}H-NMR spectrum of BBA-(3-Me)-8,10,12-OMe at 273K (500 MHz, CD_2Cl_2).

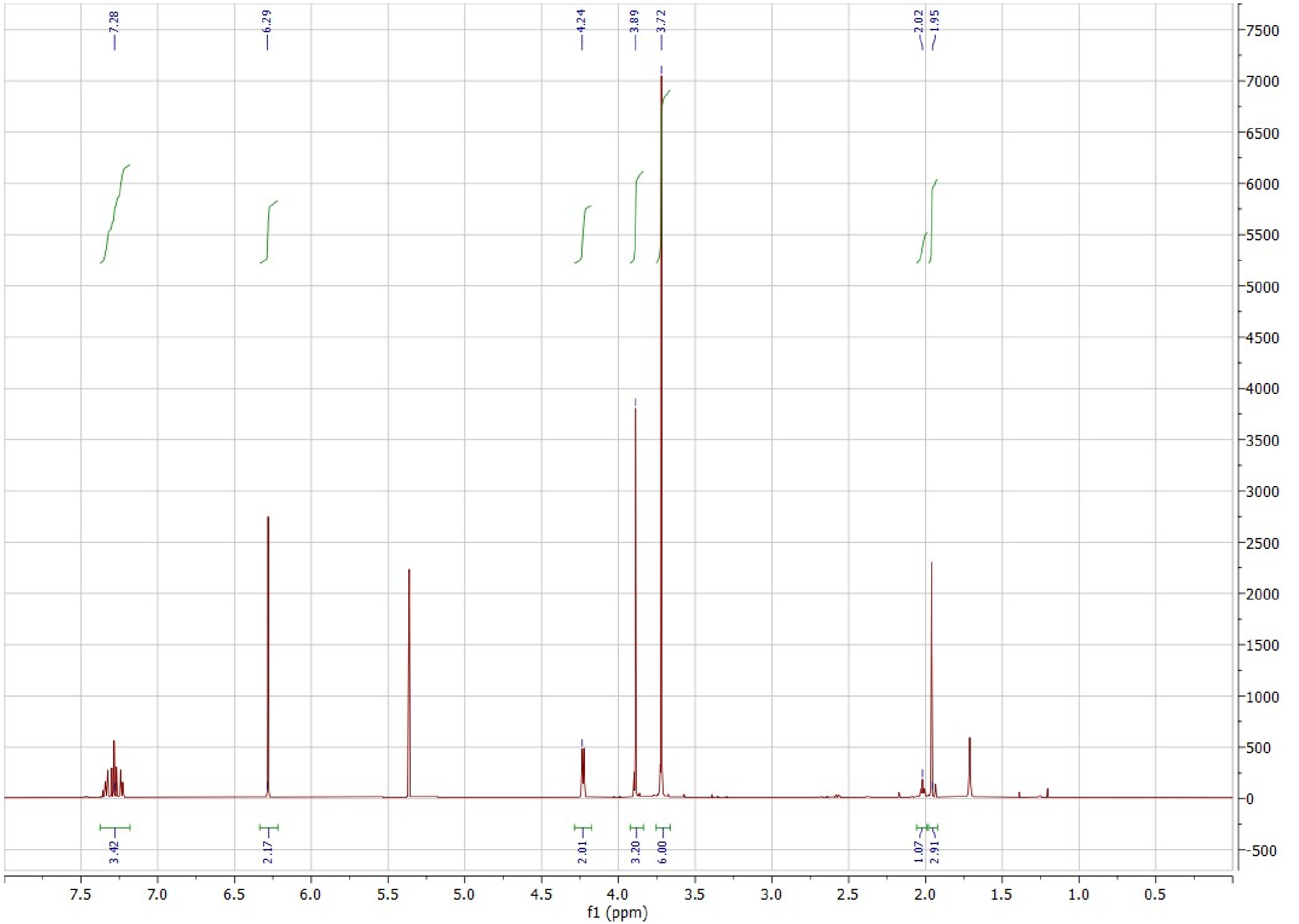

Figure S 32: ^{1}H-NMR spectrum of BBA-(3-Me)-8,10,12-OMe at 243K (500 MHz, CD_2Cl_2).

Table S 28: Tabulated ^{1}H-NMR spectrum of BBA-(3-Me)-8,10,12-OMe at 273K (500 MHz, CD_2Cl_2).

Peak Range / ppm	Normalised Integration	Observed Splitting Pattern	Chemical Group	Atoms Assigned
1.90 – 1.95	0.84	*Singlet*	-OH	H1AA
1.95 – 2.02	2.90	*Singlet*	$-CH_3$	H16A – H16C
3.68 – 3.78	6.00*	*Singlet*	$-OCH_3$	H14A – H14C<br>H15A – H15C
3.85 – 3.94	3.21	*Singlet*	$-OCH_3$	H17A – H17C
4.20 – 4.29	1.95	*Singlet*	$-CH_2$	H13A – H13B
6.25 – 6.33	2.00	*Singlet*	Aromatic -CH	H9 + H11
7.19 – 7.38	3.35	*Multiplet*	Aromatic -CH	H1 + H2 + H6

* Reference peak for integration.

Table S 29: Tabulated ^{1}H-NMR spectrum of BBA-(3-Me)-8,10,12-OMe at 243K (500 MHz, CD_2Cl_2).

Peak Range / ppm	Normalised Integration	Observed Splitting Pattern	Chemical Group	Atoms Assigned
1.92 – 1.98	2.91	*Singlet*	$-CH_3$	H16A – H16C
1.99 – 2.06	1.07	*Singlet*	-OH	H1AA
3.66 – 3.75	6.00*	*Singlet*	$-OCH_3$	H14A – H14C<br>H15A – H15C
3.84 – 3.92	3.20	*Singlet*	$-OCH_3$	H17A – H17C
4.18 – 4.29	2.01	*Singlet*	$-CH_2$	H13A – H13B
6.22 – 6.34	2.17	*Singlet*	Aromatic -CH	H9 + H11
7.18 – 7.37	3.42	*Multiplet*	Aromatic -CH	H1 + H2 + H6

* Reference peak for integration

9. Guest Flexibility and Intramolecular Energies

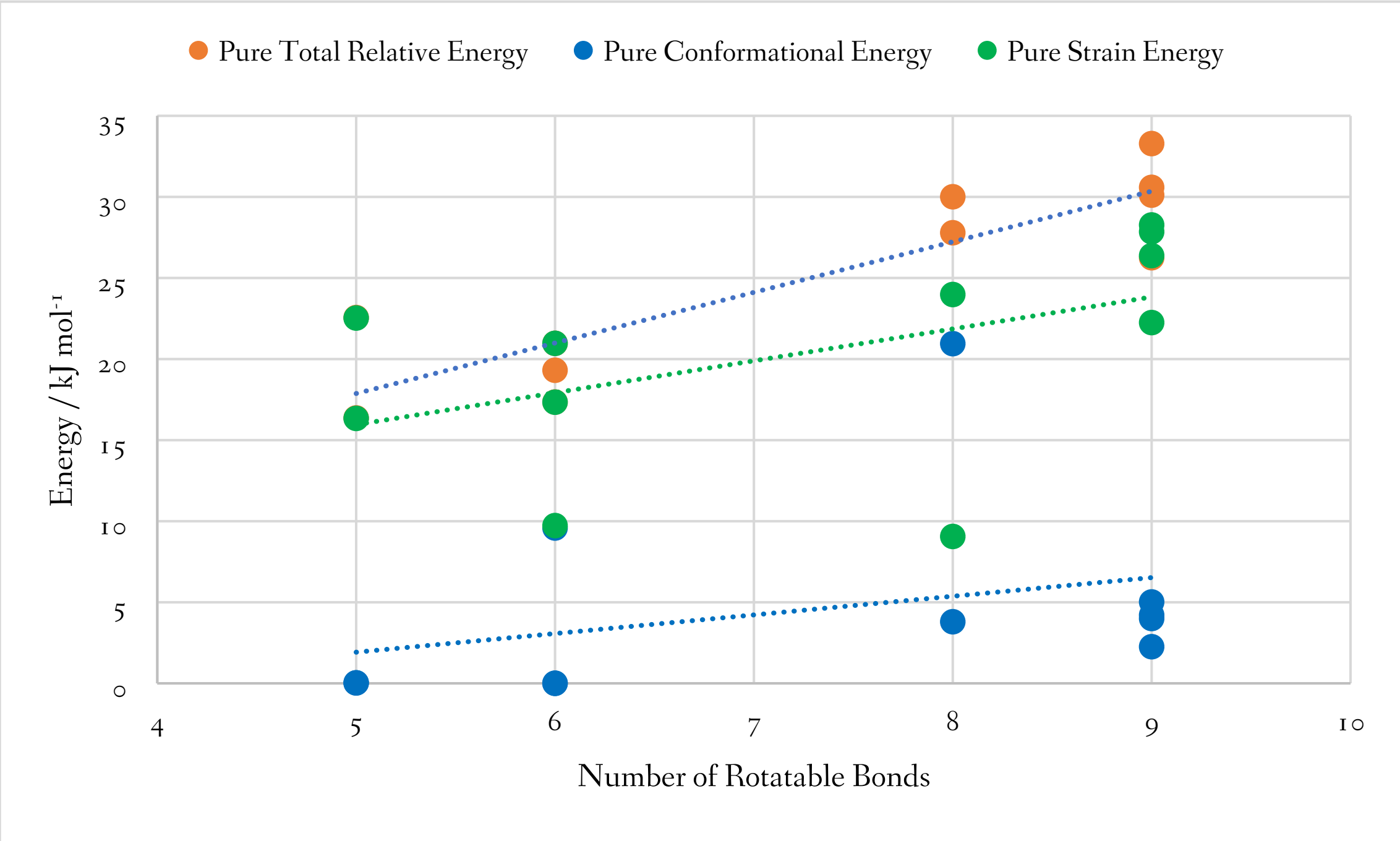

Figure S 33: Intramolecular energy components plotted as a function of guest rotatable bonds for conventional SCXRD structures (methyl included).

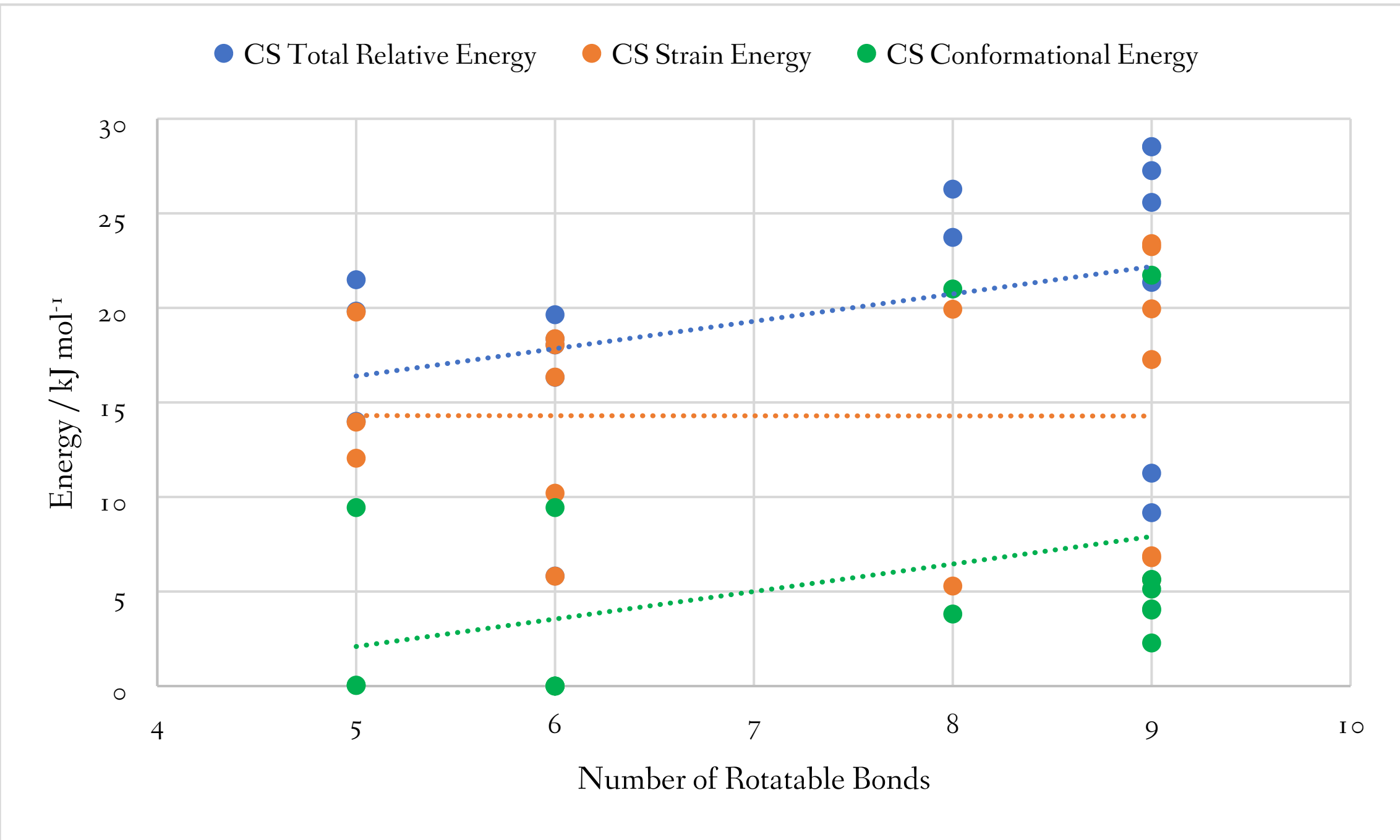

Figure S 34: Intramolecular energy components plotted as a function of guest rotatable bonds for crystalline sponge structures (methyl included).

Table S 30: Linear regression statistics for intramolecular energy components plotted as a function of guest rotatable bonds for conventional SCXRD and structures (methyl included).

		R^2-value	Standard Error	t-stat	P-value
Conventional SCXRD	$\square E_{total}$	0.8040	0.5084	6.4042	0.00008
	$\square E_{strain}$	0.3016	1.0704	2.0781	0.06441
	$\square E_{conf}$	0.0859	1.0641	0.9696	0.35511
Crystalline Sponge	$\square E_{total}$	0.1297	0.9685	1.4952	0.15560
	$\square E_{strain}$	0.0000	0.9845	0.0059	0.99540
	$\square E_{conf}$	0.1314	0.9655	1.5062	0.15277

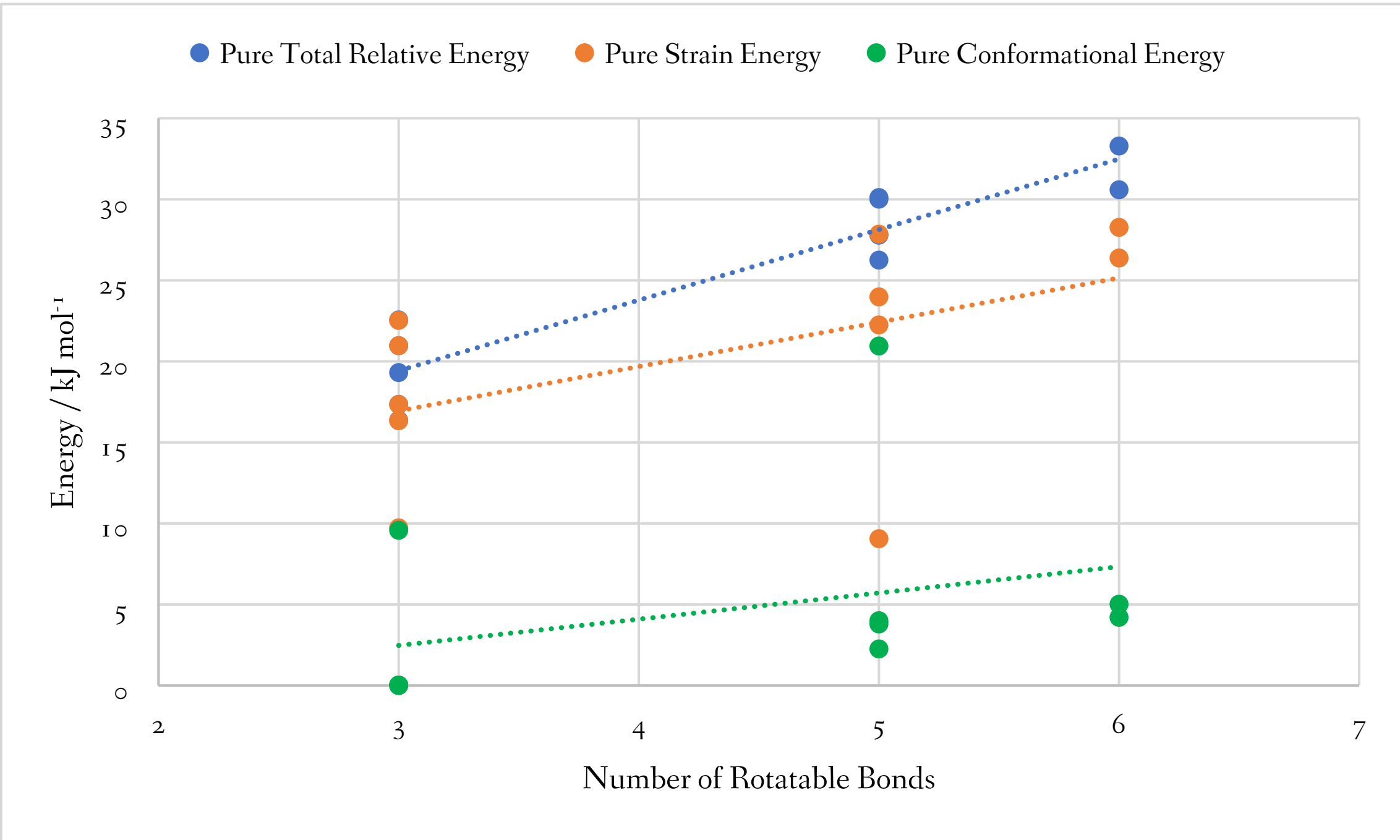

Figure S 35: Intramolecular energy components plotted as a function of guest rotatable bonds for conventional SCXRD structures (methyl not included).

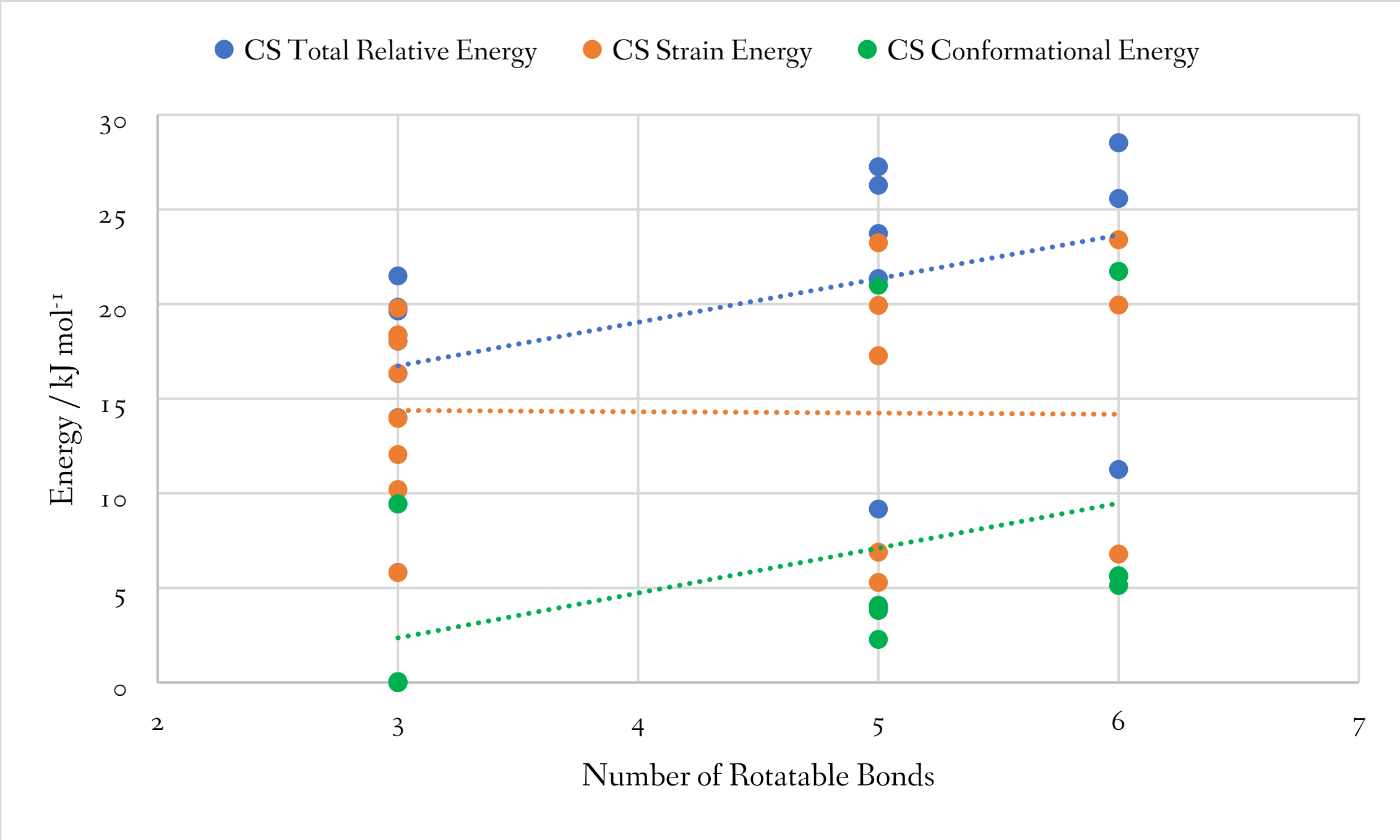

Figure S 36: Intramolecular energy components plotted as a function of guest rotatable bonds for conventional SCXRD structures (methyl not included).

Table S 31: Linear regression statistics for intramolecular energy components plotted as a function of guest rotatable bonds for conventional SCXRD and structures (methyl not included).

		R^2-value	Standard Error	t-stat	P-value
Conventional SCXRD	∆E_{total}	0.8984	0.4680	9.4019	0.000003
	∆E_{strain}	0.3451	1.3251	2.2954	0.044603
	□E_{conf}	0.0912	1.3565	1.0015	0.340201
Crystalline Sponge	∆E_{total}	0.1988	1.1951	1.9293	0.072831
	∆E_{strain}	0.0002	1.2659	0.0537	0.957849
	□E_{conf}	0.2117	1.1828	2.0069	0.063134

10. Guest Flexibility and Intermolecular Energies

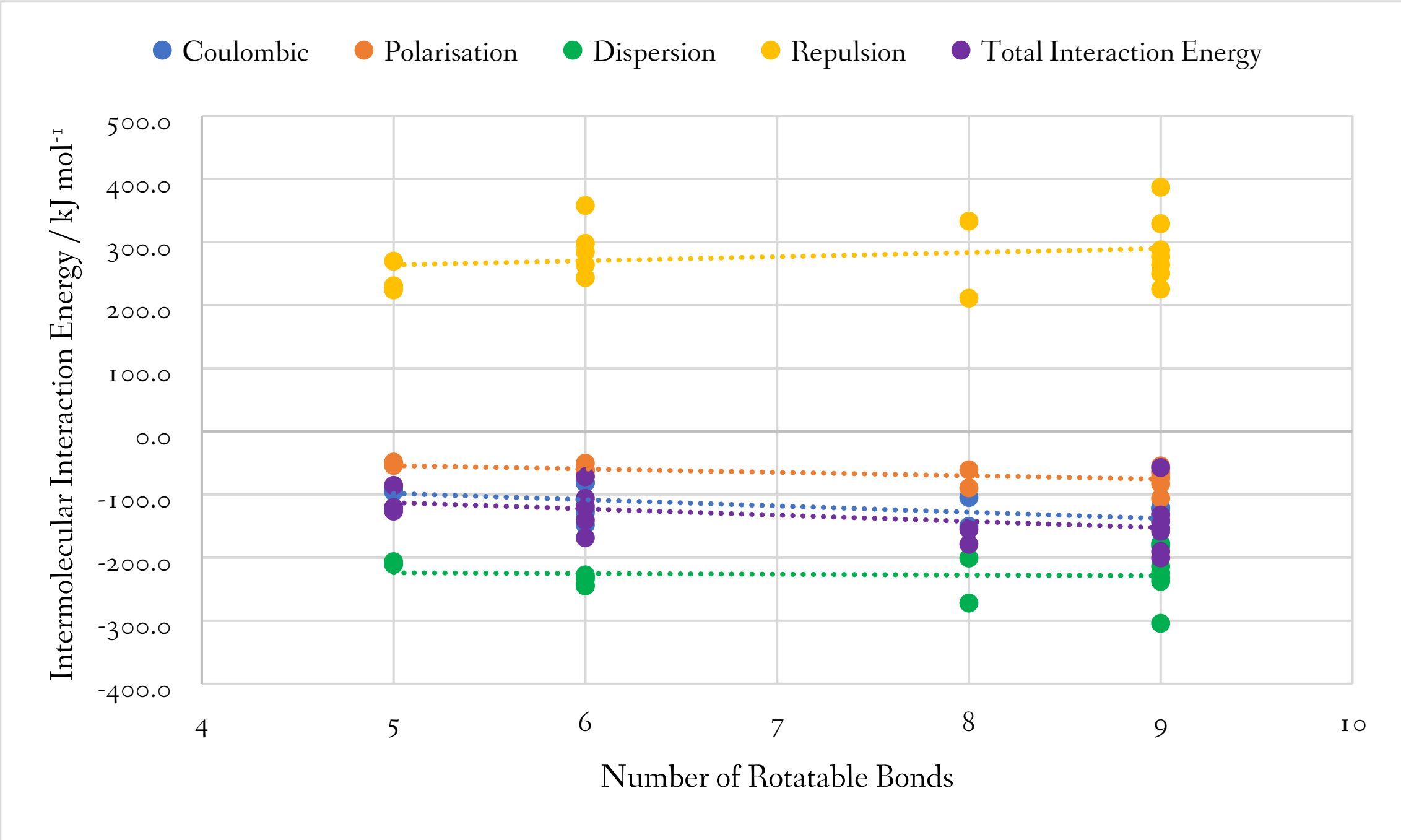

Figure S 37: Scatterplot of intermolecular interaction energies versus number of guest rotatable bonds for crystalline sponge structures (methyl included).

Table S 32: Linear regression statistics for scatterplot of intermolecular interaction energies versus number of guest rotatable bonds (methyl included).

	R^2-value	Standard Error	t-stat	P-value
E_{COU}	0.3780	3.2793	-3.0194	0.0086
E_{POL}	0.3475	1.8802	-2.8261	0.0128
E_{DISP}	0.0042	4.6704	-0.2503	0.8057
E_{REP}	0.0487	7.3872	0.8763	0.3947
E_{TOT}	0.1746	5.5635	-1.7814	0.0951

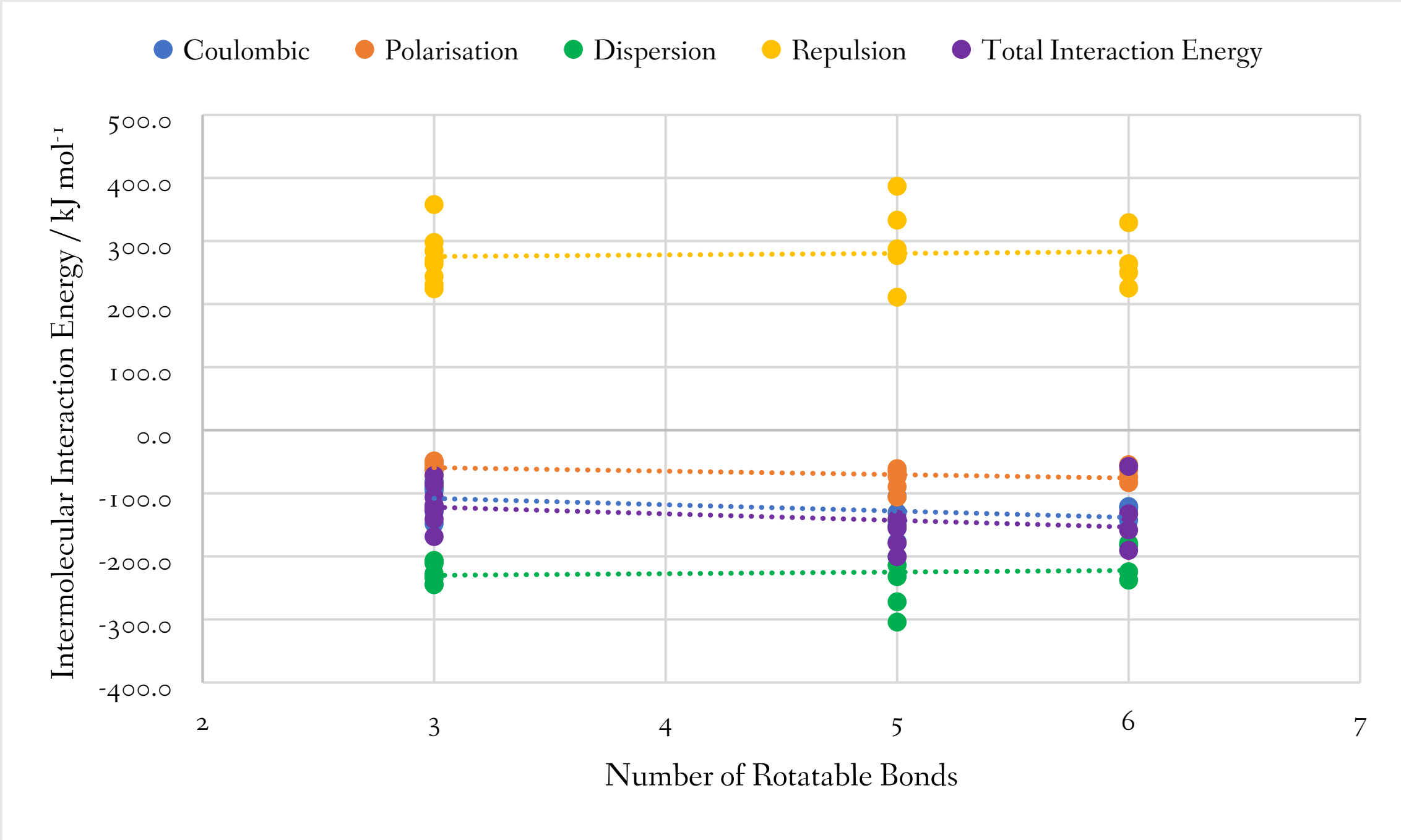

Figure S 38: Scatterplot of intermolecular interaction energies versus number of guest rotatable bonds for crystalline sponge structures (methyl not included).

Table S 33: Linear regression statistics for scatterplot of intermolecular interaction energies versus number of guest rotatable bonds (methyl not included).

	R^2-value	Standard Error	t-stat	P-value
E_{COU}	0.2357	4.6748	-2.1509	0.0482
E_{POL}	0.2245	2.6359	-2.0841	0.0547
E_{DISP}	0.0122	5.9819	0.4310	0.6726
E_{REP}	0.0043	9.7193	0.2543	0.8027
E_{TOT}	0.1185	7.3941	-1.4199	0.1761

11. Intramolecular and Intermolecular Energy Plots

11.1. Conventional SCXRD

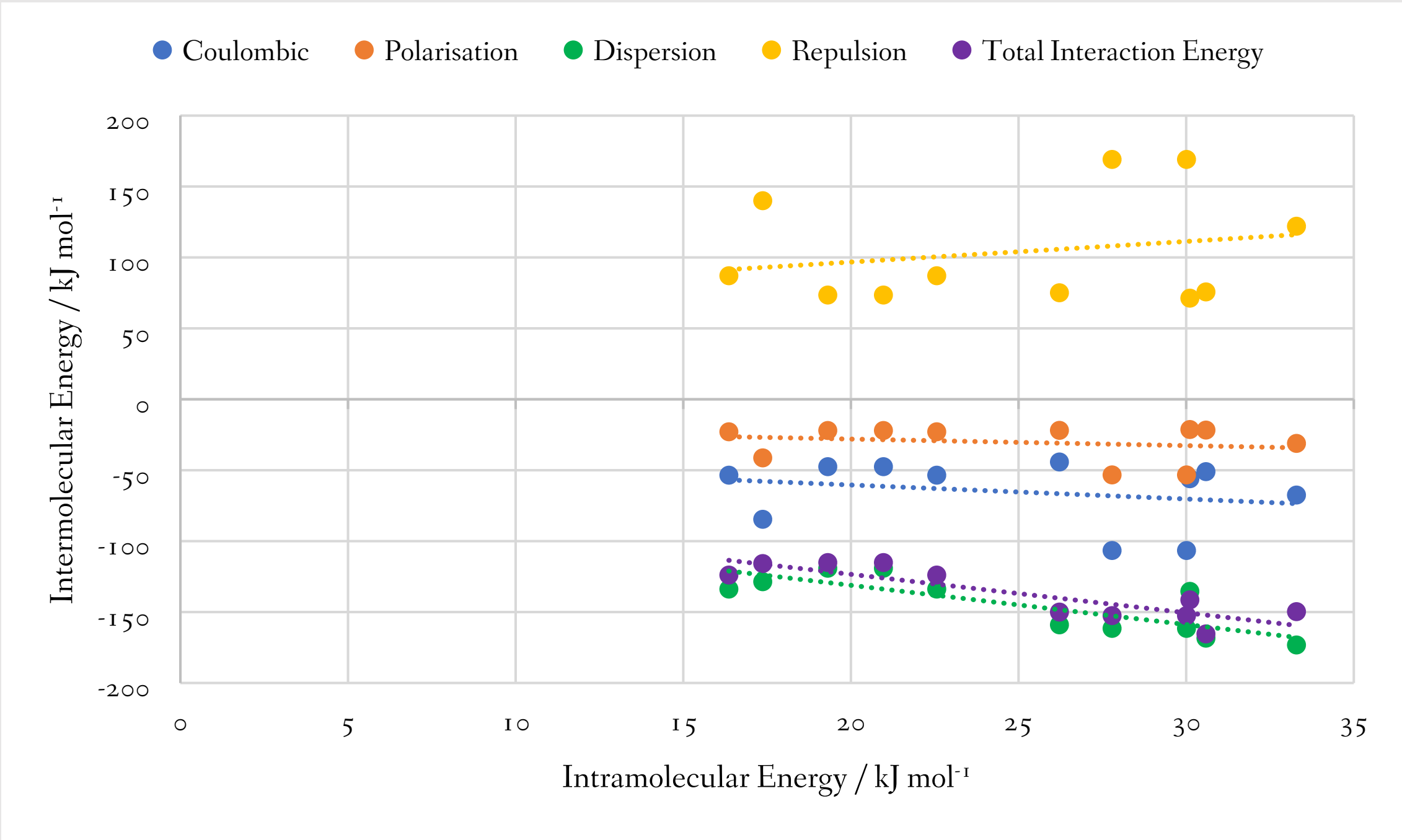

Figure S 39: Scatterplot of conventional SCXRD intermolecular interaction energies versus total relative energy.

Table S 34: Linear regression statistics for scatterplot of conventional SCXRD intermolecular interaction energies versus total relative energy (∆E_{total}).

	R^2-value	Standard Error	t-stat	P-value
E_{COU}	0.0620	1.2759	-0.7713	0.4603
E_{POL}	0.0445	0.7099	-0.6472	0.5337
E_{DISP}	0.6598	0.6621	-4.1780	0.0024
E_{REP}	0.0478	2.1517	0.6725	0.5181
E_{TOT}	0.7550	0.5147	-5.2670	0.0005

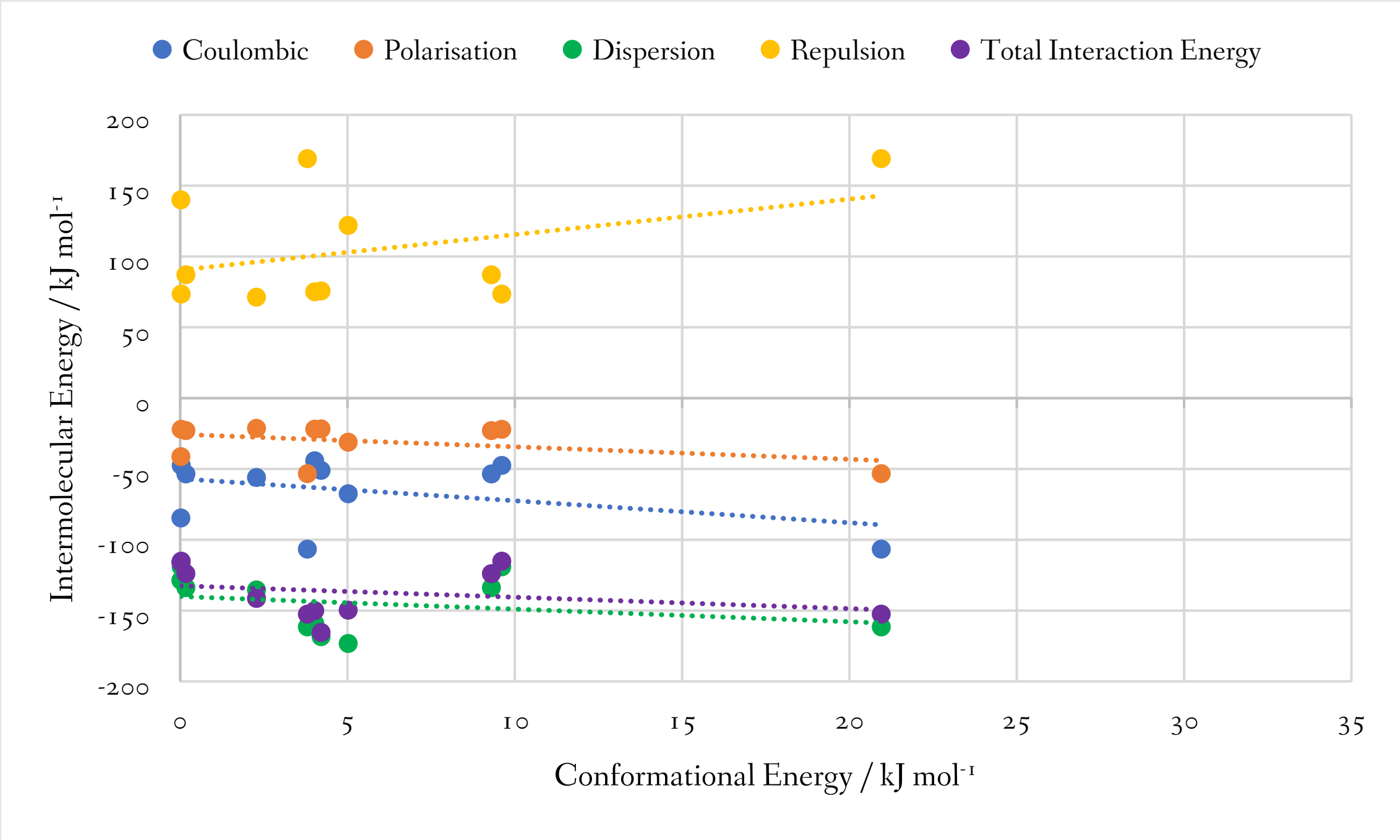

Figure S 40: Scatterplot of conventional SCXRD intermolecular interaction energies versus conformational energy.

Table S 35: Linear regression statistics for scatterplot of conventional SCXRD intermolecular interaction energies versus conformational energy.

	R^2-value	Standard Error	t-stat	P-value
E_{COU}	0.2325	1.1005	-1.6510	0.1331
E_{POL}	0.2547	0.5978	-1.7539	0.1133
E_{DISP}	0.1259	1.0119	-1.1384	0.2844
E_{REP}	0.2084	1.8707	1.5391	0.1582
E_{TOT}	0.1398	0.9197	-1.2092	0.2574

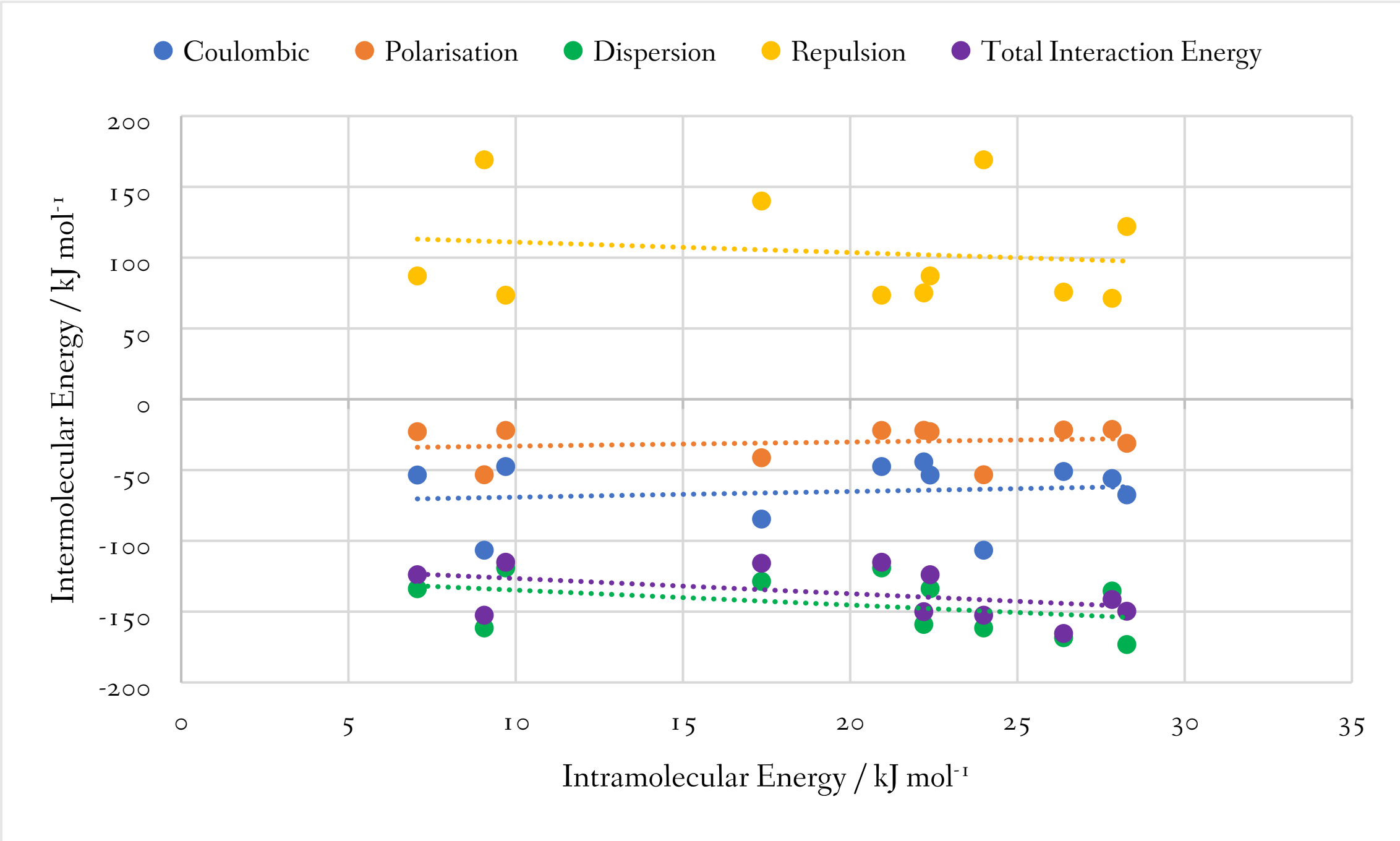

Figure S 41: Scatterplot of conventional SCXRD intermolecular interaction energies versus strain energy.

Table S 36: Linear regression statistics for scatterplot of conventional SCXRD intermolecular interaction energies versus strain energy.

	R^2-value	Standard Error	t-stat	P-value
E_{COU}	0.0519	1.1376	0.7022	0.5003
E_{POL}	0.0799	0.6178	0.8842	0.3996
E_{DISP}	0.1523	0.9269	-1.2716	0.2354
E_{REP}	0.0533	1.9028	-0.7117	0.4947
E_{TOT}	0.1787	0.8358	-1.3995	0.1952

11.2. Crystalline Sponge

11.2.1. Overview

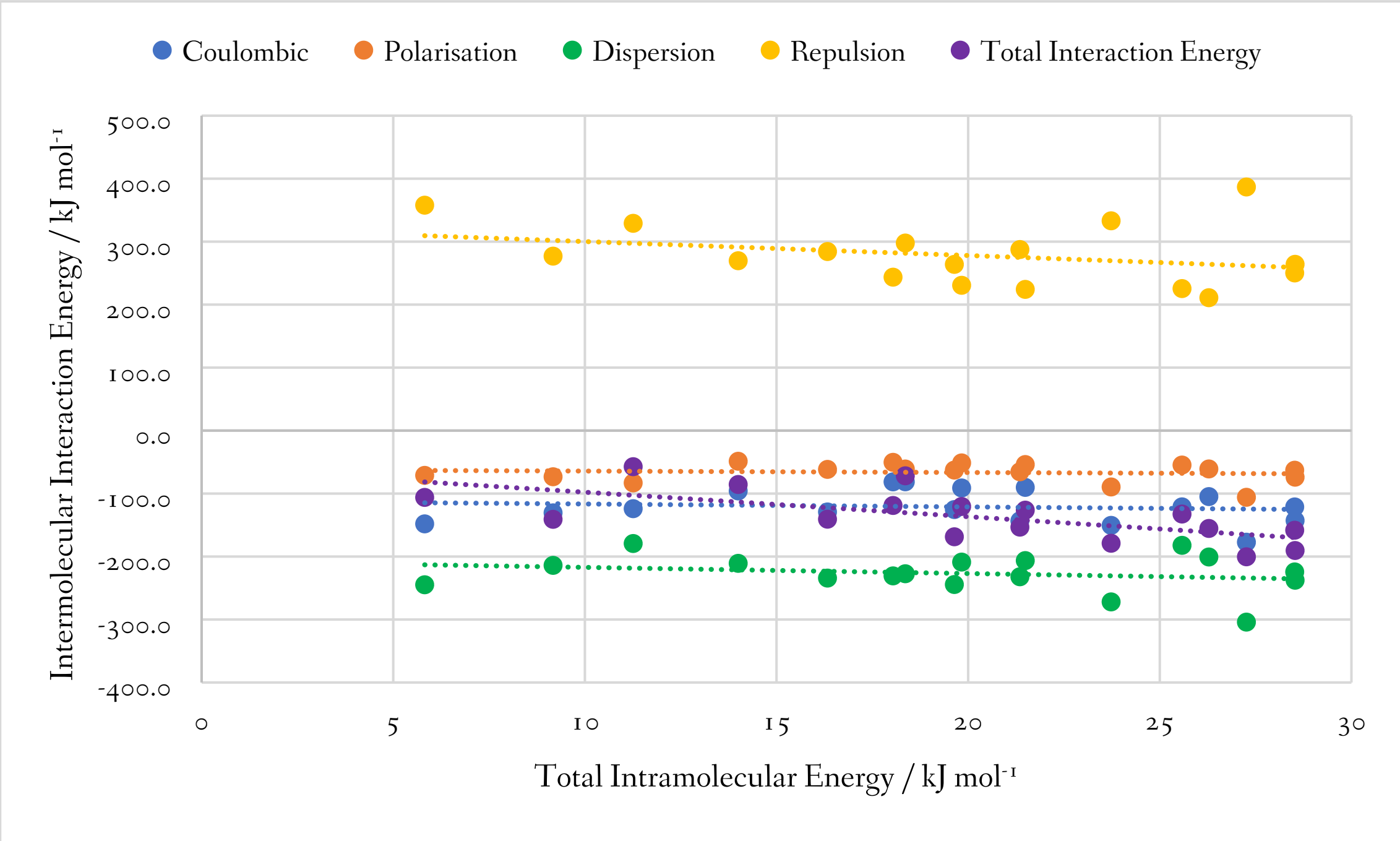

Figure S 42: Scatterplot of intermolecular interaction energies versus total relative energy for crystalline sponge structures.

Table S 37: Linear regression statistics for scatterplot of intermolecular interaction energies versus total relative energy for crystalline sponge structures.

	R^2-value	Standard Error	t-stat	P-value
E_{COU}	0.0134	1.0271	-0.4522	0.6576
E_{POL}	0.0105	0.5758	-0.3992	0.6954
E_{DISP}	0.0472	1.1362	-0.8616	0.4025
E_{REP}	0.0924	1.7945	-1.2355	0.2356
E_{TOT}	0.4350	1.1447	-3.3984	0.0040

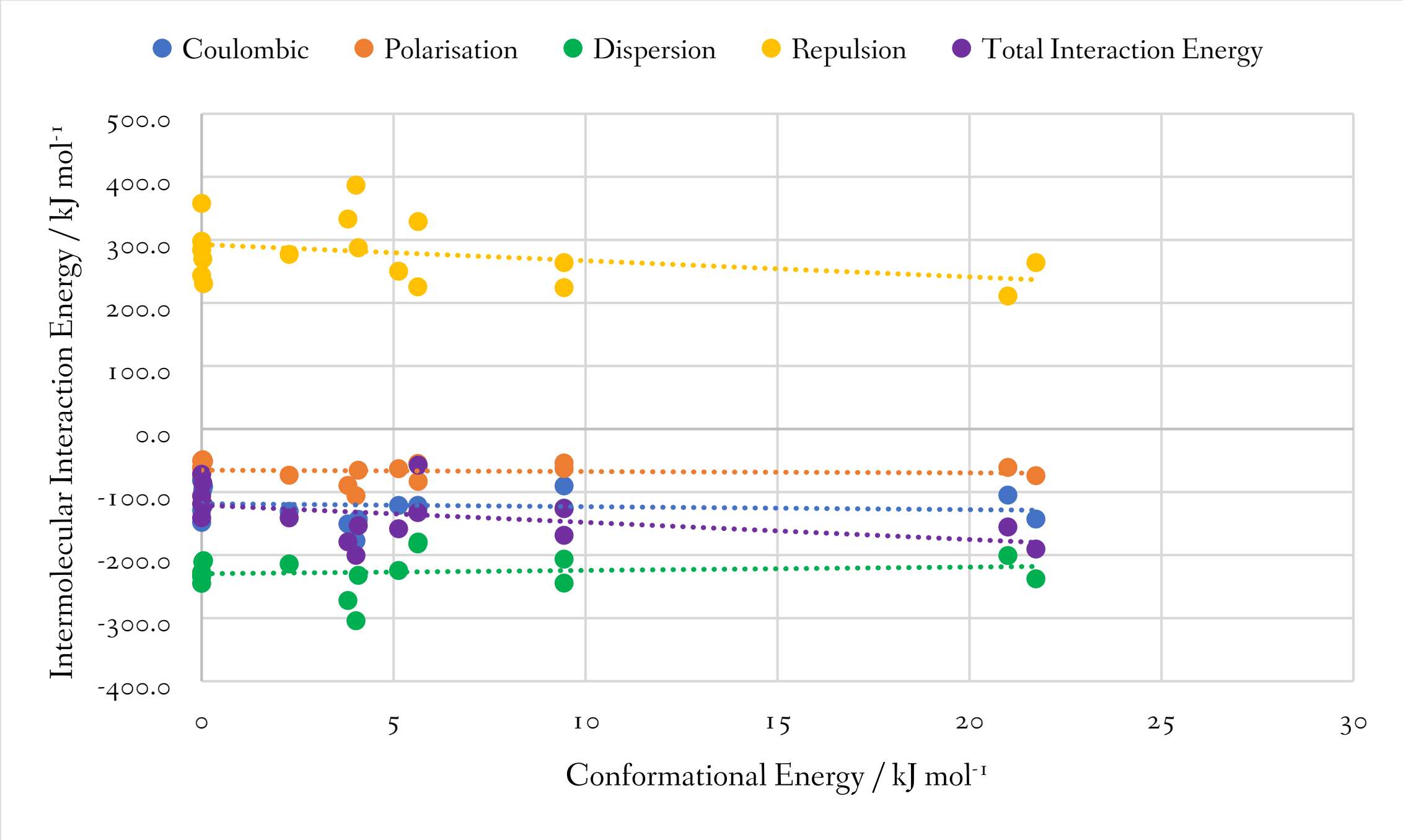

Figure S 43: Scatterplot of intermolecular interaction energies versus conformational energy for crystalline sponge structures.

Table S 38: Linear regression statistics for scatterplot of intermolecular interaction energies versus conformational energy for crystalline sponge structures.

	R^2-value	Standard Error	t-stat	P-value
E_{COU}	0.0139	1.0292	-0.4592	0.6527
E_{POL}	0.0089	0.5776	-0.3675	0.7184
E_{DISP}	0.0137	1.1585	0.4562	0.6547
E_{REP}	0.1227	1.7682	-1.4485	0.1681
E_{TOT}	0.2113	1.3555	-2.0047	0.0634

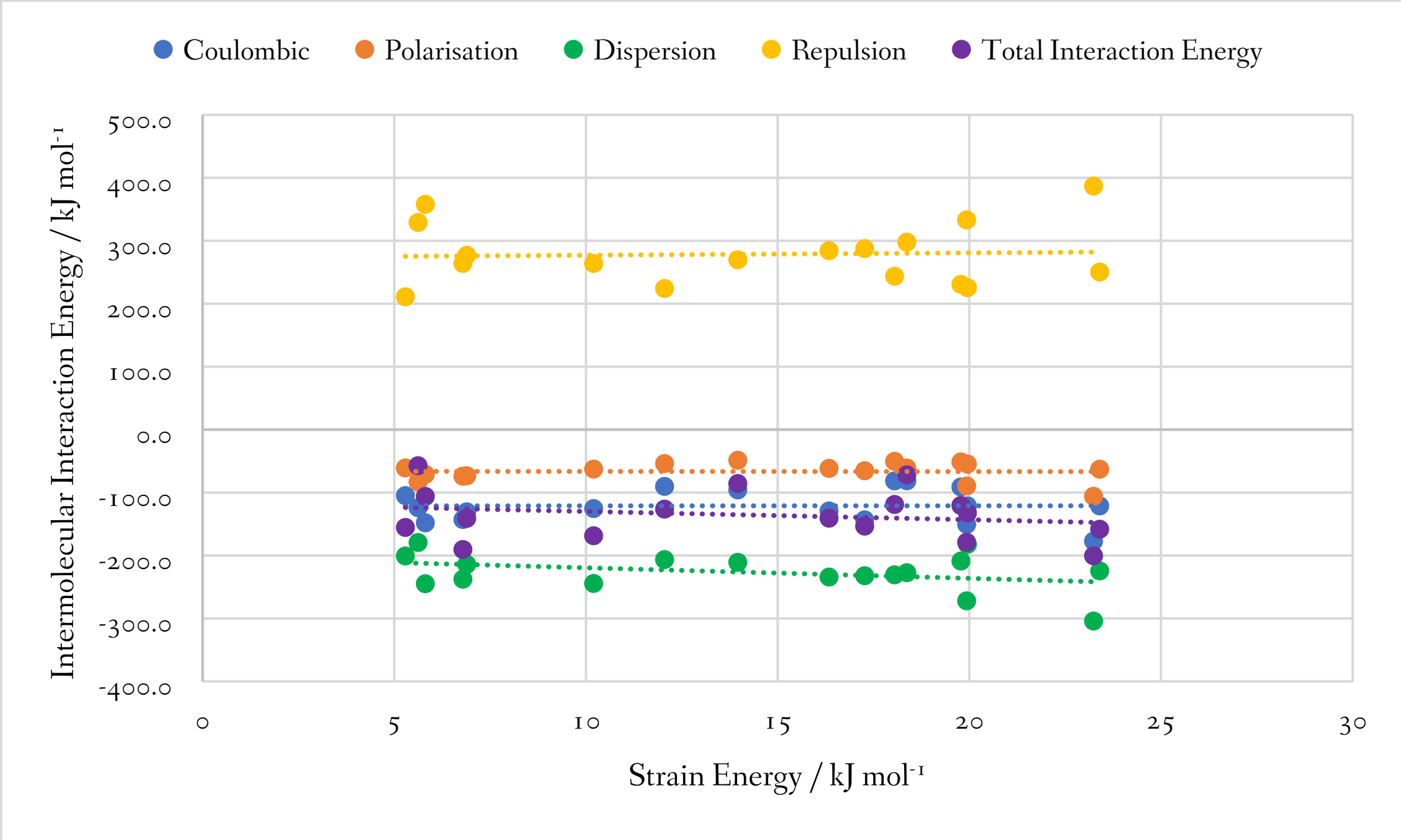

Figure S 44: Scatterplot of intermolecular interaction energies versus strain energy for crystalline sponge structures.

Table S 39: Linear regression statistics for scatterplot of intermolecular interaction energies versus strain energy for crystalline sponge structures.

	R^2-value	Standard Error	t-stat	P-value
E_{COU}	0.000004	1.0905	0.0073	0.9943
E_{POL}	0.000075	0.6104	-0.0336	0.9737
E_{DISP}	0.124014	1.1489	-1.4572	0.1657
E_{REP}	0.002291	1.9842	0.1856	0.8552
E_{TOT}	0.044927	1.5696	-0.8400	0.4141

11.2.2. Host – Guest and Guest – Guest Contributions

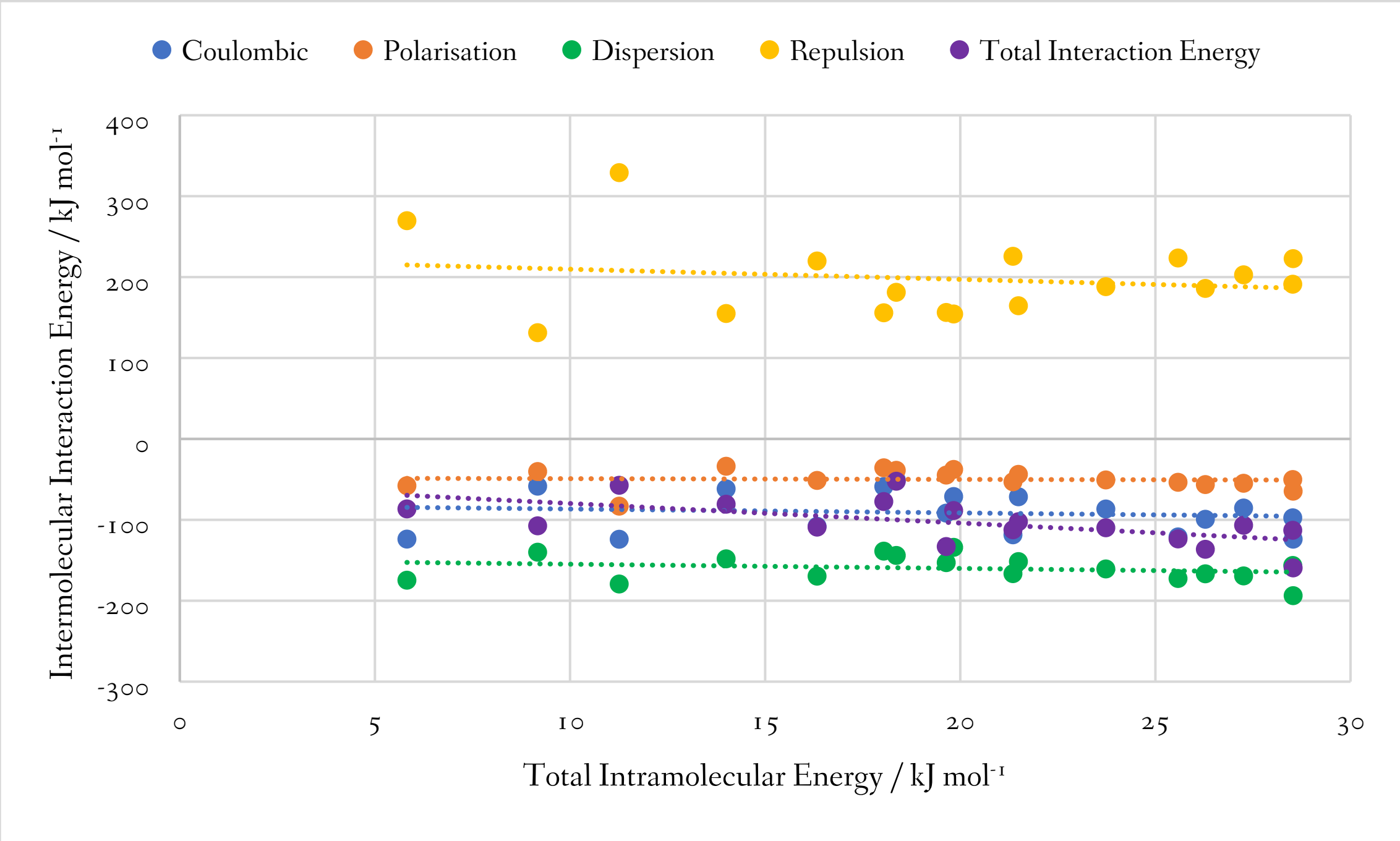

Figure S 45: Scatterplot of host – guest intermolecular interaction energies versus total relative energy for crystalline sponge structures.

Table S 40: Linear regression statistics for scatterplot of host – guest intermolecular interaction energies versus total relative energy for crystalline sponge structures.

	R^2-value	Standard Error	t-stat	P-value
E_{COU}	0.0159	0.9769	-0.4918	0.6300
E_{POL}	0.0026	0.4619	-0.1966	0.8468
E_{DISP}	0.0482	0.6019	-0.8713	0.3973
E_{REP}	0.0304	1.8329	-0.6858	0.5033
E_{TOT}	0.3515	0.8470	-2.8511	0.0121

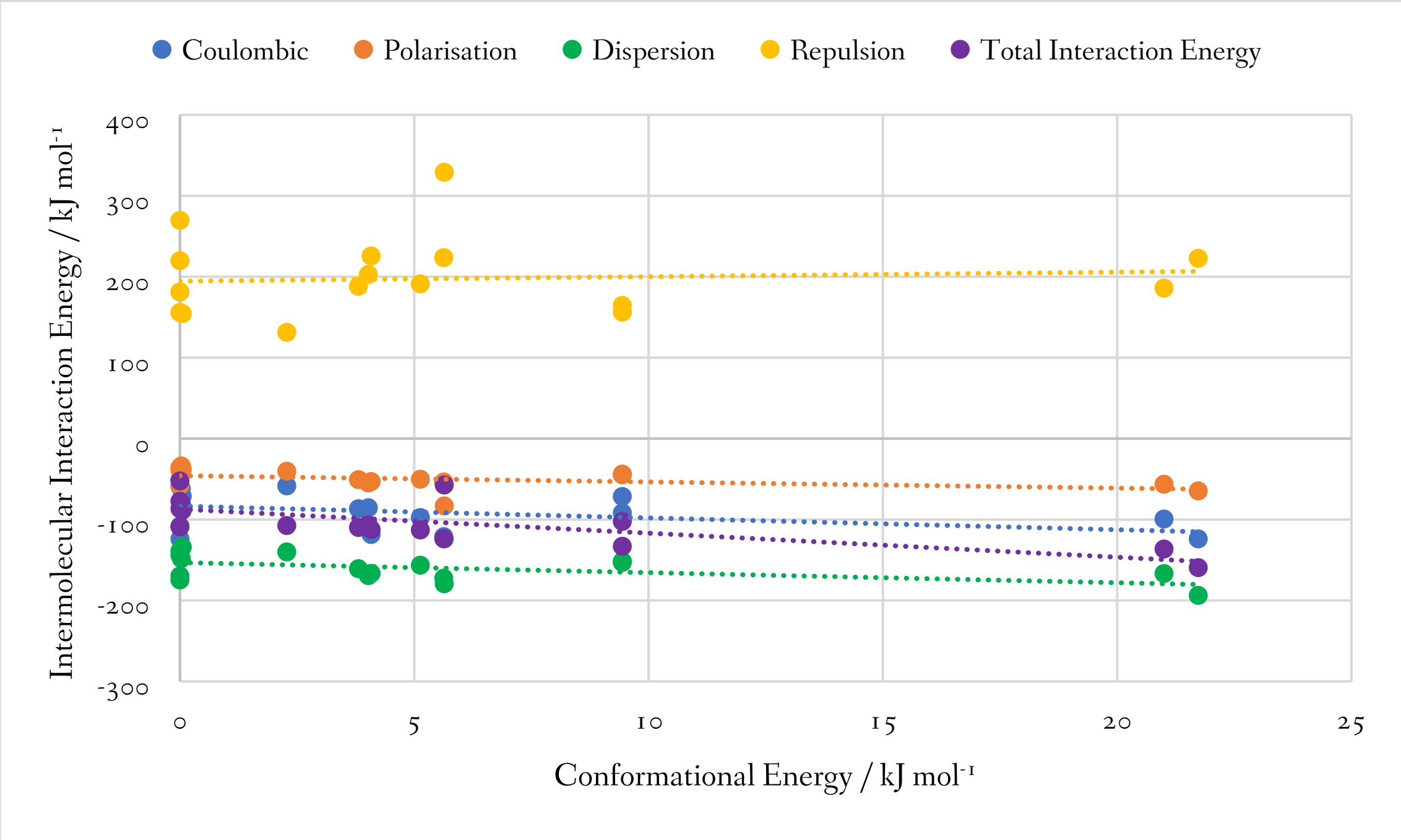

Figure S 46: Scatterplot of host – guest intermolecular interaction energies versus conformational energy for crystalline sponge structures.

Table S 41: Linear regression statistics for scatterplot of host – guest intermolecular interaction energies versus conformational energy for crystalline sponge structures.

	R^2-value	Standard Error	t-stat	P-value
E_{COU}	0.1457	0.9122	-1.5993	0.1306
E_{POL}	0.1814	0.4194	-1.8234	0.0882
E_{DISP}	0.2711	0.5279	-2.3622	0.0321
E_{REP}	0.0060	1.8599	0.3016	0.7671
E_{TOT}	0.5287	0.7236	-4.1024	0.0009

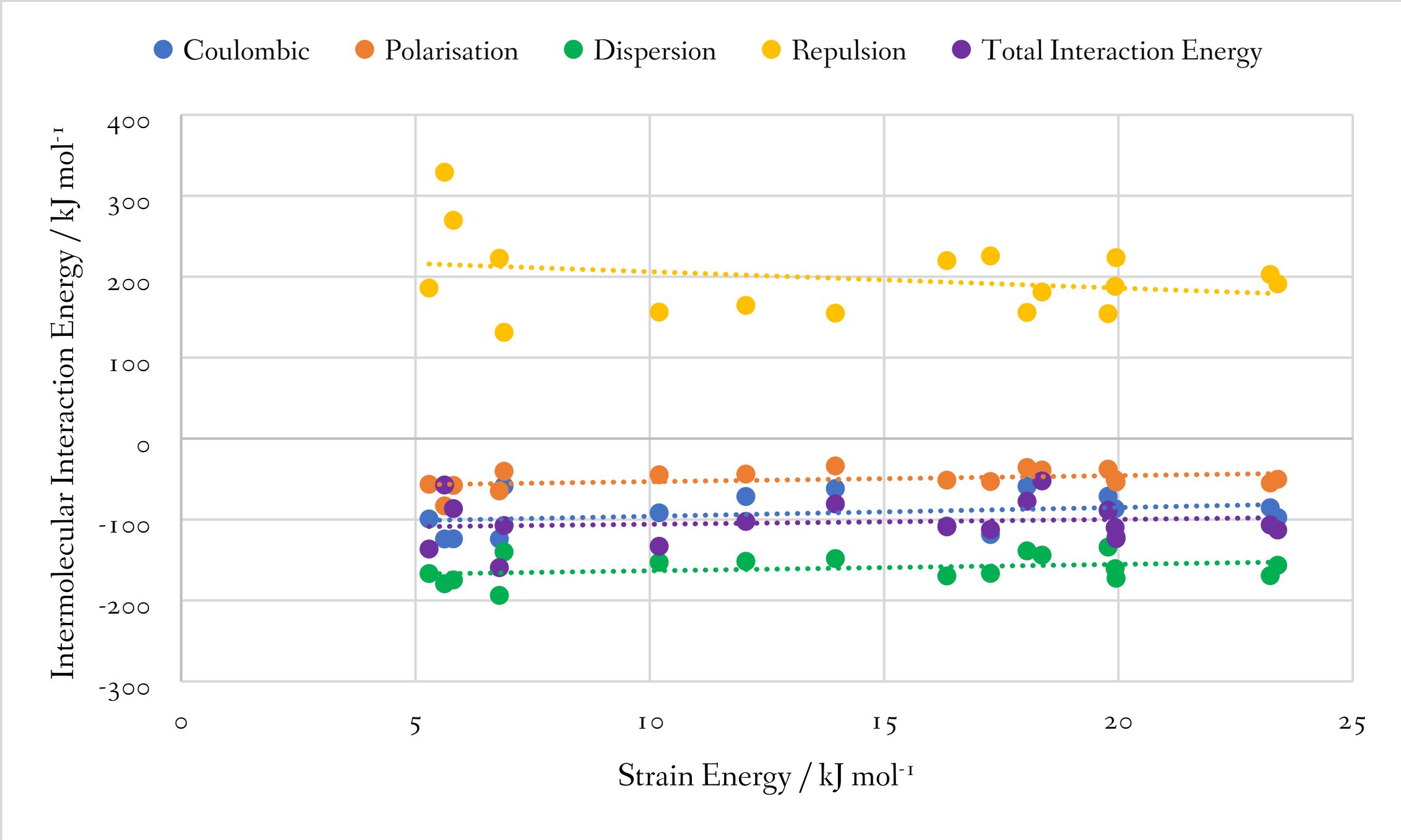

Figure S 47: Scatterplot of host – guest intermolecular interaction energies versus strain energy for crystalline sponge structures.

Table S 42: Linear regression statistics for scatterplot of host – guest intermolecular interaction energies versus strain energy for crystalline sponge structures.

	R^2-value	Standard Error	t-stat	P-value
E_{COU}	0.0724	1.0002	1.0821	0.2963
E_{POL}	0.1560	0.4481	1.6649	0.1167
E_{DISP}	0.1004	0.6171	1.2936	0.2154
E_{REP}	0.0707	1.8924	-1.0679	0.3024
E_{TOT}	0.0196	1.0982	0.5479	0.5918

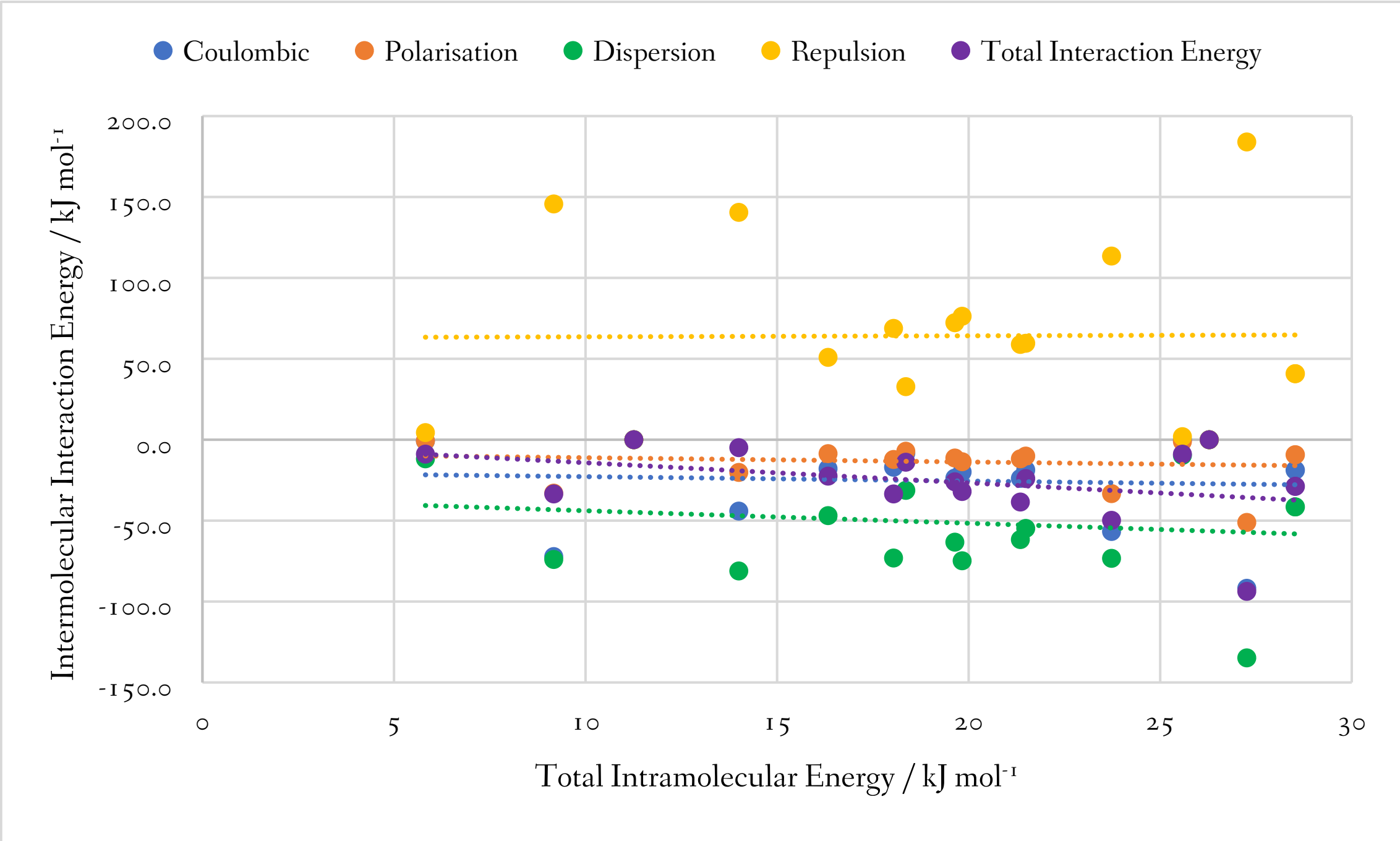

Figure S 48: Scatterplot of guest – guest intermolecular interaction energies versus total relative energy for crystalline sponge structures.

Table S 43: Linear regression statistics for scatterplot of guest – guest intermolecular interaction energies versus total relative energy for crystalline sponge structures.

	R^2-value	Standard Error	t-stat	P-value
E_{COU}	0.00485	0.9982	-0.2703	0.7906
E_{POL}	0.01698	0.5197	-0.5089	0.6182
E_{DISP}	0.02271	1.3075	-0.5904	0.5637
E_{REP}	0.00006	2.0737	0.0293	0.9770
E_{TOT}	0.14252	0.7888	-1.5790	0.1352

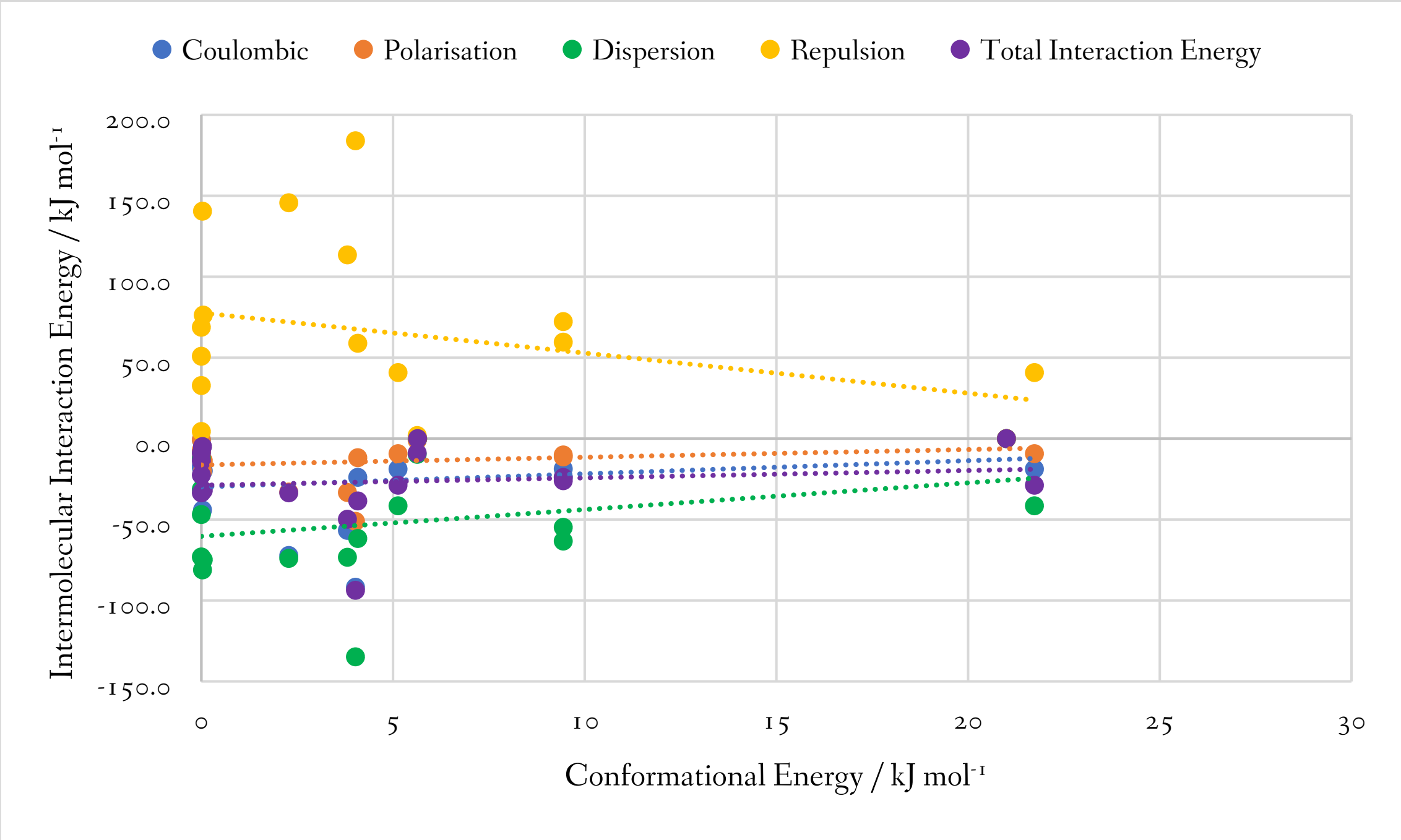

Figure S 49: Scatterplot of guest – guest intermolecular interaction energies versus conformational energy for crystalline sponge structures.

Table S 44: Linear regression statistics for scatterplot of guest – guest intermolecular interaction energies versus conformational energy for crystalline sponge structures.

	R^2-value	Standard Error	t-stat	P-value
E_{COU}	0.0435	0.9808	0.8257	0.4219
E_{POL}	0.0545	0.5108	0.9301	0.3670
E_{DISP}	0.1034	1.2552	1.3156	0.2081
E_{REP}	0.0951	1.9772	-1.2553	0.2286
E_{TOT}	0.0189	0.8457	0.5372	0.5990

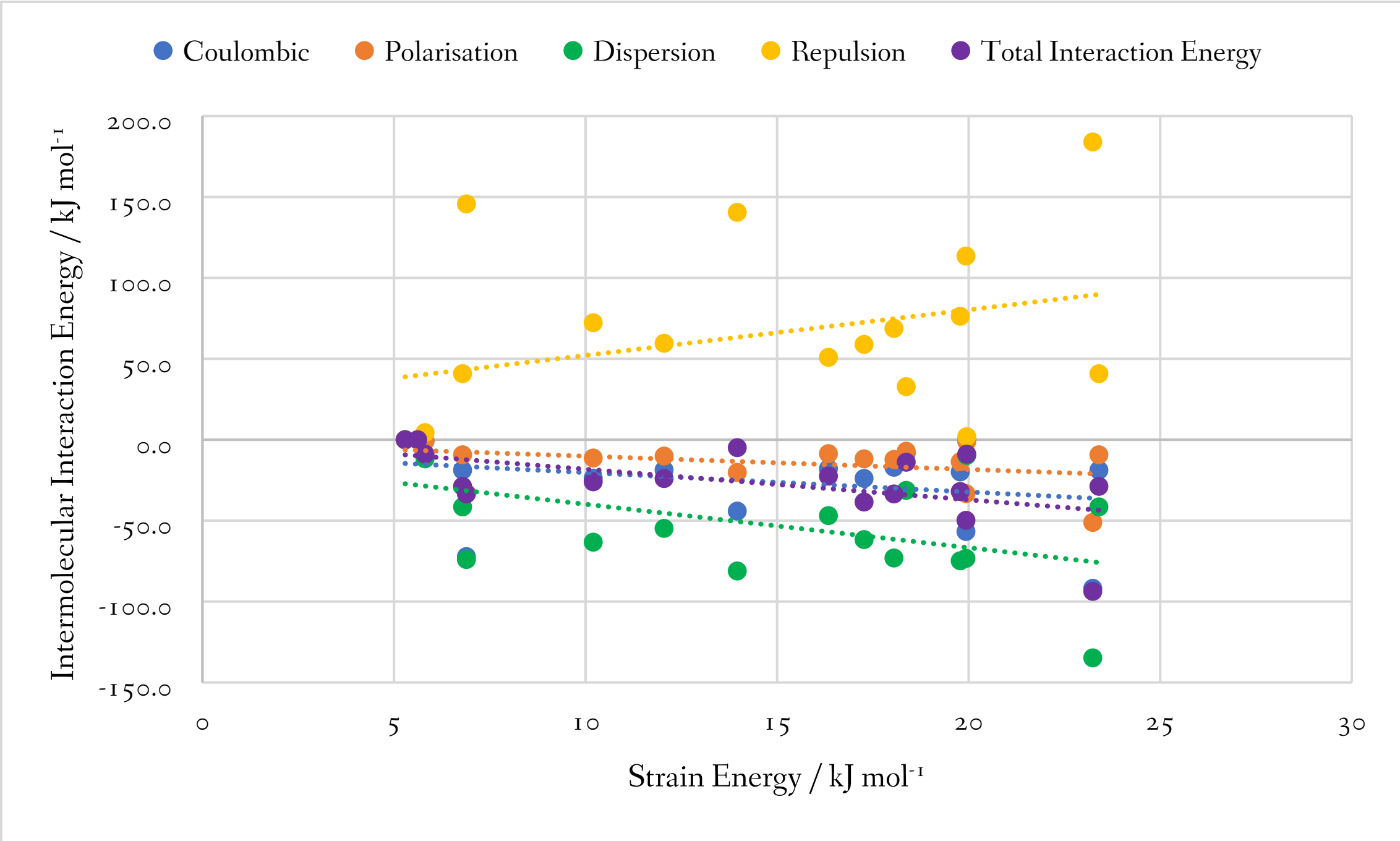

Figure S 50: Scatterplot of guest – guest intermolecular interaction energies versus strain energy for crystalline sponge structures.

Table S 45: Linear regression statistics for scatterplot of guest – guest intermolecular interaction energies versus strain energy for crystalline sponge structures.

	R^2-value	Standard Error	t-stat	P-value
E_{COU}	0.0857	1.0090	-1.1858	0.2542
E_{POL}	0.1468	0.5106	-1.6063	0.1290
E_{DISP}	0.2473	1.2101	-2.2200	0.0422
E_{REP}	0.1104	2.0627	1.3645	0.1925
E_{TOT}	0.2946	0.7545	-2.5032	0.0244

12. References

1 R. O. Diffraction, 2021.

2 O. V. Dolomanov, L. J. Bourhis, R. J. Gildea, J. A. K. Howard and H. Puschmann, *J. Appl. Cryst.*, 2009, **42**, 339–341.

3 G. M. Sheldrick, *Acta Crystallogr.*, 2015, 3–8.

4 G. M. Sheldrick, *Acta Crystallogr.*, 2015, **C71**, 3–8.

5 A. R. Tyler, R. Ragbirsingh, C. J. McMonagle, P. G. Waddell, S. E. Heaps, J. W. Steed, P. Thaw, M. J. Hall and M. R. Probert, *Chem*, 2020, **6**, 1755–1765.

6 C. F. Macrae, I. Sovago, S. J. Cottrell, P. T. A. Galek, P. McCabe, E. Pidcock, M. Platings, G. P. Shields, J. S. Stevens, M. Towler and P. A. Wood, *J. Appl. Crystallogr.*, 2020, **53**, 226–235.

7 M. G. Reeves, P. A. Wood and S. Parsons, *J. Appl. Crystallogr.*, 2020, **53**, 1154–1162.

8 M. J. Frisch, G. W. Trucks, H. B. Schlegel, G. E. Scuseria, M. A. Robb, J. R. Cheeseman, G. Scalmani, V. Barone, G. A. Petersson, H. Nakatsuji, X. Li, M. Caricato, A. Marenich, J. Bloino, B. G. Janesko, R. Gomperts, B. Mennucci, H. P. Hratchian, J. V. Ortiz, A. F. Izmaylov, J. L. Sonnenberg, D. Williams-Young, F. L. F. Ding, J. G. F. Egidi, A. P. B. Peng, T. Henderson, D. Ranasinghe, V. G. Zakrzewski, N. R. J. Gao, G. Zheng, W. Liang, M. Hada, M. Ehara, K. Toyota, R. Fukuda, J. Hasegawa, M. Ishida, T. Nakajima, Y. Honda, O. Kitao, H. Nakai, T. Vreven, K. Throssell, J. A. Montgomery, J. E. P. Jr., F. Ogliaro, M. Bearpark, J. J. Heyd, E. Brothers, K. N. Kudin, V. N. Staroverov, T. Keith, R. Kobayashi, J. Normand, K. Raghavachari, A. Rendell, J. C. Burant, S. S. Iyengar, J. Tomasi, M. Cossi, J. M. Millam, M. Klene, C. Adamo, R. Cammi, J. W. Ochterski, R. L. Martin, K. Morokuma, O. Farkas, J. B. Foresman and D. J. Fox, 2016.

9 A. G. P. Maloney, P. A. Wood and S. Parsons, *CrystEngComm*, 2015, **17**, 9300–9310.

10 A. G. P. Maloney, P. A. Wood and S. Parsons, *CrystEngComm*, 2016, **18**, 3273–3281.
